# A novel synthetic approach to hydroimidazo[1,5-*b*]pyridazines by the recyclization of itaconimides and HPLC–HRMS monitoring of the reaction pathway

**DOI:** 10.3762/bjoc.13.252

**Published:** 2017-11-30

**Authors:** Dmitry Yu Vandyshev, Khidmet S Shikhaliev, Andrey Yu Potapov, Michael Yu Krysin, Fedor I Zubkov, Lyudmila V Sapronova

**Affiliations:** 1 Faculty of Chemistry, Voronezh State University, Universitetskaya sq., 1, Voronezh 394018, Russian Federation; 2 Faculty of Physics and Mathematics and Natural Sciences, RUDN University, Miklukho-Maklaya St., 6, Moscow 117198, Russian Federation

**Keywords:** cascade reaction, diaminoimidazoles, HPLC–HRESIMS, imidazo[1,5-*b*]pyridazines, itaconimides

## Abstract

The novel cascade two-stage reaction between itaconimides and 1,2-diamino-4-phenylimidazole proceeds regio- and chemoselectively to form tetrahydroimidazo[1,5-*b*]pyridazines and includes nucleophilic C-addition by the activated C=C double bond and subsequent intramolecular recyclization of the intermediate with the amino group involved.

## Introduction

Among the numerous bicyclic fused imidazole derivatives, there is great interest in imidazo[1,5-annelated]diazines due to their diverse pharmacological actions and bioisosterism with imidazo[4,5-*d*]pyrimidines (purines). The presence of a bridgehead nitrogen atom is a common structural motif of these heterocyclic systems. For example, compounds with the imidazo[1,5-*a*]pyrazine structure show inhibitory activity against kinases BTK [1], MEK [2], ACK1 [3], mTORC1(2) [4], c-Src [5], growth factor IGF-1R [6] and act as the antagonists of Hedgehog pathway dependent malignancies [7]. Imidazo[1,5-*a*]pyrimidines are inhibitors of the bone morphogenic protein [8], antitumor agents [9], and are stimulators of guanylate cyclase [10], whereas imidazo[1,5-*c*]pyrimidines demonstrate anti-infectious effects in the treatment of brucellosis [11], etc. However, the chemistry, medicinal chemistry and pharmacology of imidazo[1,5-*b*]pyridazines, with the imidazole ring connected through the N1–C5 bond of the latter, have been studied to a lesser extent. So, for imidazo[1,5-*b*]pyridazines, inhibitory activity was found against the viruses of human immunodeficiency HIV-1 [12], influenza [13], and hepatitis C [14]. They also act as stimulators of guanylate cyclase [10], inhibitors of phosphodiesterase 10A [15], protease-activated receptor 1 [16], PIM-1/2 kinase [17], antagonists of the corticotropin releasing factor [18–19], vanilloid-1 receptor [20], and modulators for ligand binding to GABA*_A_* receptor [21]. As pyridazines are considered as privileged structures in drug design [22], we decided to search for new reagents for the synthesis of their imidazo[1,5-*b*]-annelated analogues.

**Scheme 1:**
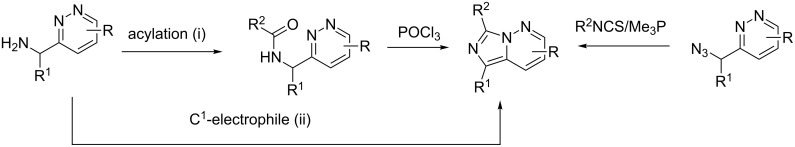
Intramolecular cyclization of 3-(aminomethyl)pyridazines and related compounds (route A). Conditions: i) R^2^COCl/Et_3_N [12], R^2^COOH/DCC [13,23], (R^2^CO)_2_O/Et_3_N [19]; ii) synthetic equivalent of C^1^-electrophile: R^2^COOH/T3P^®^ [15], BrCN (R^2^ = NH_2_) [16], ArNCS/DCC (R^2^ = NHAr) [20].

**Scheme 2:**
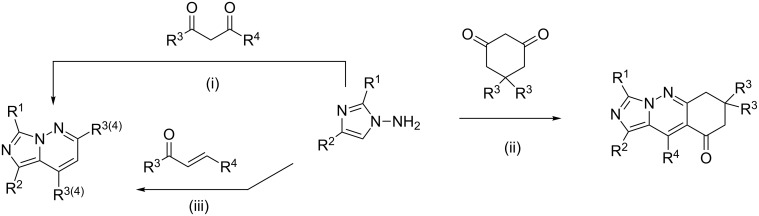
Heterocyclization of 1-aminoimidazoles with 1,3-dicarbonyl or α,β-unsaturated carbonyl compounds (route B). Conditions: i) R^1^ = NH_2_, NHAlk, R^2^ = Ph, R^3,4^ = Alk, Ar, solvent-free [24], AcOH [25–26], R^3^ = Ph, R^4^ = OEt, solvent-free [24]; ii) R^1^ = NH_2_, R^2^ = Ph, R^3^ = H, Me, R^4^ = Ar, ArCHO/MeOH/DMF [29] or R^4^ = H, HC(OEt)_3_/iPrOH [30]; iii) R^1^ = NH_2_, R^2^ = Ar, R^3,4^ = Alk, Ar, MeOH/*N*-methylmorpholine or DMF or AcOH [27–28], R^3^ = Ar, R^4^ = COOH, DMF [31], R^3^ = Ar, R^4^ = NMe_2_, AcOH/DMF [32].

**Scheme 3:**
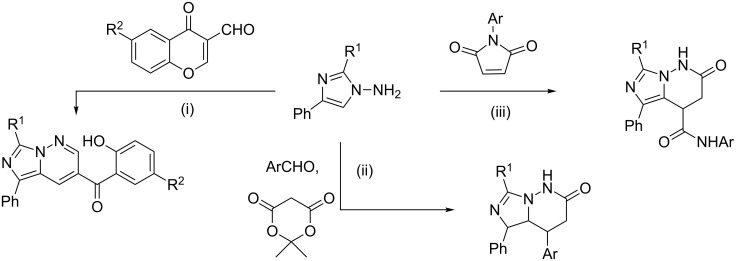
Heterocyclization of 1-aminoimidazoles with structural transformation of dielectrophilic reagents (route B). Conditions: i) R^1^ = NH_2_, SH, R^2^ = H, Me, MeO, F, Me_3_SiCl/DMF [33]; ii) R^1^ = NH_2_, MeOH/DMF [34]; iii) R^1^ = NH_2_, AcOH/iPrOH [35].

There are two general approaches to the construction of an imidazo[1,5-*b*]pyridazine scaffold: the annulation of the imidazole ring to the b-bond of the pyridazine (route A, Scheme 1) and the heterocyclization of 1-aminoimidazoles to build the pyridazine core (route B, Scheme 2 and Scheme 3).

Following route A (Scheme 1), the aminomethylpyridazine derivatives are either pre-acylated and subsequently cyclized with phosphorus oxychloride [12–13,19,23], or the formation of imidazo[1,5-*b*]pyridazines occurs as a result of their direct heterocyclization involving various single-carbon electrophilic reagents [15–16,20]. A similar one-pot aza-Staudinger reaction of 3-(azidomethyl)pyridazines with isothiocyanates in the presence of PMe_3_ also results in imidazopyridazines [17].

In the synthesis of imidazo[1,5-*b*]pyridazines along route B, 1-aminoimidazoles with no substituents at the C-5 atom of the original heterocycle and 1,3-dielectrophilic reagents are used, e.g., 1,3-diketones [24–26], β-ketoesters [24], α,β-unsaturated ketones [27–28], including those obtained in situ [29–30] or containing good leaving groups [31–32] (Scheme 2).

In the reactions of 1-aminoimidazoles with cyclic 1,3-dielectrophilic reagents (3-formylchromones [33], Meldrum’s acid derivatives [34], and *N*-arylmaleimides [35]), transformation of the structure of the latter ones is often observed (Scheme 3).

Among these methods the heterocyclization (method B) of 1-aminoimidazoles seems more attractive due to their better accessibility when compared to the corresponding pyridazines required in route A. Further, by careful selection of the reactants functional diversification of the target imidazopyridazines over virtually any carbon atom of the heterocyclic system is achievable in addition to hydrogenated imidazo[1,5-*b*]pyridazines [34–35].

**Scheme 4:**
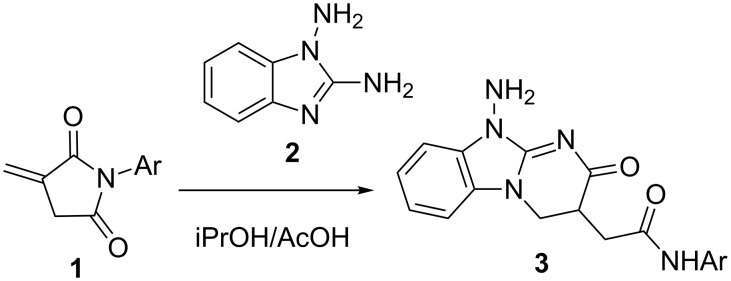
Recyclization of *N*-arylitaconimides **1** with 1,2-diaminobenzimidazole (**2**).

Itaconimides, in contrast to itaconic acid, its esters, monoamides or anhydride [36–37], scarcely have been studied in the synthesis of heterocyclic compounds. The presence of the exocyclic activated C=C double bond allows itaconimides to react with heteroaromatic dinucleophiles as dielectrophilic reagents with the possibility of recyclization. Recently, our group reported the first example of this recyclization with *N*-arylitaconimides **1** and 1,2-diaminobenzimidazole (**2**) as 1,3-N,N-dinucleophile, leading to tetrahydropyrimido[1,2-*a*]benzimidazoles **3** (Scheme 4) [38].

In continuation of our studies on the synthesis of azolo-annelated heterocycles we herein report the results of our investigations on the reactions between *N*-arylitaconimides and 1,2-diamino-4-phenylimidazole as a heterocyclic C,N/N,N polynucleophile.

## Results and Discussion

Our earlier studies and literature information have shown that acetic acid significantly accelerates the reactions of 1,2-diaminoimidazole with 1,3-dielectrophilic reagents. Therefore, the heterocyclization of *N*-arylitaconimides **1a**–**g** and diaminoimidazole **4** was carried out in refluxing iPrOH in the presence of catalytic amounts of AcOH. In all reactions only one compound was isolated in good yields (Table 1). Attempts to replace the alcoholic solvent with dioxane or DMF resulted in decreased yields of the target products.

**Table 1:** Yields of the interaction products **9** of *N*-arylitaconimides **1a**–**g** and diaminoimidazole **4**.

entry	itaconimide	Ar	product	yield (%)

1	**1a**	4-MeC_6_H_4_	**9a**	62
2	**1b**	4-EtC_6_H_4_	**9b**	60
3	**1c**	3-ClC_6_H_4_	**9c**	70
4	**1d**	4-ClC_6_H_4_	**9d**	72
5	**1e**	3,4-Me_2_C_6_H_3_	**9e**	65
6	**1f**	3,5-Me_2_C_6_H_3_	**9f**	67
7	**1g**	3,4-Cl_2_C_6_H_3_	**9g**	66

**Scheme 5:**
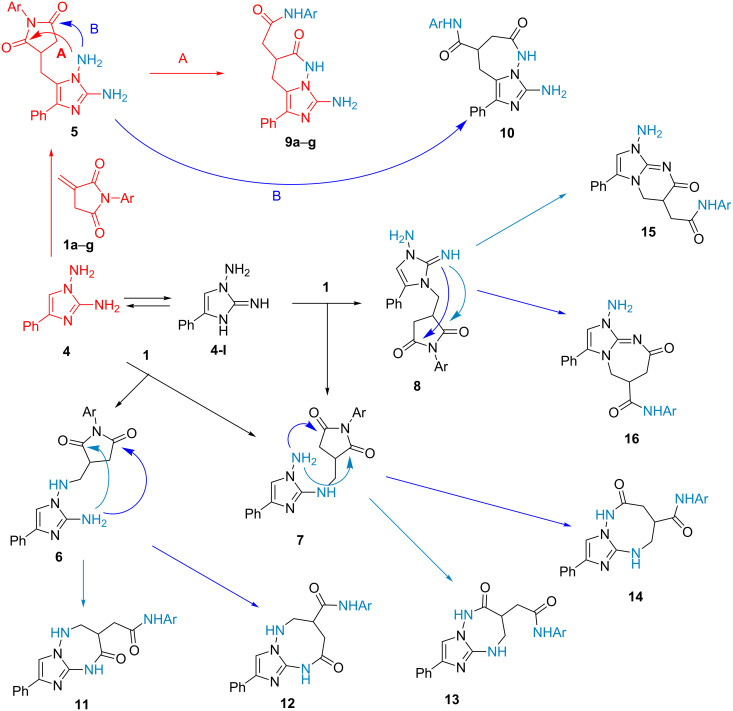
Possible synthetic routes of the interaction of itaconimides **1** with diaminoimidazole **4**.

The problem of chemoselectivity and regioselectivity of the reactions under study is associated with the polynucleophilicity of 1,2-diamino-4-phenylimidazole (**4**), whose structure contains several dinucleophilic centers, namely: 1,3-C,N (C^5^-N^1^-NH_2_), 1,3-N,N (HN^3^-C^2^=NH in the other tautomeric form **4-I**), 1,4-N,N (NH_2_-N^1^-C^2^-NH_2_) as well as with the polyelectrophilicity of itaconimides **1** due to the presence of three electrophilic C atoms: the terminal atom of the exocyclic activated multiple bond and two atoms of the imide group. Due to this, at the first step of the reaction between diaminoimidazole and itaconimides, four primary adducts **5**–**8** could result (Scheme 5) based on the well-known fact that the heterocyclization reactions of α,β-unsaturated carbonyl compounds with dinucleophilic reagents typically begin with addition like Michael’s reaction, including heterocyclic dinucleophiles [39–42]. Mechanistically, further intramolecular recyclization of the succinimide fragments in intermediates **5**–**8** could proceed by any of the two imide C atoms (Scheme 5).

Thus, for products **5** of the reaction of diaminoimidazole **4** as a 1,3-C,N-dinucleophile in the routes A and B, imidazo[1,5-*b*]pyridazines **9** and imidazo[1,5-*b*][1,2]diazepines **10**, respectively, can be obtained. As a result of the initial addition of diaminoimidazole **4** as a 1,4-N,N-dinucleophile, intermediates **6** and **7** could be formed, whose heterocyclization with a second amino group could lead to imidazotriazepines **11** and **13** or imidazotriazocines **12** and **14**. The capability of diaminoimidazoles in their tautomeric form **4-I** to react as 1,3-N,N-dinucleophiles [38] determines the possible formation of adducts **8**, whose recyclization could give imidazopyrimidines **15** or imidazodiazepines **16**. The amine–imine tautomeric equilibrium is characteristic of aminoazole systems containing an amino group at the second position of the cycle [43]. The heterocyclization of intermediate **6** involving the C5 atom of the imidazole is improbable (not shown in the Scheme 5).

The key criterion for the choice of the structure of the compounds obtained is the presence of the signals of four protons associated with nitrogen atoms in their ^1^H NMR spectra: the broad singlet of the NH_2_ group in the region of 5.6–5.7 ppm and those of the two amide NH groups in the region of 10.0–11.5 ppm. This set of signals unambiguously excludes the possible structures of both intermediates **6**–**8** and their recyclization products **11**–**16**, as these molecules contain only three hydrogen atoms in their nitrogen-containing functional groups.

**Scheme 6:**
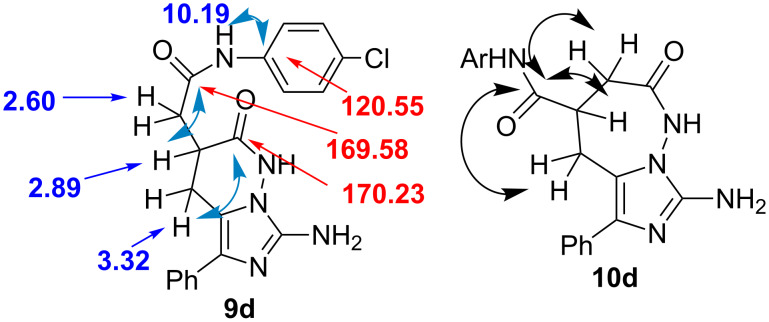
^1^H,^13^C-HMBC correlations: the most significant correlations for imidazopyridazine **9d** and possible for imidazodiazepine **10d**.

The final choice in favor of the formation of imidazopyridazines **9a**–**g** is based on the correlations found in the ^1^H,^13^C-HMBC NMR spectrum of compound **9d** (Scheme 6). To confirm the presence of the last six-membered pyridazine ring in the structure and, accordingly, the exocyclic acetanilide residue, it is first of all necessary to make a strict assignment of the carbonyl carbon atom signals at around 170 ppm.

In the ^1^H NMR spectra of imidazopyridazines **9a**–**g**, there are two N–H singlets: a narrow one at 10.19 ppm and a broadened at 11.35 ppm. The type of signals allows making an assumption that the latter refers to a cyclic lactam fragment whose hydrogen atoms are sufficiently mobile. However, only for the narrow singlet there are cross peaks with a carbonyl C atom at 169.58 ppm and the carbon atom of the benzene ring (120.55 ppm) in the ^1^H,^13^C-HMBC spectrum. For the last aromatic carbon atom, there is also a correlation with the proton of the ring (7.59 ppm). Therefore, the above mentioned C-carbonyl is highly likely to belong to the amide group. Moreover, for this atom there are also two correlations with one of the diastereotopic protons of the exocyclic methylene group, whose signal (~2.60 ppm) overlaps with the residual proton signals of DMSO-*d*_6_, and with the proton of the methine group (2.89 ppm), which is partially overlapped by the signal of water present in the solvent. As no cross-peaks with protons of another methylene group are observed due to a significant number of bonds between them, the acetanilide moiety must be in the exo-position to the pyridazine ring. One of the remaining protons (3.32 ppm) correlates with the carbonyl C atom (170.23 ppm) of the lactam fragment. In the alternative structure of imidazodiazepine **10d**, the amide carbon atom would correlate with the protons of both methylene as well as methine groups (Scheme 6).

A possible confirmation of the formation of imidazopyridazine **9d** would be the correlation between protons of the exo- and endocyclic methylene groups in the NOESY experiment. However, the corresponding cross peaks were not detected, due to the spatial remoteness of these hydrogen atoms.

The registration of the ^1^H NMR spectrum of imidazopyridazine **9d** in the presence of trace amounts of CF_3_COOH allowed for a better assignment of the aliphatic protons due to a change in the position and intensity of signals of those protons associated with heteroatoms capable of protonation. This also included protons of water present in the DMSO-*d*_6_. Thus, doublets of doublets were observed for the diastereotopic protons of the exocyclic methylene group at 2.64 ppm (^2^*J* = 15.9 Hz, ^3^*J* = 6.7 Hz) and 2.87 ppm (^2^*J* = 15.9 Hz, ^3^*J* = 5.9 Hz) in addition the protons of the endocyclic methylene group at 3.09 ppm (^2^*J* = 15.6 Hz, ^3^*J* = 10.6 Hz) and 3.32 ppm. (^2^*J* = 15.6 Hz, ^3^*J* = 5.5 Hz). The signal of the methine proton was a multiplet (3.17–3.25 ppm). The addition of trifluoroacetic acid did not have a significant effect on the position of the signals of these protons.

Thus, the reaction of itaconimides **1** with diaminoimidazole **4** is a regioselective and chemoselective cascade process involving an initial C-addition of diaminoimidazole as a 1,3-C,N-dinucleophile to the activated C=C double bond to form intermediate **5** followed by recyclization involving the N^1^-amino group which leads to the formation of imidazo[1,5-*b*]pyridazines **9**.

Monitoring of the liquid phase composition of the reaction mixture by HPLC–HRMS showed that during the reaction of itaconimide **1d** with diaminoimidazole **4**, four compounds result with the integer mass of the protonated molecular ion (*m*/*z* 396 [M + H]^+^), which corresponds to the possible products of the reagent interaction (Table 2).

**Table 2:** Results of HPLC–HRESIMS monitoring of the reaction mixture composition in the synthesis of imidazopyridazine **9d**.

entry	compound	[M + H]^+^ calcd *m*/*z*	[M + H]^+^ found *m*/z	*t*_R_^a^, min	composition of the reaction mixture, % (time after reaction start)				
					
					10 min	11 min	16 min	30 min	60 min

1	**1d**	222.0317	222.0314	5.6	0.7	0.4	–	–	–
2	**4**	175.0979	175.0977	1.5	81.2	83.3	81.2	77.6	79.9
3	**5**–**8d**^b^	396.1223	396.1225	3.6	2.0	1.9	1.7	2.1	2.2
4	**5**–**8d**^b^	396.1223	396.1225	3.8	5.3	5.1	7.8	10.5	11.2
5	**9d**^c^	396.1223	396.1224	4.2	10.8	8.8	8.6	7.4	3.5
6	**10d**^d^	396.1223	396.1224	5.3	–	0.5	0.7	2.4	3.2

^a^Retention time (*t*_R_), average value; ^b^one of possible intermediates **5**–**8d**; ^c^for isolated compound **9d**, the retention time is 4.13 min; ^d^imidazodiazepine **10d** or one of the possible products of recyclization of intermediates **6**–**8d**.

However, it is still impossible to give a full assessment of the probable routes of the cascade recyclization process, because ions of the protonable substances are only fixed in the given ESI–MS conditions, and precipitation of the product is observed as the reaction proceeds. The latter causes a decreased content of the imidazopyridazine **9d** in the liquid phase is observed, whose peak is identified by the retention time (4.13 min) determined for the pure substance. The long retention time (5.6 min) and the insignificant content (less than 1%) of the initial itaconimide **1d** found under ESI conditions in the reaction mixture are due to its extremely low proton affinity. Allowing for the formal structural similarity of intermediates **5**–**8d**, we assume that one of the chromatographic peaks with the retention time of 3.6 or 3.8 min corresponds to the intermediate diaminoimidazole **5d**, and the second one corresponds to one of succinimides **6**–**8d**. The accumulation of the compound exhibiting a retention time of 5.3 min occurs about 30 min after the beginning of the reaction. In our opinion, this minor product is either imidazodiazepine **10d** or one of the possible products **11**–**16d** of recyclization of intermediates **6**–**8d** due to their more complex structure compared with the structure of the latter ones, and, correspondingly, the lower chromatographic mobility. However, the formation of heterocyclic systems including 7- and 8-membered rings is unlikely, because of the spatial remoteness of the corresponding reaction centers in the recyclization process. Nevertheless, the results of the HPLC–HRESIMS monitoring of the reaction confirm its selectivity.

## Conclusion

In summary, a new regioselective and chemoselective cascade reaction of *N*-arylitaconimides with 1,2-diamino-4-phenylimidazole as 1,3-C,N-dinucleophile was developed to synthesize tetrahydroimidazo[1,5-*b*]pyridazines. The process includes the steps of Michael’s initial C-addition of diaminoimidazole to the activated multiple bond of the imide followed by recyclization of the primary adducts. The availability of the reagents needed, the simplicity of the synthetic procedures, and the possibility of further functionalization of the hydrogenated heterocyclic scaffold imidazo[1,5-*b*]pyridazine are the major advantages of the developed reaction.

## Supporting Information

File 1 Experimental procedures, characterization data, copies of ^1^H, ^13^C spectra of the products and results of HPLC–HRESIMS monitoring of the reaction mixture composition.

## References

[1] Liu J, Guiadeen D, Krikorian A, Gao X, Wang J, Boga S B, Alhassan A-B, Yu Y, Vaccaro H, Liu S (2016). ACS Med Chem Lett.

[2] Robarge K D, Lee W, Eigenbrot C, Ultsch M, Wiesmann C, Heald R, Price S, Hewitt J, Jackson P, Savy P (2014). Bioorg Med Chem Lett.

[3] Jin M, Wang J, Kleinberg A, Kadalbajoo M, Siu K W, Cooke A, Bittner M A, Yao Y, Thelemann A, Ji O (2013). Bioorg Med Chem Lett.

[4] Crew A P, Bhagwat S V, Dong H, Bittner M A, Chan A, Chen X, Coate H, Cooke A, Gokhale P C, Honda A (2011). Bioorg Med Chem Lett.

[5] Mukaiyama H, Nishimura T, Kobayashi S, Ozawa T, Kamada N, Komatsu Y, Kikuchi S, Oonota H, Kusama H (2007). Bioorg Med Chem.

[6] Jin M, Kleinberg A, Cooke A, Gokhale P C, Foreman K, Dong H, Siu K W, Bittner M A, Mulvihill K M, Yao Y (2011). Bioorg Med Chem Lett.

[7] Kinzel O, Alfieri A, Altamura S, Brunetti M, Bufali S, Colaceci F, Ferrigno F, Filocamo G, Fonsi M, Gallinari P (2011). Bioorg Med Chem Lett.

[8] Wu J, Xing X, Cuny G D (2009). Lett Org Chem.

[9] Matsumoto H, Ikeda K, Nagata N, Takayanagi H, Mizuno Y, Tanaka M, Sasaki T (1999). J Med Chem.

[10] Roberts L R, Bradley P A, Bunnage M A, England K S, Fairman D, Fobian Y M, Fox D N A, Gymer G E, Heasley S E, Molette J (2011). Bioorg Med Chem Lett.

[11] Turtaut F, Lopez M, Ouahrani-Bettache S, Köhler S, Winum J-Y (2014). Bioorg Med Chem Lett.

[12] Livermore D G H, Bethell R C, Cammack N, Hancock A P, Harm M M, Green D V S, Lamont R B, Noble S A, Orr D C, Payne J J (1993). J Med Chem.

[13] Knight D J, Scopes D I C, Storer R, Holman S (1987). Imidazopyridazine derivatives having antiviral activity. U.S. Patent.

[14] Britt S D, Fu J, Parker D T, Patane M, Raman P, Radetich B, Seepersaud M, Yifru, A, Zheng R, Brand T (2010). Organic compounds and their uses. U.S. Pat. Appl..

[15] Chino A, Masuda N, Amano Y, Honbou K, Mihara T, Yamazaki M, Tomishima M (2014). Bioorg Med Chem.

[16] Heinelt U, Wehner V, Herrmann M, Schoenafinger K, Steinhagen H (2011). Imidazopyridazines as PAR1 inhibitors, production thereof, and use as medicaments. U.S. Pat. Appl..

[17] Wurz R P, Sastri C, D’Amico D C, Herberich B, Jackson C L M, Pettus L H, Tasker A S, Wu B, Guerrero N, Lipford J R (2016). Bioorg Med Chem Lett.

[18] Chen Y L (2001). Substituted 6,5-hetero-bicyclic derivatives. U.S. Pat. Appl..

[19] Saito T, Obitsu T, Kohno H, Sugimoto I, Matsushita T, Nishiyama T, Hirota T, Takeda H, Matsumura N, Ueno S (2012). Bioorg Med Chem.

[20] Brown R E, Burkamp F, Doughty V A, Fischer S R, Hollingworth G J, Jones A B, Sparey T J (2006). Substituted amino heterocycles as VR-1 antagonists for treating pain. U.S. Pat. Appl..

[21] Xu Y, Han B, Xie L, Maynard G D (2009). Imidazo-pyridazines, triazolo-pyridazines and related benzodiazepine receptor ligands. U.S. Pat. Appl..

[22] Wermuth C G (2011). Med Chem Commun.

[23] Knutsen L J S, Judkins B D, Mitchell W L, Newton R F, Scopes D I C (1984). J Chem Soc, Perkin Trans 1.

[24] Brückner R, Lavergne J-P, Viallefont P (1979). Liebigs Ann Chem.

[25] Golubushina G M, Poshtaruk G N, Chuiguk V A (1974). Chem Heterocycl Compd.

[26] Kutlescha K, Irrgang T, Kempe R (2010). New J Chem.

[27] Kolos N N, Orlov V D, Paponov B V, Shishkin O V (1999). Chem Heterocycl Compd.

[28] Kolos N N, Orlov V D, Paponov B V, Shishkin O V, Baumer V N (1998). Chem Heterocycl Compd.

[29] Lipson V V, Svetlichnaya N V, Shirobokov M G, Musatov V I, Shishkin O V, Shishkina S V (2012). Russ J Org Chem.

[30] Vandyshev D Y, Shikhaliev K S, Potapov A Y, Krysin M Y (2014). Chem Heterocycl Compd.

[31] Kolos N N, Beryozkina T V, Orlov V D (2002). Mendeleev Commun.

[32] Vandyshev D Y, Shikhaliev K S, Potapov A Y, Krysin M Y (2015). Chem Heterocycl Compd.

[33] Plaskon A S, Ryabukhin S V, Volochnyuk D M, Shivanyuk A N, Tolmachev A A (2008). Heterocycles.

[34] Lipson V V, Svetlichnaya N V, Shishkina S V, Shishkin O V (2008). Mendeleev Commun.

[35] Vandyshev D Y, Shikhaliev K S, Potapov A Y, Krysin M Y (2015). Chem Heterocycl Compd.

[36] Filimonov S I, Korsakov M K, Chirkova Z V, Abramov I G, Stashina G A, Firgang S I, Kovygin Y A, Shikhaliev K S (2013). Chem Heterocycl Compd.

[37] Medway A M, Sperry J (2014). Green Chem.

[38] Vandyshev D Y, Shikhaliev K S, Kokonova A V, Potapov A Y, Kolpakova M G, Sabynin A L, Zubkov F I (2016). Chem Heterocycl Compd.

[39] Chebanov V A, Gura K A, Desenko S M (2010). Top Heterocycl Chem.

[40] Rudenko R V, Komykhov S A, Desenko S M, Sen’ko Y V, Shishkin O V, Konovalova I S, Shishkina S V, Chebanov V A (2011). Synthesis.

[41] Havrylyuk D, Zimenkovsky B, Lesyk R (2009). Phosphorus, Sulfur Silicon Relat Elem.

[42] Lesyk R, Vladzimirska O, Holota S, Zaprutko L, Gzella A (2007). Eur J Med Chem.

[43] Taylor P J, Antonov L, Antonov L (2016). “Triage” for Tautomers: The Choice between Experiment and Computation. Tautomerism: Concepts and Applications in Science and Technology.

