# Supplementary material for: A novel synthetic approach to hydroimidazo[1,5-b]pyridazines by the recyclization of itaconimides and HPLC–HRMS monitoring of the reaction pathway
Source: Beilstein J Org Chem. 2017 Nov 30;13:2561–8. doi: 10.3762/bjoc.13.252 (PMC5727784; doi:10.3762/bjoc.13.252)
Supplement: File 1 — Experimental procedures, characterization data, copies of 1H, 13C spectra of the products and results of HPLC–HRESIMS monitoring of the reaction mixture composition. [file Beilstein_J_Org_Chem-13-2561-s001.pdf]

Supporting Information

for

A novel synthetic approach to hydroimidazo[1,5-*b*]pyridazines by the recyclization of itaconimides and HPLC–HRMS monitoring of the reaction pathway

Dmitry Yu. Vandyshev[1], Khidmet S. Shikhaliev[1], Andrey Yu. Potapov[1], Michael Yu. Krysin*[1], Fedor I. Zubkov[2], and Lyudmila V. Sapronova[2]

Address: [1]Faculty of Chemistry, Voronezh State University, Universitetskaya sq., 1, Voronezh 394018, Russian Federation and [2]Faculty of Physics and Mathematics and Natural Sciences, RUDN University, Miklukho-Maklaya St., 6, Moscow 117198, Russian Federation

Email: Michael Yu. Krysin - kaf261@rambler.ru

* Corresponding author

Experimental procedures, characterization data, copies of ^{1}H, ^{13}C spectra of the products and results of HPLC-HRESIMS monitoring of the reaction mixture composition

Table of contents

I. General information S2

II. General procedure for the reaction of itaconimides **1** with diaminoimidazole **4** and characterization data of imidazopyridazine **9** S3

III. References S6

IV. Copies of ^{1}H and ^{13}C spectra and the data of HPLC-HRMS-ESI analysis of the products **9** S7

V. Results of HPLC–HRESIMS monitoring of reaction mixture composition in the synthesis of imidazopyridazine **9d** S74

I. General information

^{1}H and ^{13}C NMR spectra were registered on Bruker DRX (500 and 125.8 MHz, respectively) spectrometer in DMSO-d_6 and TMS as internal standard. Mass spectra were registered on an Agilent Technologies LCMS 6230B (ESI). Melting points were determined on a Stuart SMP 30 apparatus. Qualitative control of reagents and products in reaction mass was performed by TLC using Merck TLC Silicagel 60 F_{254} chromatographic plates; eluents: methanol, chloroform and mixtures thereof in various ratios. The chromatograms were developed by UV and iodine vapor.

Monitoring of the reaction pathway and purity of the products was controlled by HPLC-ESIMS. The device consists of the liquid chromatograph Agilent 1269 Infinity and the time-of-flight high resolution mass detector Agilent 6230 TOF LC/MS. Block ionization is double electrospray, detection mass range is from 50 to 2 000 Dalton. Capillary voltage is 4.0 kV, fragmentor + 191 V, skimmer + 66 V, OctRF 750 V. Column Poroshell 120 EC-C18 (4.6 × 50 mm; 2.7 µm) was used. Gradient eluation: acetonitrile/water (0.1% formic acid); flow rate: 0,4 mL/min. Software for collection and elaboration of research results is MassHunter Workstation/Data Acquisition

V.06.00. Starting arylitaconimides **1** and diaminoimidazole **4** were obtained according to the procedure described previously [1,2].

II. General procedure for the reaction of itaconimides 1 with diaminoimidazole 4 and characterization data of imidazopyridazine 9

The mixture of 0.87 g (5 mmol) diaminoimidazole **4**, the corresponding *N*-arylitaconimide **1a**–**g** (5 mmol), isopropyl alcohol (5 mL) and 1–2 drops of acetic acid was refluxed for 1–2 h. The precipitated sediment was filtered and crystallized from a mixture of iPrOH/DMF 2:1. The 7-amino-2-oxo-5-phenyl-1,2,3,4-tetrahydroimidazo[1,5-*b*]pyridazin-3-yl)-*N*-arylacetamides **9a**–**g** were obtained as light gray powders.

2-(7-Amino-2-oxo-5-phenyl-1,2,3,4-tetrahydroimidazo[1,5-*b*]pyridazine-3-yl)-*N*-(4-methylphenyl)acetamide (9a).

1.163 g; yield 62%; m.p. 220-223 ^{o}C; ^{1}H NMR, δ: 2.23 (3H, s, CH_3); 2.84-2.94 (3H, m, CH_2 + $CH_{pyridaz}$); 3.24-3.33 (2H, m, $CH_{2\ pyridaz}$); 5.70 (2H, s, NH_2); 7.07 (2H, d, *J* = 8.4 Hz, CH_{arom}); 7.15 (1H, t, *J* = 7.4 Hz, CH_{arom}); 7.33 (2H, t, *J* = 7.7 Hz, CH_{arom}); 7.45 (2H, d, *J* = 8.4 Hz, CH_{arom}); 7.51 (2H, d, *J* = 7.3 Hz, CH_{arom}); 10.01 (1H, s, N$\underline{H}$CO); 11.50 (1H, br. s, $NH_{pyridaz}$); ^{13}C NMR, δ: 20.5 (CH_3); 24.4 (CH_2); 35.8 (C-4); 36.0 (C-3); 114.5 (C-4a); 119.1, 125.4, 125.5, 128.4, 129.0, 131.8, 136.8 (C Ar); 142.4 (C-7); 169.2 (CO); 170.2 (C-2); HRMS, m/z ($[M+H]^+$), calcd for $C_{21}H_{21}N_5O_2+H^+$ 376.1769, found 376.1766.

2-(7-Amino-2-oxo-5-phenyl-1,2,3,4-tetrahydroimidazo[1,5-*b*]pyridazin-3-yl)-*N*-(4-ethylphenyl)acetamide (9b).

1.167 g; yield 60%; m.p 215- 217 °C; ^{1}H NMR, δ: 1.14 (3H, t, *J* = 7.6, CH_3); 2.51-2.56 (2H, m, CH_2); 2.84-2.93 (3H, m, CH_2 + $CH_{pyridaz}$); 3.24-3.33 (2H, m, $CH_{2\ pyridaz}$); 5.59 (2H, s, NH_2); 7.10 (2H, d, *J* = 8.5 Hz, CH_{arom}); 7.15 (1H, t, *J* = 7.3 Hz, CH_{arom}); 7.33 (2H, t, *J* = 7.6 Hz, CH_{arom}); 7.46 (2H, d, *J* = 8.5 Hz, CH_{arom}); 7.51 (2H, d, *J* = 7.3 Hz, CH_{arom}); 9.95 (1H, s, N<u>H</u>CO); 11.31 (1H, br. s, $NH_{pyridaz}$); ^{13}C NMR, δ: 15.8 (CH_3); 24.5 (CH_2); 27.6 (<u>C</u>H_2CH_3); 35.6 (C-4); 36.0 (C-3); 114.4 (C-4a); 119.1, 125.2, 125.5, 127.9, 128.4, 137.0, 138.4 (C Ar); 142.4 (C-7); 169.1 (CO); 170.2 (C-2); HRMS, m/z ($[M+H]^+$), calcd for $C_{22}H_{23}N_5O_2$+H^+ 390.1926, found 390.1924.

2-(7-Amino-2-oxo-5-phenyl-1,2,3,4-tetrahydroimidazo[1,5-*b*]pyridazin-3-yl)-*N*-(3-chlorophenyl)acetamide (9c).

1.383 g; yield 70%; m.p 225- 227 °C; ^{1}H NMR, δ: 2.85-2.95 (3H, m, CH_2 + $CH_{pyridaz}$); 3.26-3.33 (2H, m, $CH_{2\ pyridaz}$); 5.64 (2H, s, NH_2); 7.08 (1H, dd, *J* = 7.9 Hz, *J* = 2.0 Hz, CH_{arom}); 7.16 (1H, t, *J* = 7.4 Hz, CH_{arom}); 7.29-7.36 (3H, m, CH_{arom}); 7.41 (1H, dd, *J* = 8.3 Hz, *J* = 2.0 Hz, CH_{arom}); 7.52 (2H, d, *J* = 7.3 Hz, CH_{arom}); 7.78 (1H, t, *J* = 2.0 Hz, CH_{arom}); 10.24 (1H, s, N<u>H</u>CO); 11.34 (1H, br. s, $NH_{pyridaz}$); ^{13}C NMR, δ: 24.4 (CH_2); 35.8 (C-4); 35.9 (C-3); 114.5 (C-4a); 117.4, 118.5, 122.7, 125.2, 125.6, 128.4, 130.4, 133.1, 140.7 (C Ar); 142.3 (C-7); 169.9 (CO); 170.0 (C-2); HRMS, m/z ($[M+H]^+$), calcd for $C_{20}H_{18}ClN_5O_2$+H^+ 396.1223, found 396.1220.

2-(7-Amino-2-oxo-5-phenyl-1,2,3,4-tetrahydroimidazo[1,5-*b*]pyridazin-3-yl)-*N*-(4-chlorophenyl)acetamide (9d).

1.422 g; yield 72%; m.p 245- 247 °C; ^{1}H NMR, δ: 2.85-2.94 (3H, m, CH_2 + $CH_{pyridaz}$); 3.25-3.33 (2H, m, $CH_{2\ pyridaz}$); 5.63 (2H, s, NH_2); 7.16 (1H, t, *J* = 7.3 Hz, CH_{arom}); 7.31-7.36 (4H, m, CH_{arom}); 7.51 (2H, d, *J* = 7.7 Hz, CH_{arom}); 7.60 (2H, d, *J* = 8.8 Hz, CH_{arom}); 10.19 (1H, s, N<u>H</u>CO); 11.35 (1H, br. s, $NH_{pyridaz}$); ^{13}C NMR, δ: 24.4 (CH_2); 35.7 (C-4); 36.0 (C-3); 114.4 (C-4a); 120.6, 125.2, 125.5, 126.5, 126.7, 128.4, 128.6, 134.7, 138.2 (C Ar); 142.4 (C-7); 169.6 (CO); 170.2 (C-2); HRMS, m/z ($[M+H]^+$), calcd for $C_{20}H_{18}ClN_5O_2+H^+$ 396.1223, found 396.1225.

2-(7-Amino-2-oxo-5-phenyl-1,2,3,4-tetrahydroimidazo[1,5-*b*]pyridazin-3-yl)-*N*-(3,4-dimethylphenyl)acetamide (9e).

1.264 g; yield 65%; m.p 256- 259 °C; ^{1}H NMR, δ: 2.14 (3H, s, CH_3); 2.16 (3H, s, CH_3); 2.83-2.94 (3H, m, CH_2 + $CH_{pyridaz}$); 3.24-3.33 (2H, m, $CH_{2\ pyridaz}$); 5.63 (2H, s, NH_2); 7.01 (1H, d, *J* = 8.2 Hz, CH_{arom}); 7.15 (1H, t, *J* = 7.4 Hz, CH_{arom}); 7.26 (1H, dd, *J* = 8.2 Hz, *J* = 2.0 Hz, CH_{arom}); 7.31-7.35 (3H, m, CH_{arom}); 7.51 (2H, d, *J* = 7.4 Hz, CH_{arom}); 9.87 (1H, sc, N<u>H</u>CO); 11.34 (1H, br. s, $NH_{pyridaz}$); ^{13}C NMR, δ: 18.8, 19.7 (CH_3); 24.4 (CH_2); 35.0 (C-4); 36.0 (C-3); 114.4 (C-4a); 116.6, 120.3, 125.2, 125.5, 128.4, 129.5, 130.7, 136.2, 137.0 (C Ar); 142.4 (C-7); 169.0 (CO); 170.3 (C-2); HRMS, m/z ($[M+H]^+$), calcd for $C_{22}H_{23}N_5O_2+H^+$ 390.1926, found 390.1900.

2-(7-Amino-2-oxo-5-phenyl-1,2,3,4-tetrahydro-imidazo[1,5-*b*]pyridazin-3-yl)-*N*-(3,5-dimethylphenyl)acetamide (9f).

1.303 g; yield 67%; m.p 235-237 °C; ^{1}H NMR, δ: 2.21 (6H, s, CH_3); 2.83-2.94 (3H, m, CH_2 + $CH_{pyridaz}$); 3.24-3.33 (2H, m, $CH_{2pyridaz}$); 5.62 (2H, s, NH_2); 6.66 (1H, s, CH_{arom}); 7.15 (1H, t, *J* = 7.4 Hz, CH_{arom}); 7.18 (2H, s, CH_{arom}); 7.33 (2H, t, *J* = 7.7 Hz, CH_{arom});

7.51 (2H, d, *J* = 7.7 Hz, CH_{arom}); 9.87 (1H, s, N<u>H</u>CO); 11.30 (1H, br. s, $NH_{pyridaz}$); ^{13}C NMR, δ: 21.0 ($2CH_3$); 24.3 (CH_2); 35.6 (C-4); 35.9 (C-3); 114.2 (C-4a); 116.7, 124.5, 125.1, 125.4, 128.3, 137.5, 139.0 (C Ar); 142.2 (C-7); 169.1 (CO); 170.1 (C-2); HRMS, m/z ($[M+H]^+$), calcd for $C_{22}H_{23}N_5O_2+H^+$ 390.1926, found 390.1904.

2-(7-Amino-2-oxo-5-phenyl-1,2,3,4-tetrahydroimidazo[1,5-*b*]pyridazin-3-yl)-*N*-(3,4-dichlorophenyl)acetamide (9g).

1.416 g; yield 66%; m.p 237-239 ^{o}C; ^{1}H NMR, δ: 2.85-2.96 (3H, m, CH_2 + $CH_{pyridaz}$); 3.26-3.33 (2H, m, $CH_{2\ pyridaz}$); 5.64 (2H, s, NH_2); 7.16 (1H, t, *J* = 7.5 Hz, CH_{arom}); 7.34 (2H, t, *J* = 7.8 Hz, CH_{arom}); 7.45 (1H, dd, *J* = 8.9 Hz, *J* = 2.4 Hz, CH_{arom}); 7.50-7.55 (3H, m, CH_{arom}); 7.96 (1H, d, *J* = 2.4 Hz, CH_{arom}); 10.34 (1H, s, N<u>H</u>CO); 11.35 (1H, br. s, $NH_{pyridaz}$); ^{13}C NMR, δ: 24.4 (CH_2); 35.8 (C-4); 35.9 (C-3); 114.4 (C-4a); 119.1, 120.2, 124.4, 125.2, 125.5, 128.4, 130.7, 131.0, 139.3 (C Ar); 142.4 (C-7); 170.0 (CO); 170.1 (C-2); HRMS, m/z ($[M+H]^+$), calcd for $C_{20}H_{17}Cl_2N_5O_2+H^+$ 430.0833, found 430.0829.

III. References.

1. Abdel-Naby, A. S. *J. Appl. Polym. Sci.* **2011**, *121*, 169–175.
doi: 10.1002/app.33507

2. Ivashchenko, A. V.; Lazareva, V. T.; Prudnikova, E. K.; Ivashchenko, S. P.; Rumyantsev, V. G. *Chem. Heterocycl. Comp.* **1982**, *18*, 185-189.
doi: 10.1007/BF00512966

IV. Copies of ^{1}H, ^{13}C NMR spectra and the data of HPLC–HRESIMS analysis of the products 9.

2-(7-Amino-2-oxo-5-phenyl-1,2,3,4-tetrahydroimidazo[1,5-*b*]pyridazine-3-yl)-*N*-(4-methylphenyl)acetamide (9a)

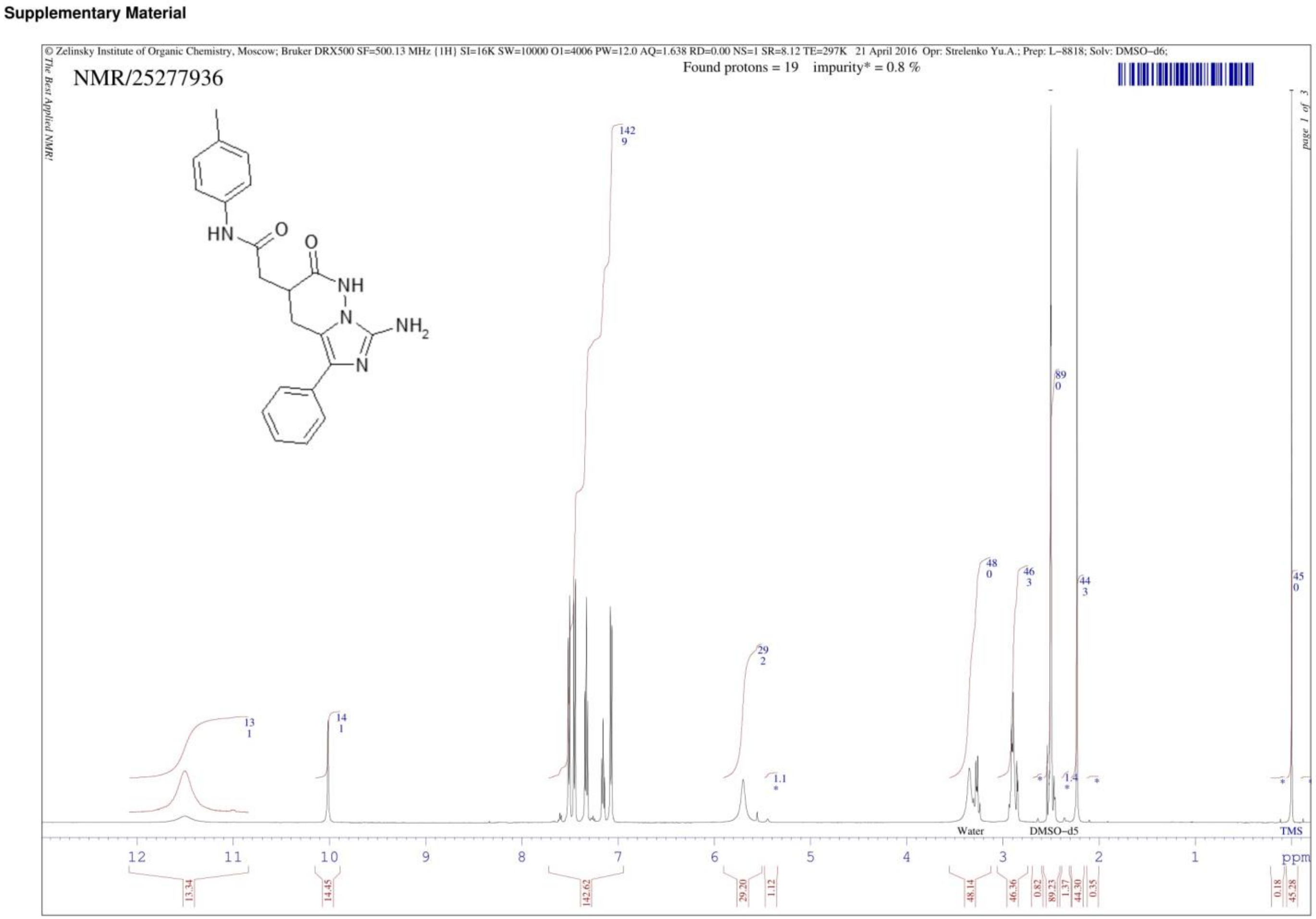

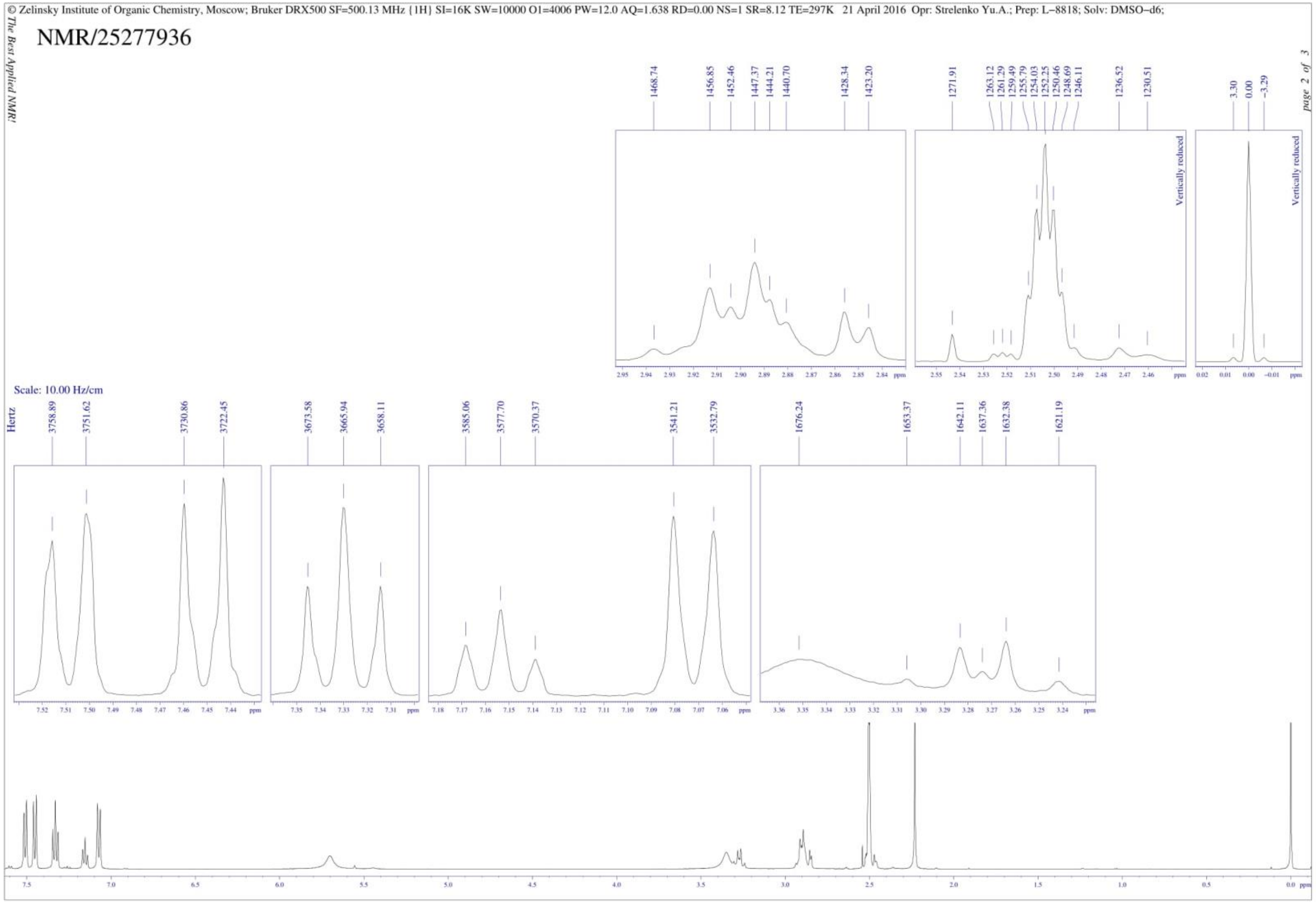
© Zelinsky Institute of Organic Chemistry, Moscow; Bruker DRX500 SF=500.13 MHz {1H} SI=16K SW=10000 O1=4006 PW=12.0 AQ=1.638 RD=0.00 NS=1 SR=8.12 TE=297K 21 April 2016 Opr: Strelenko Yu.A.; Prep: L−8818; Solv: DMSO−d6;
The Best Applied NMR!
NMR/25277936
page 2 of 3
Scale: 10.00 Hz/cm
Hertz
Vertically reduced
Vertically reduced
ppm

© Zelinsky Institute of Organic Chemistry, Moscow; Bruker DRX500 SF=500.13 MHz {1H} SI=16K SW=10000 O1=4006 PW=12.0 AQ=1.638 RD=0.00 NS=1 SR=8.12 TE=297K 21 April 2016 Opr: Strelenko Yu.A.; Prep: L–8818; Solv: DMSO–d6;

The Best Applied NMR!

NMR/25277936

Peaks List

#	Address [points]	Frequency [Hz]	Frequency [ppm]	Intensity [cm]
1	13072.3	5008.465	10.0143	1.70
2	17166.9	3758.891	7.5158	3.31
3	17190.7	3751.617	7.5013	3.89
4	17258.7	3730.858	7.4598	4.11
5	17286.3	3722.452	7.4430	4.67
6	17446.4	3673.578	7.3452	2.37
7	17471.5	3665.938	7.3300	4.05
8	17497.1	3658.115	7.3143	2.35
9	17736.5	3585.062	7.1683	1.10
10	17760.6	3577.705	7.1536	1.86
11	17784.6	3570.369	7.1389	0.80
12	17880.2	3541.207	7.0806	3.83
13	17907.8	3532.787	7.0637	3.53
14	20136.8	2852.543	5.7036	0.63
15	23991.3	1676.238	3.3516	0.80
16	24066.3	1653.365	3.3059	0.38
17	24103.1	1642.114	3.2834	1.05
18	24118.7	1637.355	3.2739	0.54
19	24135.0	1632.377	3.2639	1.18
20	24171.7	1621.195	3.2415	0.34
21	24671.2	1468.737	2.9367	0.27
22	24710.2	1456.847	2.9129	1.57
23	24724.6	1452.462	2.9042	1.16
24	24741.3	1447.369	2.8940	2.11
25	24751.6	1444.207	2.8877	1.32
26	24763.1	1440.698	2.8806	0.84
27	24803.6	1428.336	2.8559	1.06
28	24820.5	1423.201	2.8457	0.73
29	25316.2	1271.915	2.5432	1.58
30	25345.0	1263.116	2.5256	0.46
31	25351.0	1261.286	2.5219	0.54
32	25356.9	1259.488	2.5183	0.45
33	25369.0	1255.788	2.5109	3.83
34	25374.8	1254.031	2.5074	8.80
35	25380.6	1252.247	2.5038	12.62
36	25386.5	1250.457	2.5003	8.88
37	25392.3	1248.687	2.4967	4.01
38	25400.7	1246.112	2.4916	0.81
39	25432.2	1236.518	2.4724	0.80
40	25451.9	1230.510	2.4604	0.40
41	25827.0	1116.036	2.2315	12.57
42	29473.2	3.298	0.0066	0.62
43	29484.0	0.002	0.0000	30.93
44	29494.8	-3.295	-0.0066	0.62

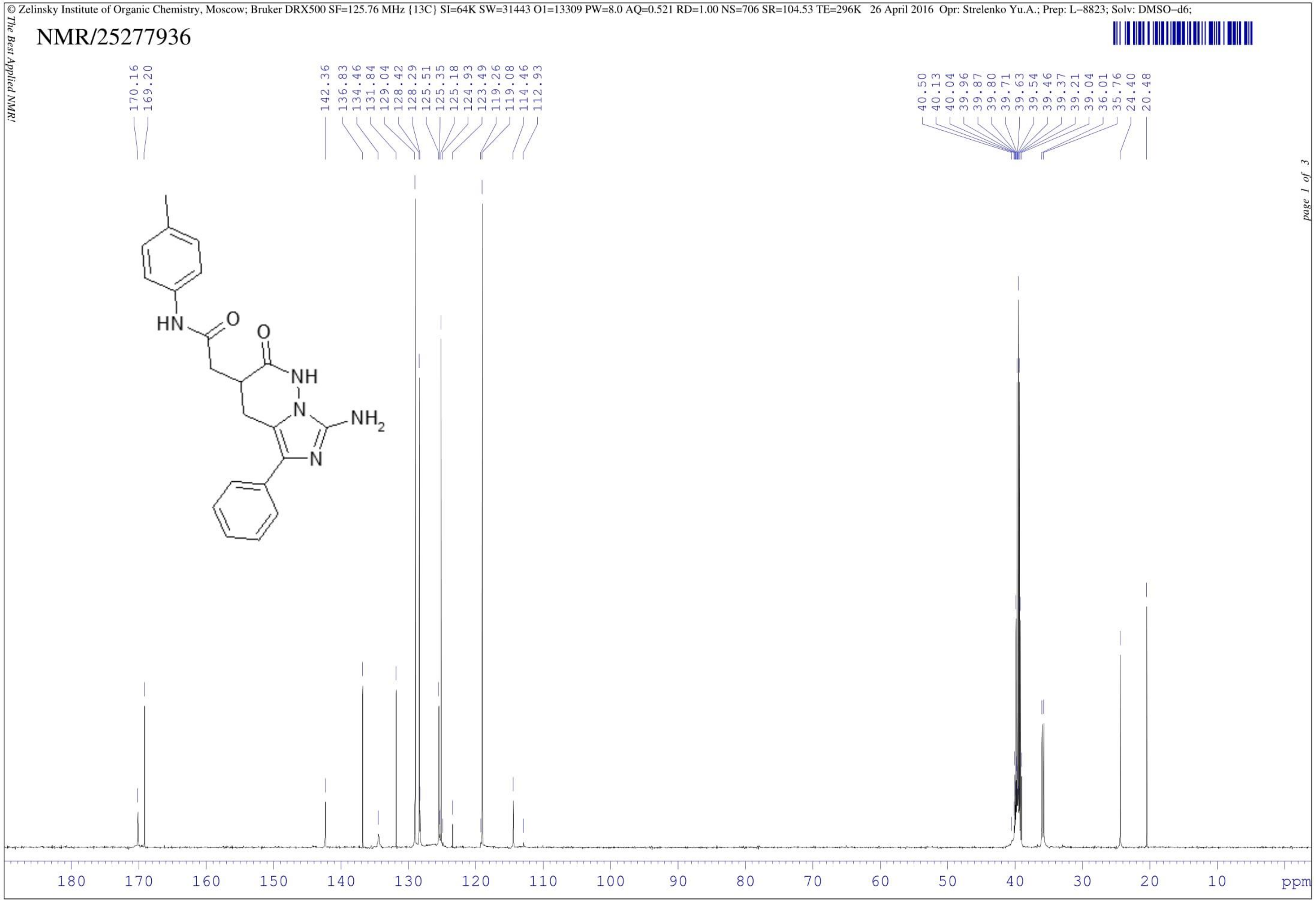
© Zelinsky Institute of Organic Chemistry, Moscow; Bruker DRX500 SF=125.76 MHz {13C} SI=64K SW=31443 O1=13309 PW=8.0 AQ=0.521 RD=1.00 NS=706 SR=104.53 TE=296K 26 April 2016 Opr: Strelenko Yu.A.; Prep: L–8823; Solv: DMSO–d6;
The Best Applied NMR!
NMR/25277936
170.16
169.20
142.36
136.83
134.46
131.84
129.04
128.42
128.29
125.51
125.35
125.18
124.93
123.49
119.26
119.08
114.46
112.93
40.50
40.13
40.04
39.96
39.87
39.80
39.71
39.63
39.54
39.46
39.37
39.21
39.04
36.01
35.76
24.40
20.48
page 1 of 3
HN
O
O
NH
N
NH_2
N
180
170
160
150
140
130
120
110
100
90
80
70
60
50
40
30
20
10
ppm

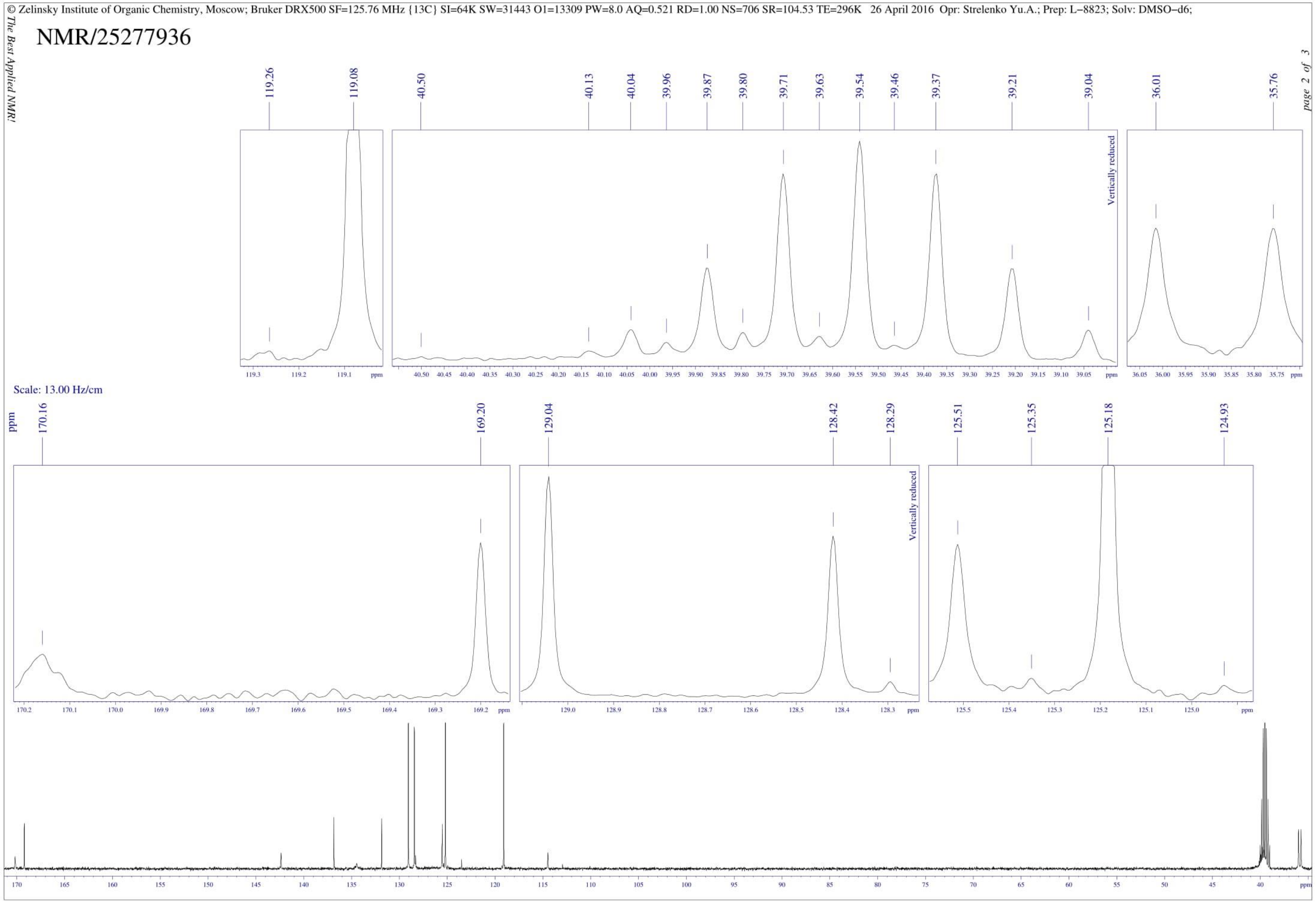
© Zelinsky Institute of Organic Chemistry, Moscow; Bruker DRX500 SF=125.76 MHz {13C} SI=64K SW=31443 O1=13309 PW=8.0 AQ=0.521 RD=1.00 NS=706 SR=104.53 TE=296K 26 April 2016 Opr: Strelenko Yu.A.; Prep: L−8823; Solv: DMSO−d6;
The Best Applied NMR!
NMR/25277936
page 2 of 3
119.26
119.08
40.50
40.13
40.04
39.96
39.87
39.80
39.71
39.63
39.54
39.46
39.37
39.21
39.04
36.01
35.76
Vertically reduced
Scale: 13.00 Hz/cm
ppm
170.16
169.20
129.04
128.42
128.29
125.51
125.35
125.18
124.93
Vertically reduced
ppm

© Zelinsky Institute of Organic Chemistry, Moscow; Bruker DRX500 SF=125.76 MHz {13C} SI=64K SW=31443 O1=13309 PW=8.0 AQ=0.521 RD=1.00 NS=706 SR=104.53 TE=296K 26 April 2016 Opr: Strelenko Yu.A.; Prep: L−8823; Solv: DMSO−d6;

The Best Applied NMR!

NMR/25277936

Peaks List

#	Address [points]	Frequency [Hz]	Frequency [ppm]	Intensity [cm]
1	15690.7	21398.906	170.1596	0.90
2	15942.2	21278.219	169.1999	3.27
3	22975.4	17903.443	142.3644	1.13
4	24425.9	17207.402	136.8297	3.69
5	25045.9	16909.902	134.4640	0.40
6	25733.8	16579.842	131.8394	3.61
7	26466.9	16228.079	129.0423	14.00
8	26630.2	16149.719	128.4192	10.22
9	26662.9	16134.012	128.2943	0.96
10	27392.0	15784.170	125.5124	3.23
11	27434.4	15763.843	125.3508	0.40
12	27478.5	15742.665	125.1824	11.14
13	27544.9	15710.789	124.9289	0.25
14	27922.5	15529.638	123.4884	0.69
15	29029.5	14998.434	119.2644	0.22
16	29077.9	14975.214	119.0798	13.89
17	30287.9	14394.621	114.4630	1.17
18	30689.3	14202.001	112.9313	0.33
19	49672.3	5093.299	40.5008	0.26
20	49768.5	5047.129	40.1337	0.57
21	49792.7	5035.514	40.0414	1.72
22	49813.0	5025.764	39.9638	1.03
23	49836.5	5014.515	39.8744	5.07
24	49856.9	5004.703	39.7963	1.57
25	49880.1	4993.567	39.7078	10.10
26	49900.9	4983.611	39.6286	1.36
27	49923.9	4972.550	39.5407	11.85
28	49943.9	4962.985	39.4646	0.87
29	49967.7	4951.556	39.3737	10.14
30	50011.4	4930.553	39.2067	5.03
31	50055.2	4909.566	39.0398	1.70
32	50848.1	4529.109	36.0145	2.82
33	50915.4	4496.804	35.7576	2.82
34	53892.9	3068.069	24.3966	4.35
35	54920.6	2574.972	20.4756	5.42

**Data File**	LCMS_2679.d	**Sample Name**	#1539
**Sample Type**	Sample	**Position**	Vial 3
**Instrument Name**	Instrument 1	**User Name**	Falaleev A.
**Acq Method**	ACN-H2O_30-70.m	**Acquired Time**	22-Dec-16 6:58:46 PM
**IRM Calibration Status**	Success	**DA Method**	13032017.m
**Comment**	4a / 2-(7-amino-2-oxo-5-phenyl-1,2,3,4-tetrahydroimidazo[1,5-b]pyridazin-3-yl)-N-(p-tolyl)acetamide		

**Stream Name**	LC 1	**Acquisition SW Version**	6200 series TOF/6500 series Q-TOF B.06.01 (B6157)

**Fragmentor Voltage**	191	**Collision Energy**	0	**Ionization Mode**	ESI

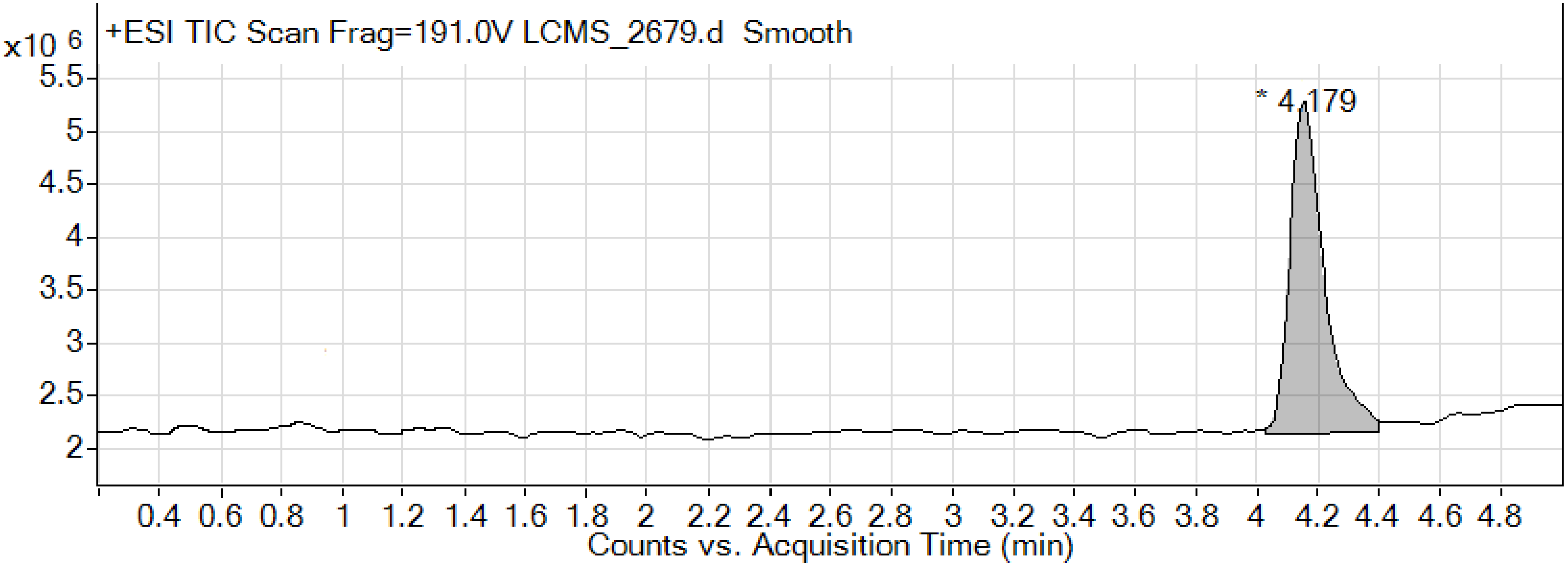

User Chromatogram Peak List

RT	Height	Height %	Area	Area %	Area Sum %	S/N	Symmetry	Width
4,179	273184,54	100	1810459,47	100	100	1421,8	1,2	0,291

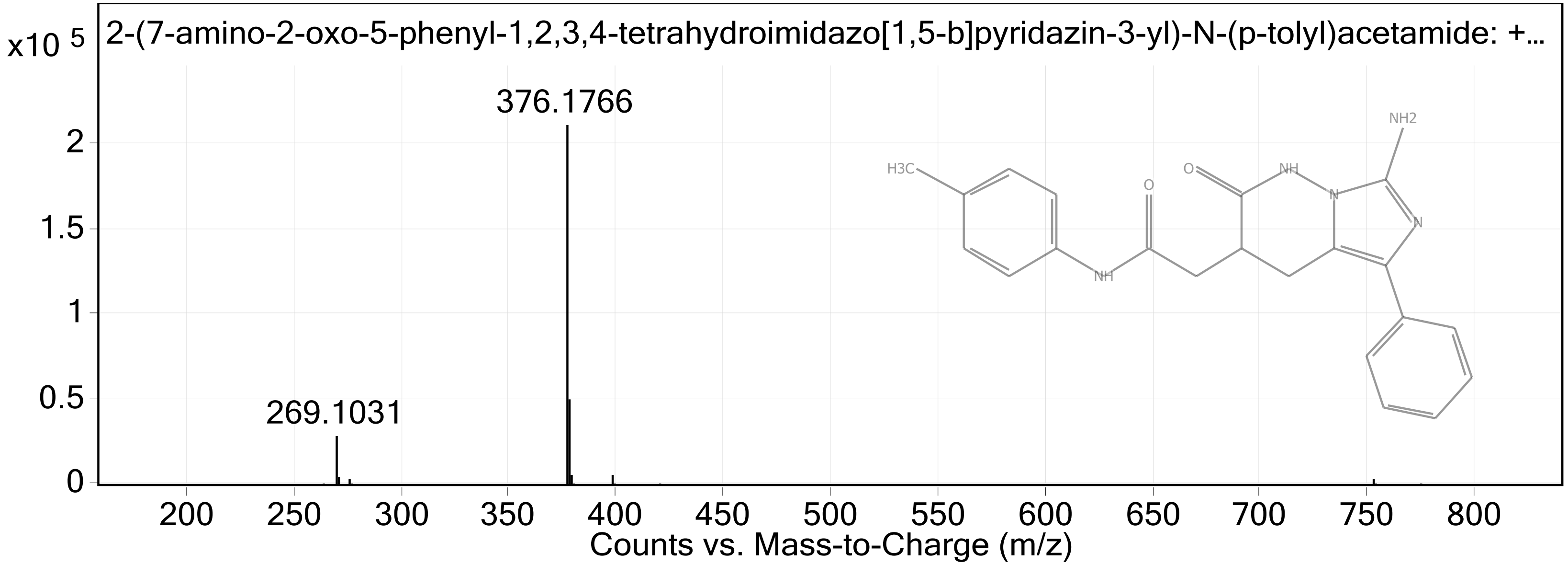

MS Spectrum Peak List

m/z	z	Abund
269,1031	1	28663,44
270,1062	1	4725,35
376,1766	1	211900,03
377,1795	1	50675,81
378,182	1	6966,59
398,1579		6309,32

Compound Structure

--- End Of Report ---

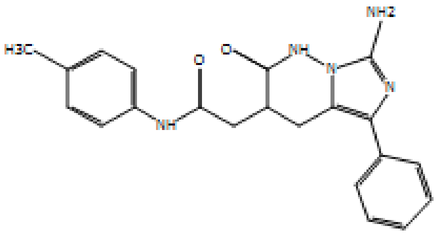

2-(7-Amino-2-oxo-5-phenyl-1,2,3,4-tetrahydroimidazo[1,5-*b*]pyridazin-3-yl)-*N*-(4-ethylphenyl)acetamide (9b)

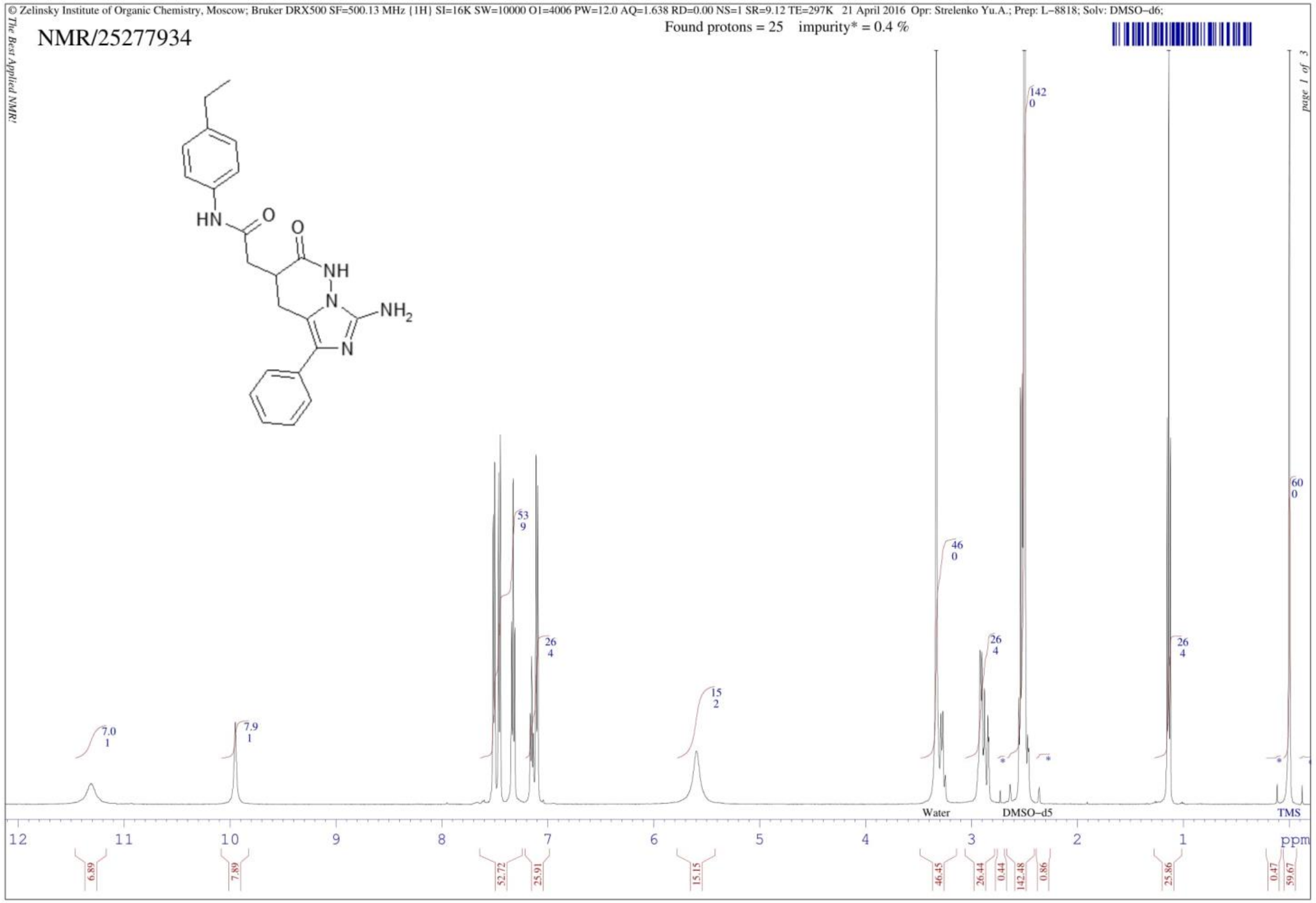

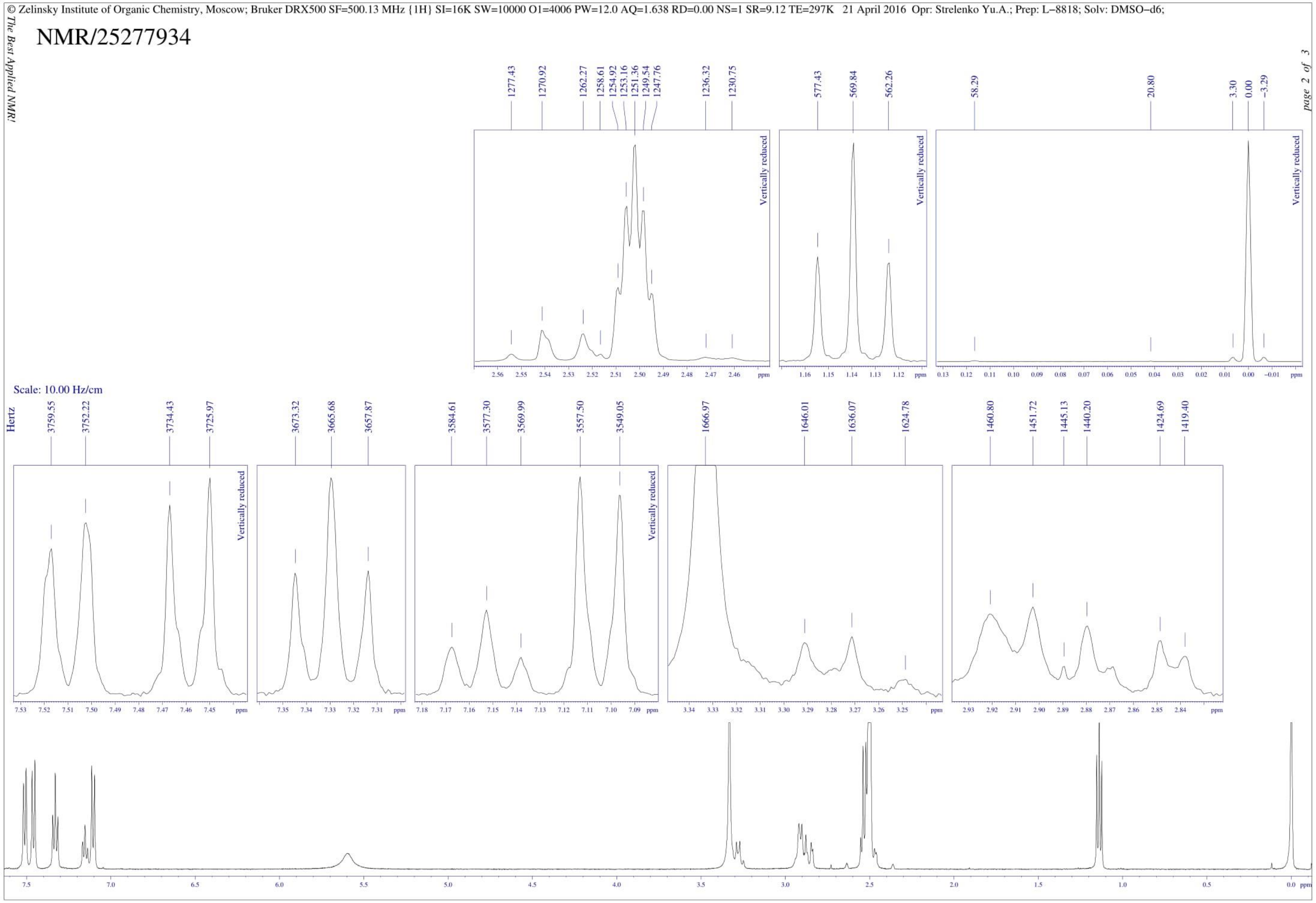
© Zelinsky Institute of Organic Chemistry, Moscow; Bruker DRX500 SF=500.13 MHz {1H} SI=16K SW=10000 O1=4006 PW=12.0 AQ=1.638 RD=0.00 NS=1 SR=9.12 TE=297K 21 April 2016 Opr: Strelenko Yu.A.; Prep: L–8818; Solv: DMSO–d6;
The Best Applied NMR!
NMR/25277934
page 2 of 3
Scale: 10.00 Hz/cm
Hertz
Vertically reduced
ppm

Peaks List

#	Address [points]	Frequency [Hz]	Frequency [ppm]	Intensity [cm]
1	10950.8	5654.678	11.3064	0.25
2	13168.1	4977.983	9.9534	0.95
3	17160.7	3759.548	7.5171	4.02
4	17184.7	3752.221	7.5025	4.71
5	17243.0	3734.434	7.4669	5.18
6	17270.8	3725.969	7.4500	5.95
7	17443.3	3673.315	7.3447	2.64
8	17468.3	3665.678	7.3295	4.65
9	17493.9	3657.867	7.3138	2.67
10	17734.0	3584.609	7.1674	1.19
11	17757.9	3577.300	7.1527	2.06
12	17781.9	3569.989	7.1381	0.94
13	17822.8	3557.496	7.1131	5.23
14	17850.5	3549.050	7.0963	4.85
15	20315.9	2796.659	5.5919	0.58
16	24017.7	1666.967	3.3331	10.35
17	24086.4	1646.011	3.2912	1.15
18	24118.9	1636.069	3.2713	1.27
19	24155.9	1624.781	3.2487	0.38
20	24693.3	1460.797	2.9208	1.76
21	24723.0	1451.722	2.9027	1.90
22	24744.6	1445.128	2.8895	0.66
23	24760.8	1440.200	2.8797	1.51
24	24811.6	1424.690	2.8486	1.20
25	24828.9	1419.402	2.8381	0.86
26	25004.7	1365.762	2.7308	0.26
27	25158.7	1318.768	2.6368	0.31
28	25294.1	1277.434	2.5542	1.46
29	25315.5	1270.918	2.5412	6.28
30	25343.8	1262.274	2.5239	5.49
31	25355.8	1258.606	2.5166	1.45
32	25367.9	1254.922	2.5092	14.51
33	25373.6	1253.163	2.5057	30.68
34	25379.6	1251.356	2.5021	43.13
35	25385.5	1249.544	2.4984	29.98
36	25391.4	1247.755	2.4949	13.39
37	25428.8	1236.324	2.4720	0.82
38	25447.1	1230.746	2.4609	0.69
39	25607.0	1181.936	2.3633	0.28
40	27587.9	577.427	1.1546	6.84
41	27612.8	569.836	1.1394	14.37
42	27637.6	562.264	1.1242	6.60
43	29289.0	58.291	0.1166	0.43
44	29411.8	20.803	0.0416	0.23
45	29469.2	3.297	0.0066	1.85
46	29480.0	0.001	0.0000	92.23
47	29490.8	-3.295	-0.0066	1.85
48	29676.2	-59.885	-0.1197	0.39

NMR/25277934

© Zelinsky Institute of Organic Chemistry, Moscow; Bruker DRX500 SF=500.13 MHz {} SI=16K SW=10000 O1=4006 PW=12.0 AQ=1.638 RD=0.00 NS=0 SR=9.12 TE=297K 21 April 2016 Opr: Strelenko Yu.A.; Prep: L-8818; Solv: DMSO-d6;

© The Best Applied NMR!

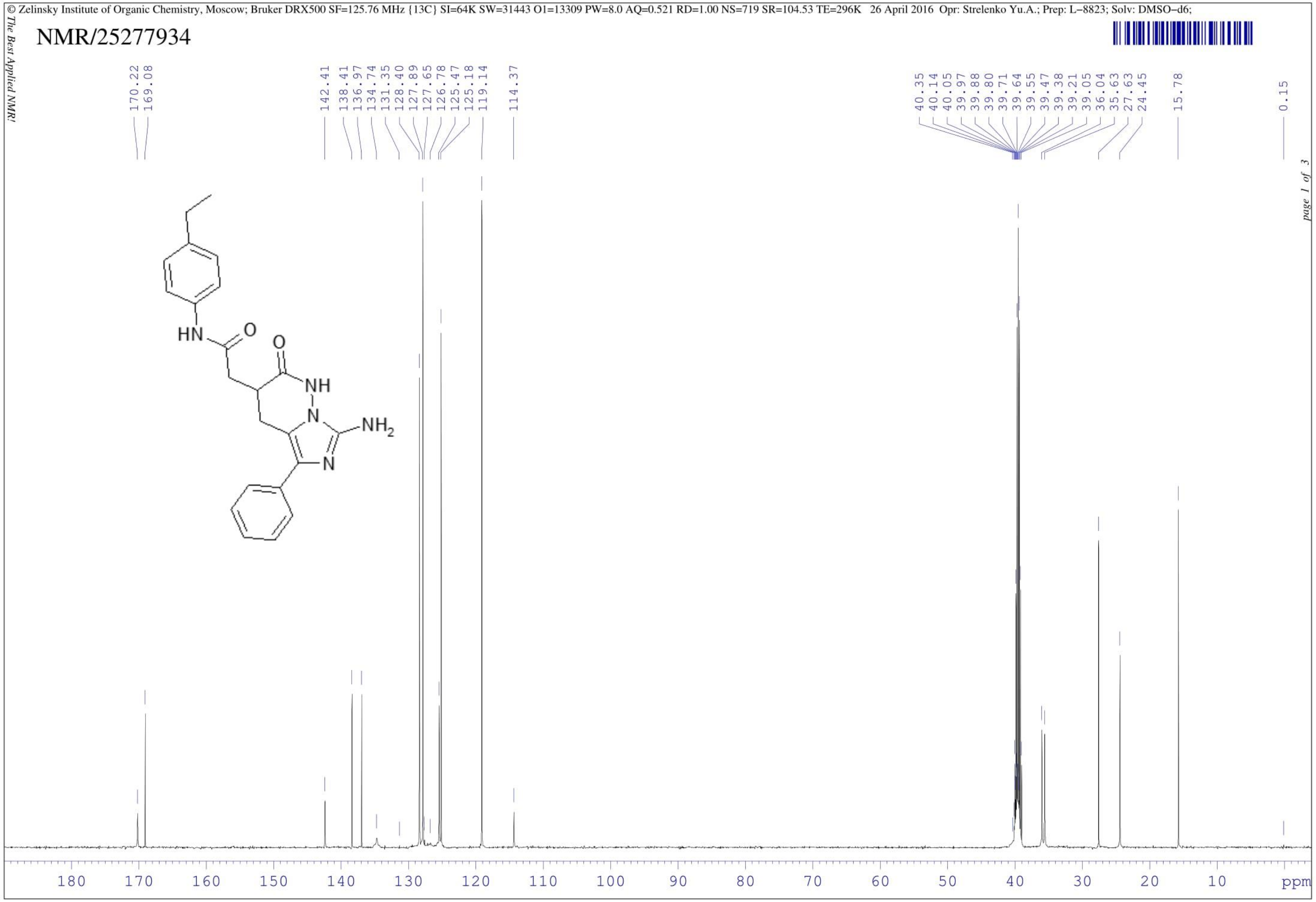

© Zelinsky Institute of Organic Chemistry, Moscow; Bruker DRX500 SF=125.76 MHz {13C} SI=64K SW=31443 O1=13309 PW=8.0 AQ=0.521 RD=1.00 NS=719 SR=104.53 TE=296K 26 April 2016 Opr: Strelenko Yu.A.; Prep: L–8823; Solv: DMSO–d6;
The Best Applied NMR!
NMR/25277934
170.22
169.08
142.41
138.41
136.97
134.74
131.35
128.40
127.89
127.65
126.78
125.47
125.18
119.14
114.37
40.35
40.14
40.05
39.97
39.88
39.80
39.71
39.64
39.55
39.47
39.38
39.21
39.05
36.04
35.63
27.63
24.45
15.78
0.15
page 1 of 3
HN
O
O
NH
N
NH2
N
180
170
160
150
140
130
120
110
100
90
80
70
60
50
40
30
20
10
ppm

© Zelinsky Institute of Organic Chemistry, Moscow; Bruker DRX500 SF=125.76 MHz {13C} SI=64K SW=31443 O1=13309 PW=8.0 AQ=0.521 RD=1.00 NS=719 SR=104.53 TE=296K 26 April 2016 Opr: Strelenko Yu.A.; Prep: L–8823; Solv: DMSO–d6;

NMR/25277934

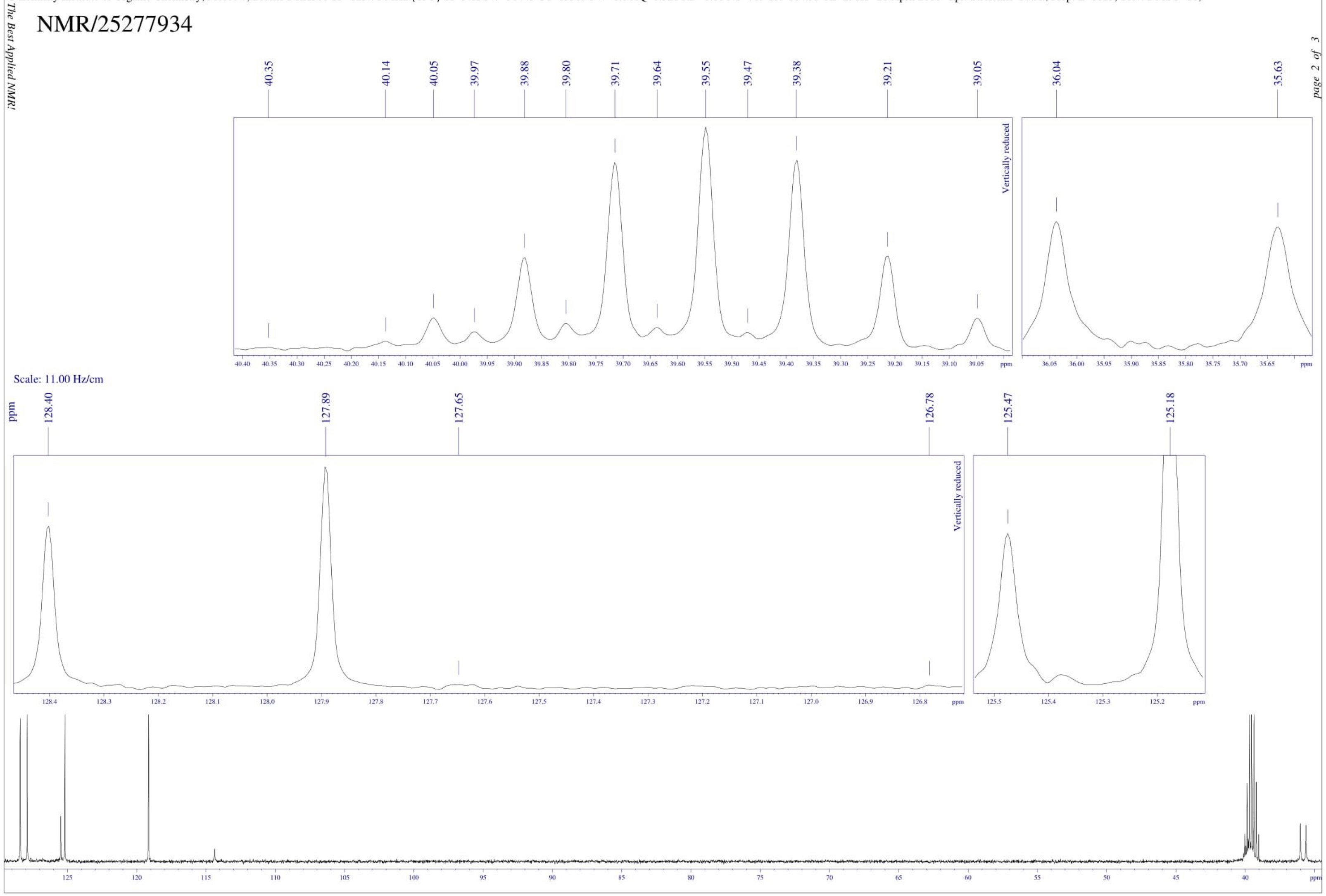

© Zelinsky Institute of Organic Chemistry, Moscow; Bruker DRX500 SF=500.13 MHz {1H} SI=16K SW=10000 O1=4006 PW=12.0 AQ=1.638 RD=0.00 NS=1 SR=8.67 TE=299K 15 March 2016 Opr: Strelenko Yu.A.; Prep: L–8797; Solv: DMSO–d6;

The Best Applied NMR!

NMR/25277683

Peaks List

#	Address [points]	Frequency [Hz]	[ppm]	Intensity [cm]
1	12699.4	5121.342	10.2400	2.53
2	16716.1	3895.542	7.7891	3.87
3	16722.7	3893.528	7.7850	6.85
4	16729.5	3891.456	7.7809	3.68
5	17147.2	3763.982	7.5260	7.14
6	17171.2	3756.669	7.5114	8.03
7	17319.9	3711.274	7.4206	2.55
8	17347.1	3702.976	7.4040	3.35
9	17434.8	3676.219	7.3505	5.43
10	17459.5	3668.676	7.3354	9.02
11	17477.1	3663.314	7.3247	5.35
12	17485.3	3660.801	7.3197	5.35
13	17503.2	3655.334	7.3088	8.12
14	17529.7	3647.235	7.2926	4.02
15	17723.6	3588.070	7.1743	2.39
16	17748.0	3580.619	7.1594	4.08
17	17772.3	3573.222	7.1446	1.74
18	17865.2	3544.865	7.0879	3.12
19	17891.3	3536.909	7.0720	2.76
20	20237.5	2820.889	5.6403	1.37
21	24025.2	1664.990	3.3291	2.30
22	24061.4	1653.941	3.3070	3.24
23	24093.9	1644.008	3.2872	3.12
24	24130.9	1632.723	3.2646	1.13
25	24665.8	1469.473	2.9382	3.48
26	24681.2	1464.782	2.9288	2.83
27	24698.0	1459.667	2.9186	4.50
28	24735.1	1448.320	2.8959	2.83
29	24785.5	1432.957	2.8652	2.45
30	24803.7	1427.388	2.8540	1.81
31	25297.6	1276.663	2.5527	1.56
32	25314.7	1271.465	2.5423	5.02
33	25349.0	1260.992	2.5213	2.52
34	25366.9	1255.522	2.5104	6.95
35	25373.0	1253.652	2.5067	13.77
36	25379.0	1251.837	2.5030	19.31
37	25385.0	1250.010	2.4994	13.37
38	25391.0	1248.174	2.4957	5.80
39	26072.0	1040.331	2.0801	0.88
40	26077.9	1038.557	2.0766	0.81
41	26348.9	955.839	1.9112	1.13
42	29470.2	3.297	0.0066	0.68
43	29481.0	0.001	0.0000	34.26
44	29491.8	-3.294	-0.0066	0.68

**Data Filename**	LCMS_2681.d	**Sample Name**	#1540
**Sample Type**	Sample	**Position**	Vial 4
**Instrument Name**	Instrument 1	**User Name**	Falaleev A.
**Acq Method**	ACN-H2O_40-60.m	**Acquired Time**	22-Dec-16 7:2
**IRM Calibration Status**	Success	**DA Method**	alex20150126
**Comment**	4b / 2-(7-amino-2-oxo-5-phenyl-1,2,3,4-tetrahydroimidazo[1,5-b]pyridazin-3-yl)-N-(4-ethylphenyl)acetamide		

**Stream Name**	LC 1	**Acquisition SW Version**	6200 series TOF/6500 series Q-TOF B.06.01 (B6157)

User Chromatograms

**Fragmentor Voltage**	191	**Collision Energy**	0	**Ionization Mode**	ESI

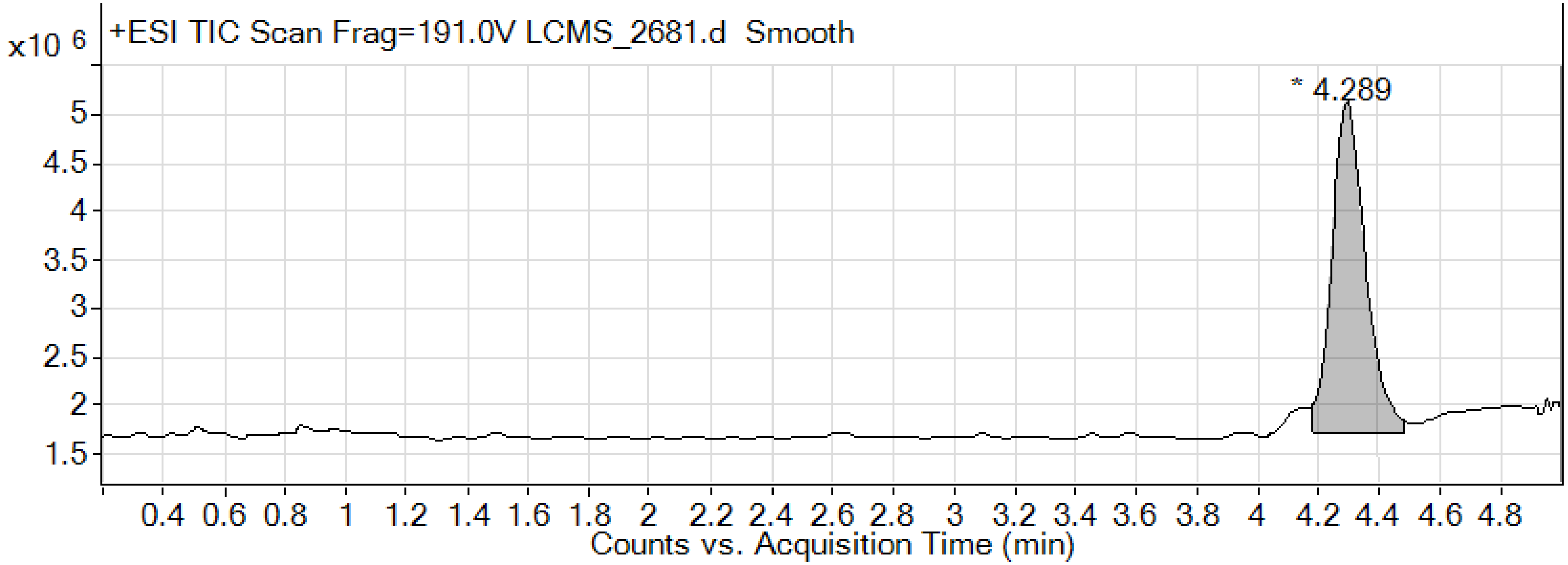

Integration Peak List

Peak	Start	RT	End	Height	Area	Area %
1	4,089	4,289	4,511	3434764,48	26390977,78	100

User Spectra

Spectrum Source	Fragmentor Voltage	Collision Energy	Ionization Mode
Peak (1) in "+ TIC Scan Smo"	191	0	ESI

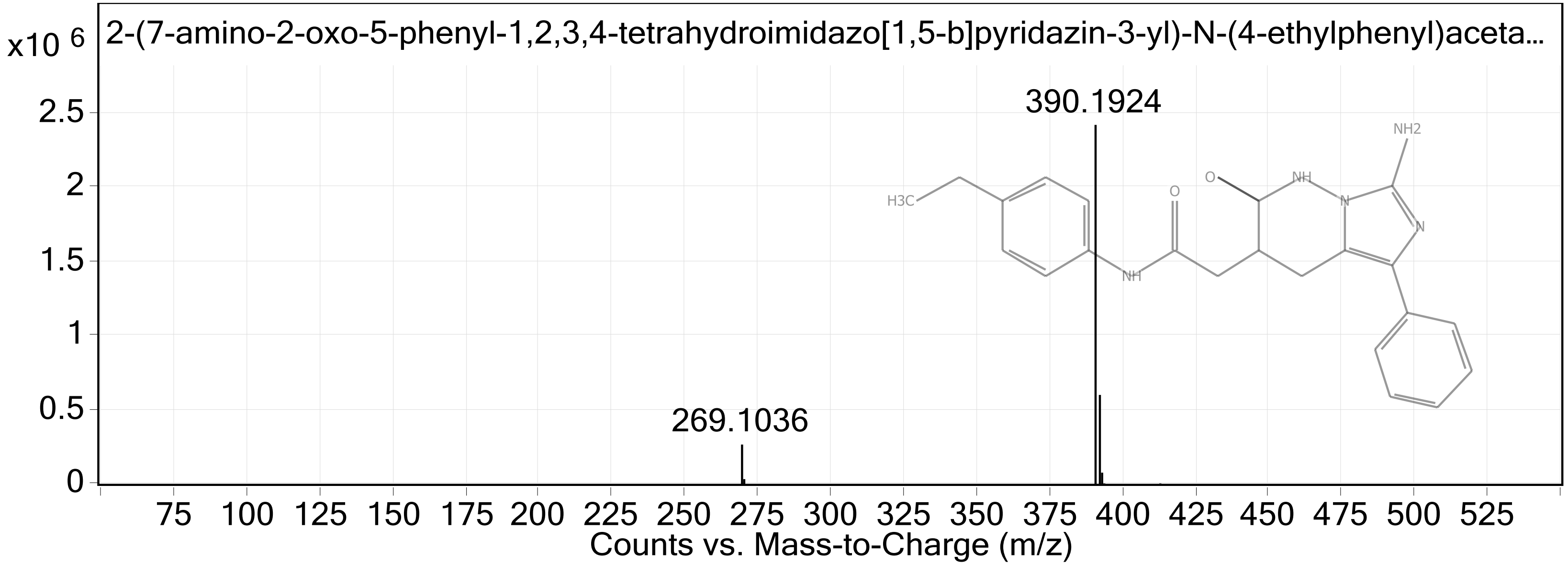

Peak List

m/z	z	Abund
269,1036		274114,59
390,1924	1	2423017
391,1961	1	614791,75

Spectrum Structure

2-(7-amino-2-oxo-5-phenyl-1,2,3,4-tetrahydroimidazo[1,5-b]pyridazin-3-yl)-N-(4-ethylphenyl)acetamide

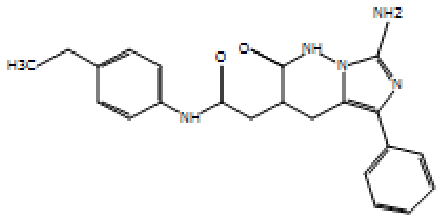

--- End Of Report ---

2-(7-Amino-2-oxo-5-phenyl-1,2,3,4-tetrahydroimidazo[1,5-*b*]pyridazin-3-yl)-*N*-(3-chlorophenyl)acetamide (9c)

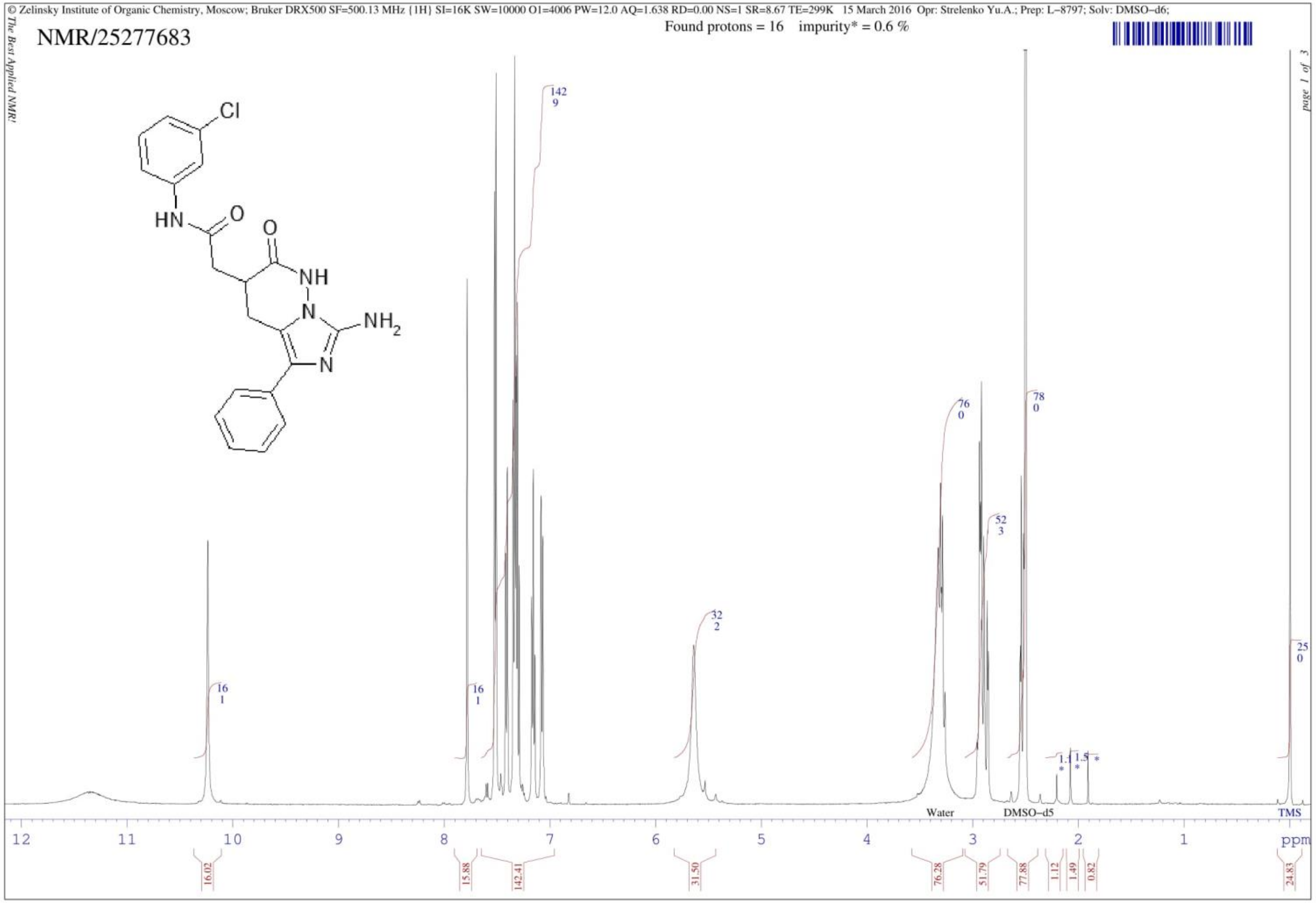

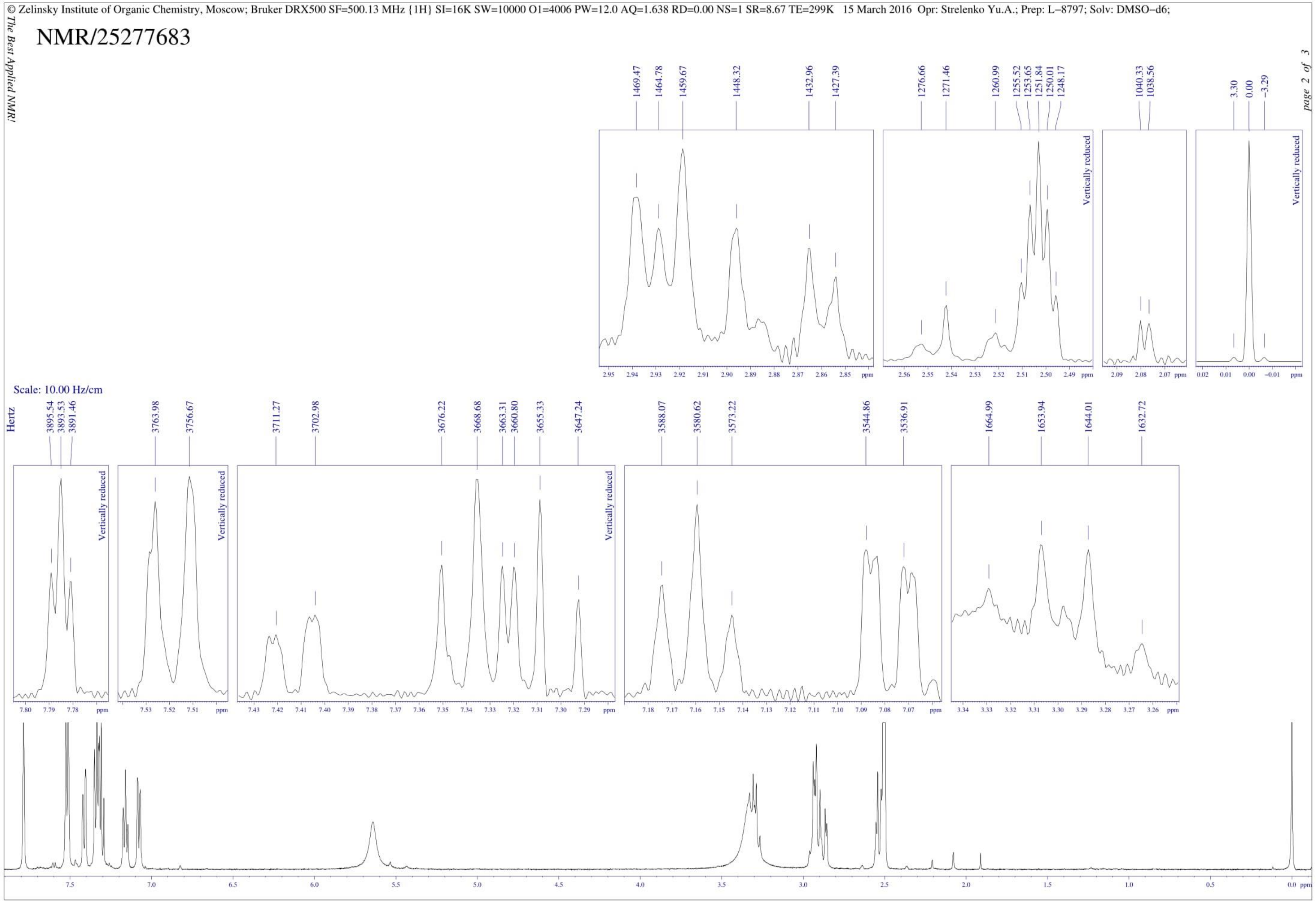
© Zelinsky Institute of Organic Chemistry, Moscow; Bruker DRX500 SF=500.13 MHz {1H} SI=16K SW=10000 O1=4006 PW=12.0 AQ=1.638 RD=0.00 NS=1 SR=8.67 TE=299K 15 March 2016 Opr: Strelenko Yu.A.; Prep: L–8797; Solv: DMSO–d6;
The Best Applied NMR!
NMR/25277683
page 2 of 3
1469.47 1464.78 1459.67 1448.32 1432.96 1427.39
1276.66 1271.46 1260.99 1255.52 1253.65 1251.84 1250.01 1248.17
1040.33 1038.56
3.30 0.00 -3.29
Vertically reduced
Scale: 10.00 Hz/cm
Hertz
3895.54 3893.53 3891.46
3763.98 3756.67
3711.27 3702.98 3676.22 3668.68 3663.31 3660.80 3655.33 3647.24
3588.07 3580.62 3573.22 3544.86 3536.91
1664.99 1653.94 1644.01 1632.72
ppm

© Zelinsky Institute of Organic Chemistry, Moscow; Bruker DRX500 SF=500.13 MHz {1H} SI=16K SW=10000 O1=4006 PW=12.0 AQ=1.638 RD=0.00 NS=1 SR=8.67 TE=299K 15 March 2016 Opr: Strelenko Yu.A.; Prep: L–8797; Solv: DMSO–d6;

The Best Applied NMR!

NMR/25277683

Peaks List

#	Address [points]	Frequency [Hz]	[ppm]	Intensity [cm]
1	12699.4	5121.342	10.2400	2.53
2	16716.1	3895.542	7.7891	3.87
3	16722.7	3893.528	7.7850	6.85
4	16729.5	3891.456	7.7809	3.68
5	17147.2	3763.982	7.5260	7.14
6	17171.2	3756.669	7.5114	8.03
7	17319.9	3711.274	7.4206	2.55
8	17347.1	3702.976	7.4040	3.35
9	17434.8	3676.219	7.3505	5.43
10	17459.5	3668.676	7.3354	9.02
11	17477.1	3663.314	7.3247	5.35
12	17485.3	3660.801	7.3197	5.35
13	17503.2	3655.334	7.3088	8.12
14	17529.7	3647.235	7.2926	4.02
15	17723.6	3588.070	7.1743	2.39
16	17748.0	3580.619	7.1594	4.08
17	17772.3	3573.222	7.1446	1.74
18	17865.2	3544.865	7.0879	3.12
19	17891.3	3536.909	7.0720	2.76
20	20237.5	2820.889	5.6403	1.37
21	24025.2	1664.990	3.3291	2.30
22	24061.4	1653.941	3.3070	3.24
23	24093.9	1644.008	3.2872	3.12
24	24130.9	1632.723	3.2646	1.13
25	24665.8	1469.473	2.9382	3.48
26	24681.2	1464.782	2.9288	2.83
27	24698.0	1459.667	2.9186	4.50
28	24735.1	1448.320	2.8959	2.83
29	24785.5	1432.957	2.8652	2.45
30	24803.7	1427.388	2.8540	1.81
31	25297.6	1276.663	2.5527	1.56
32	25314.7	1271.465	2.5423	5.02
33	25349.0	1260.992	2.5213	2.52
34	25366.9	1255.522	2.5104	6.95
35	25373.0	1253.652	2.5067	13.77
36	25379.0	1251.837	2.5030	19.31
37	25385.0	1250.010	2.4994	13.37
38	25391.0	1248.174	2.4957	5.80
39	26072.0	1040.331	2.0801	0.88
40	26077.9	1038.557	2.0766	0.81
41	26348.9	955.839	1.9112	1.13
42	29470.2	3.297	0.0066	0.68
43	29481.0	0.001	0.0000	34.26
44	29491.8	-3.294	-0.0066	0.68

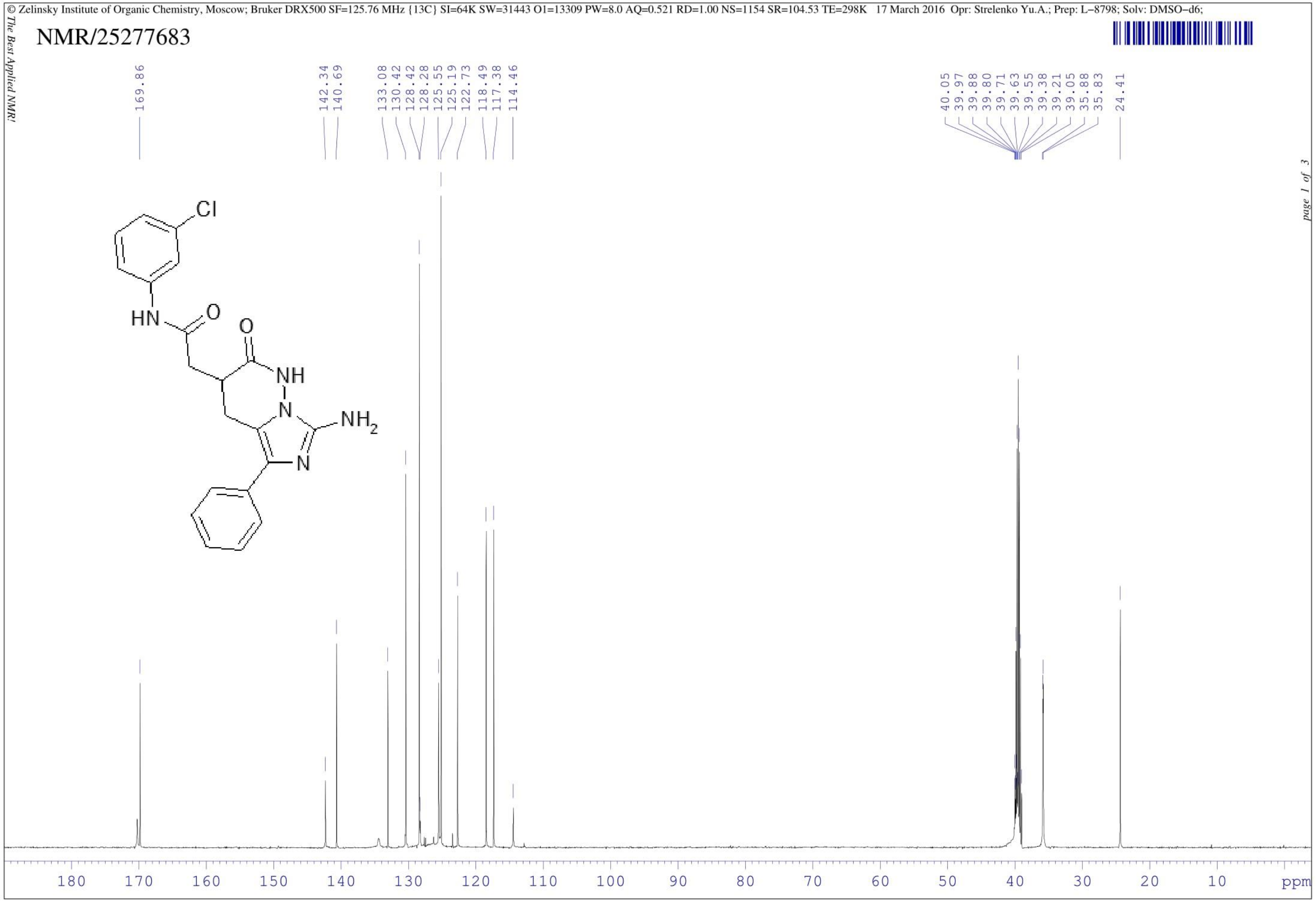
© Zelinsky Institute of Organic Chemistry, Moscow; Bruker DRX500 SF=125.76 MHz {13C} SI=64K SW=31443 O1=13309 PW=8.0 AQ=0.521 RD=1.00 NS=1154 SR=104.53 TE=298K 17 March 2016 Opr: Strelenko Yu.A.; Prep: L–8798; Solv: DMSO–d6;
The Best Applied NMR!
NMR/25277683
169.86
142.34
140.69
133.08
130.42
128.42
128.28
125.55
125.19
122.73
118.49
117.38
114.46
40.05
39.97
39.88
39.80
39.71
39.63
39.55
39.38
39.21
39.05
35.88
35.83
24.41
page 1 of 3
Cl
HN
O
O
NH
N
NH2
N
180
170
160
150
140
130
120
110
100
90
80
70
60
50
40
30
20
10
ppm

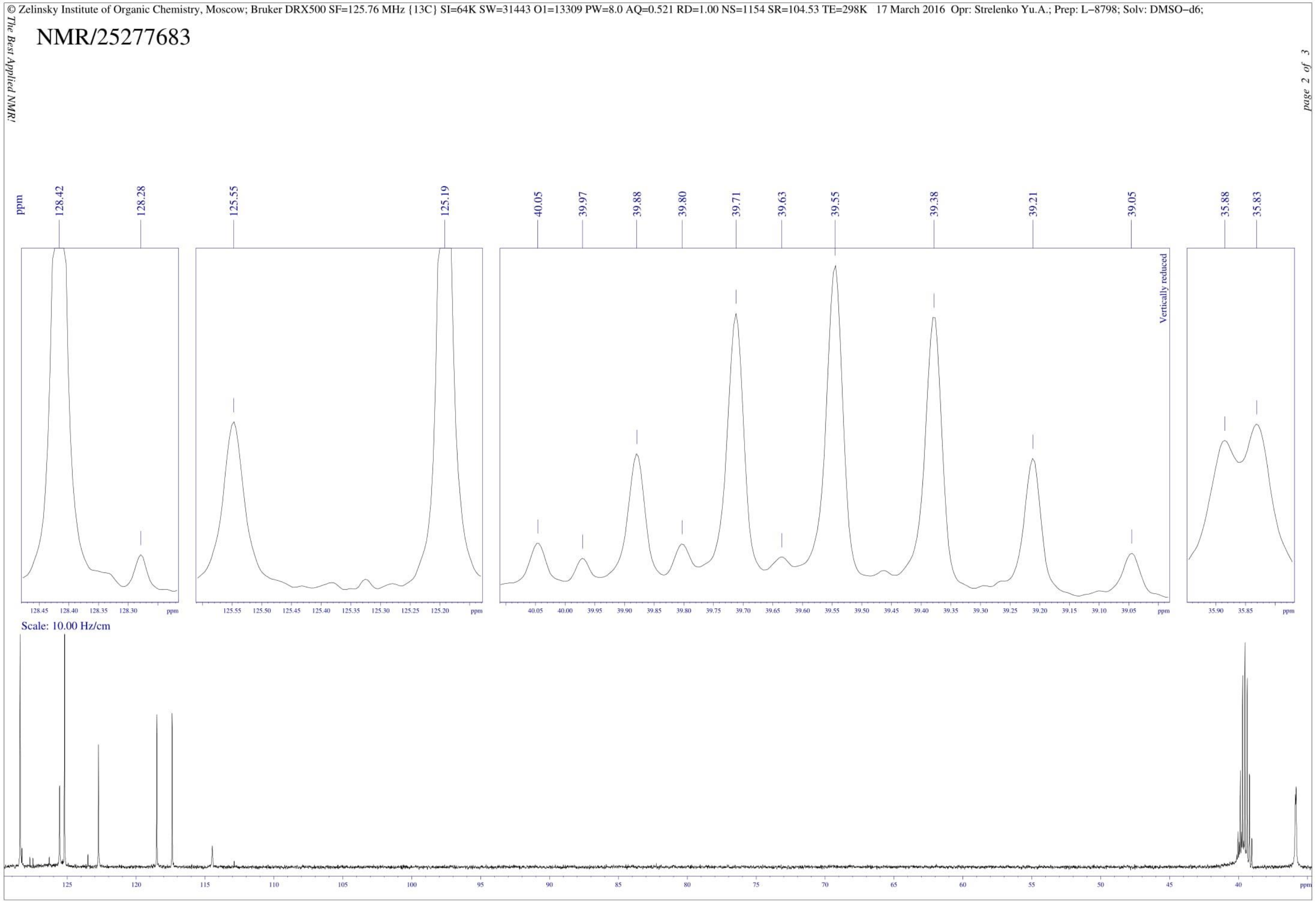
© Zelinsky Institute of Organic Chemistry, Moscow; Bruker DRX500 SF=125.76 MHz {13C} SI=64K SW=31443 O1=13309 PW=8.0 AQ=0.521 RD=1.00 NS=1154 SR=104.53 TE=298K 17 March 2016 Opr: Strelenko Yu.A.; Prep: L–8798; Solv: DMSO–d6;
The Best Applied NMR!
NMR/25277683
page 2 of 3
ppm
128.42
128.28
125.55
125.19
40.05
39.97
39.88
39.80
39.71
39.63
39.55
39.38
39.21
39.05
35.88
35.83
Vertically reduced
Scale: 10.00 Hz/cm

© Zelinsky Institute of Organic Chemistry, Moscow; Bruker DRX500 SF=125.76 MHz {13C} SI=64K SW=31443 O1=13309 PW=8.0 AQ=0.521 RD=1.00 NS=1154 SR=104.53 TE=298K 17 March 2016 Opr: Strelenko Yu.A.; Prep: L–8798; Solv: DMSO–d6;

The Best Applied NMR!

NMR/25277683

Peaks List

#	Address [points]	Frequency [Hz]	Frequency [ppm]	Intensity [cm]
1	15768.0	21361.770	169.8643	3.68
2	22981.4	17900.531	142.3413	1.57
3	23413.4	17693.268	140.6932	4.58
4	25407.9	16736.201	133.0828	3.94
5	26105.6	16401.457	130.4209	8.21
6	26631.0	16149.346	128.4162	12.56
7	26667.0	16132.039	128.2786	0.86
8	27382.8	15788.574	125.5474	3.67
9	27476.1	15743.842	125.1917	14.00
10	28121.4	15434.201	122.7295	5.56
11	29231.5	14901.540	118.4939	6.96
12	29523.7	14761.304	117.3788	6.97
13	30287.5	14394.834	114.4647	0.97
14	49791.5	5036.087	40.0459	1.61
15	49811.3	5026.607	39.9705	1.14
16	49835.2	5015.112	39.8791	4.35
17	49855.3	5005.494	39.8026	1.58
18	49879.0	4994.107	39.7121	8.64
19	49899.3	4984.374	39.6347	1.18
20	49922.7	4973.113	39.5452	10.14
21	49966.5	4952.124	39.3783	8.60
22	50010.2	4931.153	39.2115	4.20
23	50053.8	4910.228	39.0451	1.30
24	50882.0	4512.806	35.8849	3.28
25	50896.2	4506.013	35.8309	3.62
26	53890.4	3069.286	24.4063	5.25

**Data Filename**	LCMS_2682.d	**Sample Name**	#1541
**Sample Type**	Sample	**Position**	Vial 5
**Instrument Name**	Instrument 1	**User Name**	Falaleev A.
**Acq Method**	ACN-H2O_40-60.m	**Acquired Time**	22-Dec-16 7:3
**IRM Calibration Status**	Success	**DA Method**	alex20150126
**Comment**	4c / 2-(7-amino-2-oxo-5-phenyl-1,2,3,4-tetrahydroimidazo[1,5-b]pyridazin-3-yl)-N-(3-chlorophenyl)acetamide		

**Stream Name**	LC 1	**Acquisition SW Version**	6200 series TOF/6500 series Q-TOF B.06.01 (B6157)

User Chromatograms

Fragmentor Voltage 191 **Collision Energy** 0 **Ionization Mode** ESI

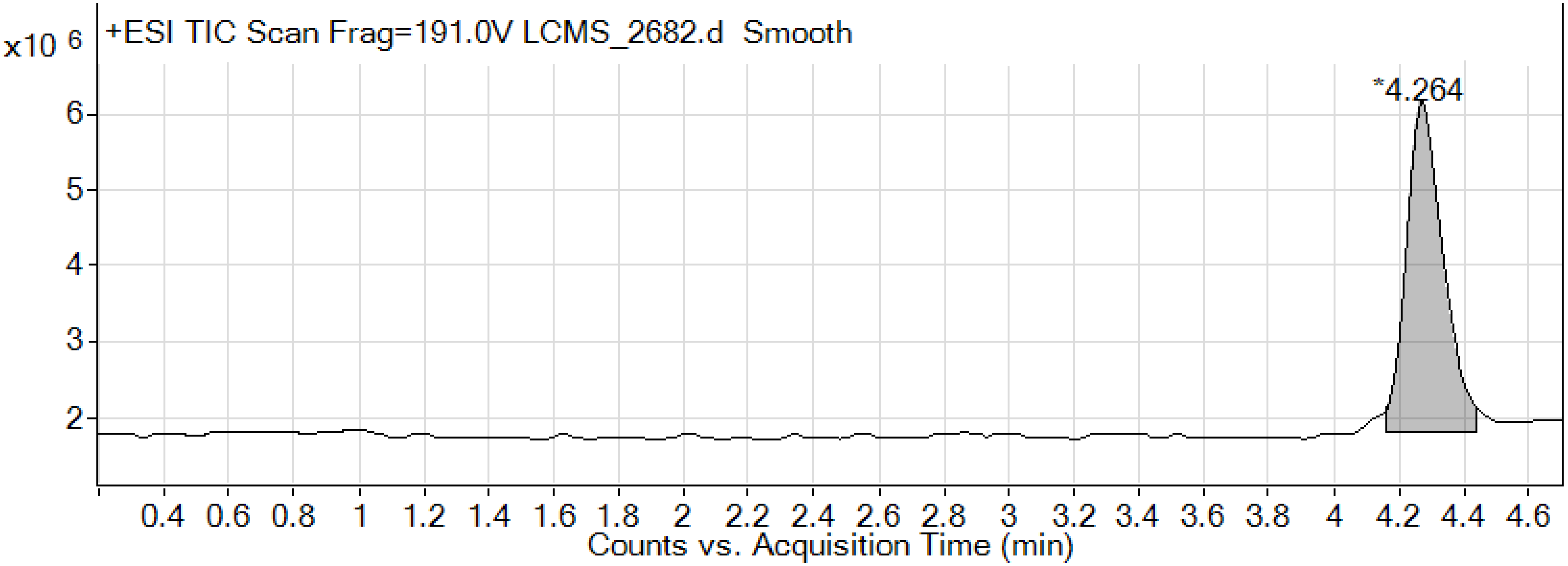

Integration Peak List

Peak	Start	RT	End	Height	Area	Area %
1	4,099	4,264	4,435	4389605,58	34643325,54	100

User Spectra

Spectrum Source	Fragmentor Voltage	Collision Energy	Ionization Mode
Peak (1) in "+ TIC Scan Smo"	191	0	ESI

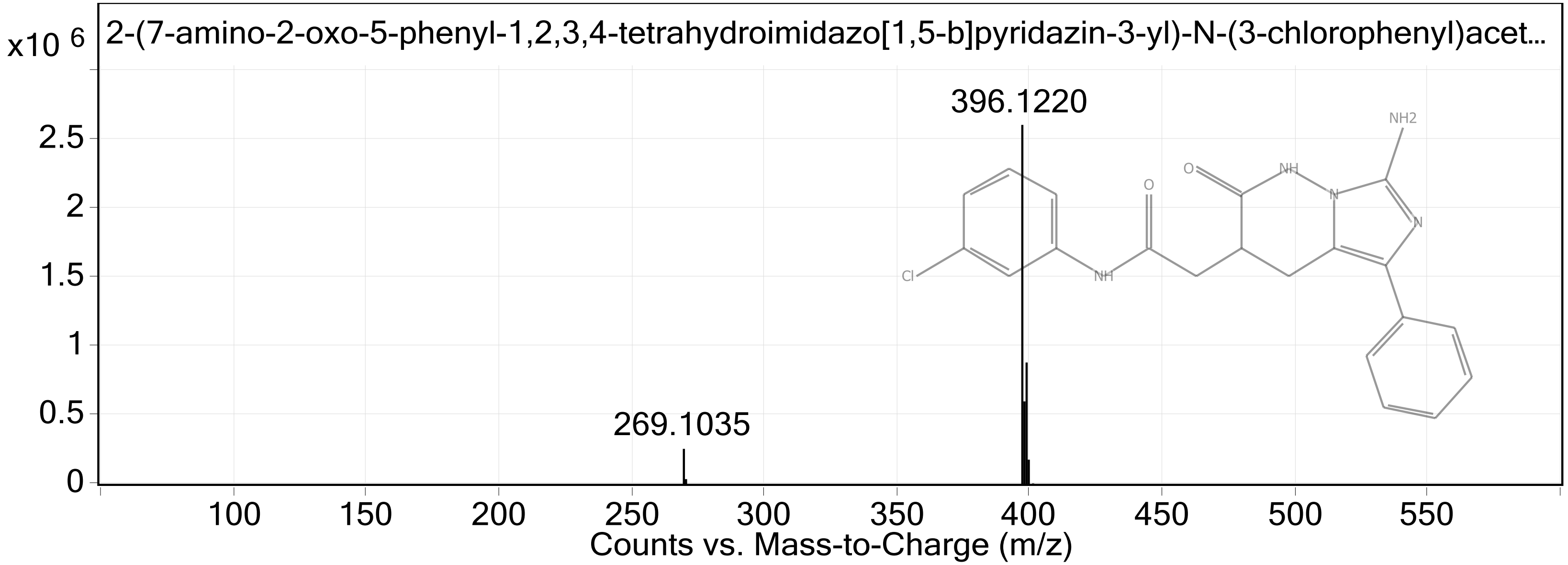

Peak List

m/z	z	Abund
269,1035		268470,53
396,122	1	2618581,25
397,1258	1	607766,44
398,1205	1	891921,63

Spectrum Structure

2-(7-amino-2-oxo-5-phenyl-1,2,3,4-tetrahydroimidazo[1,5-b]pyridazin-3-yl)-N-(3-chlorophenyl)acetamide

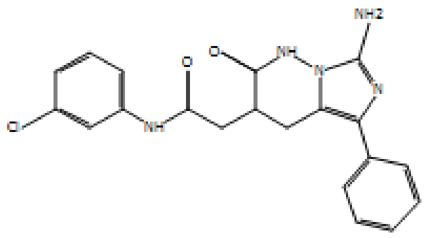

--- End Of Report ---

2-(7-Amino-2-oxo-5-phenyl-1,2,3,4-tetrahydroimidazo[1,5-*b*]pyridazin-3-yl)-*N*-(4-chlorophenyl)acetamide (9d)

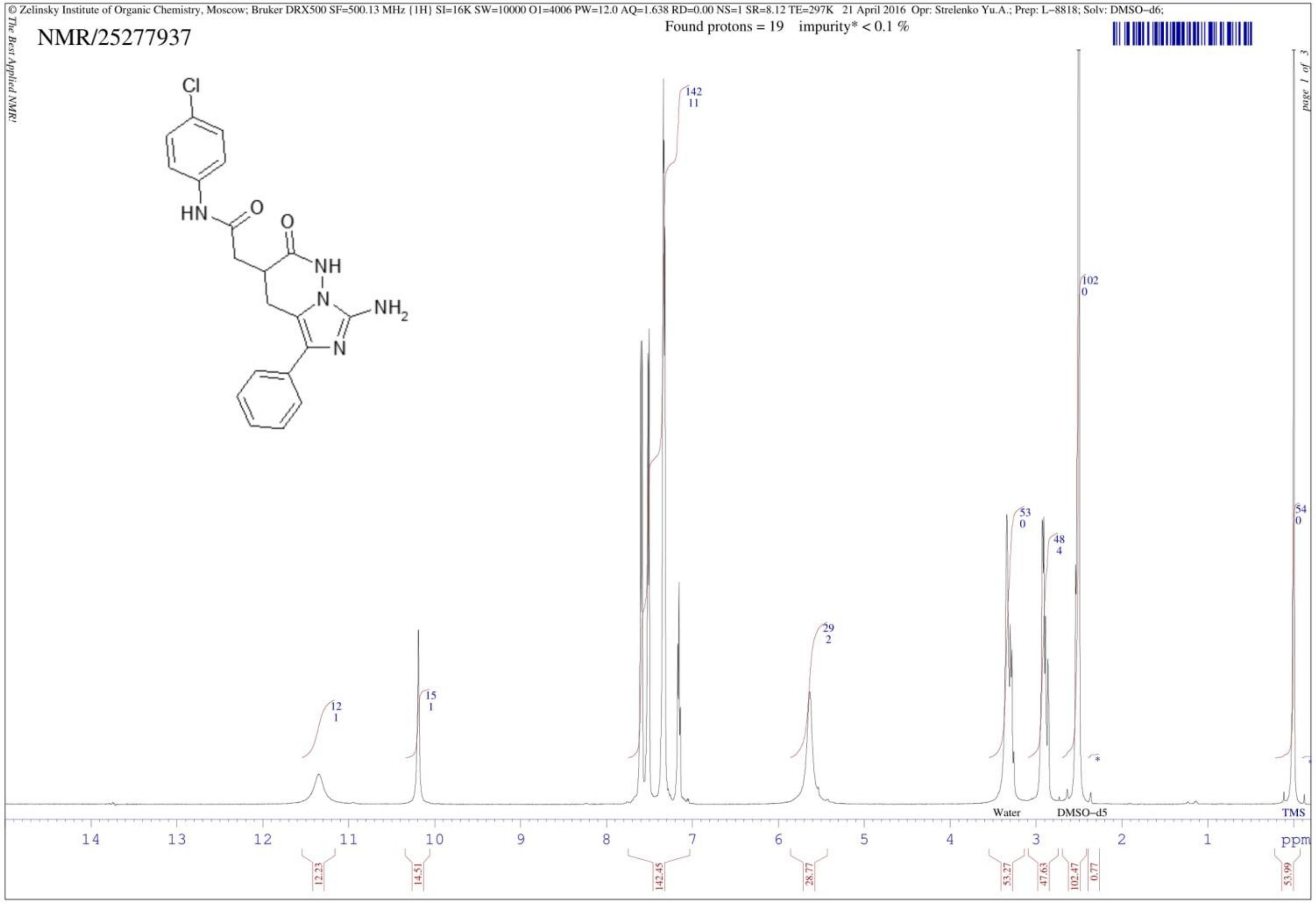

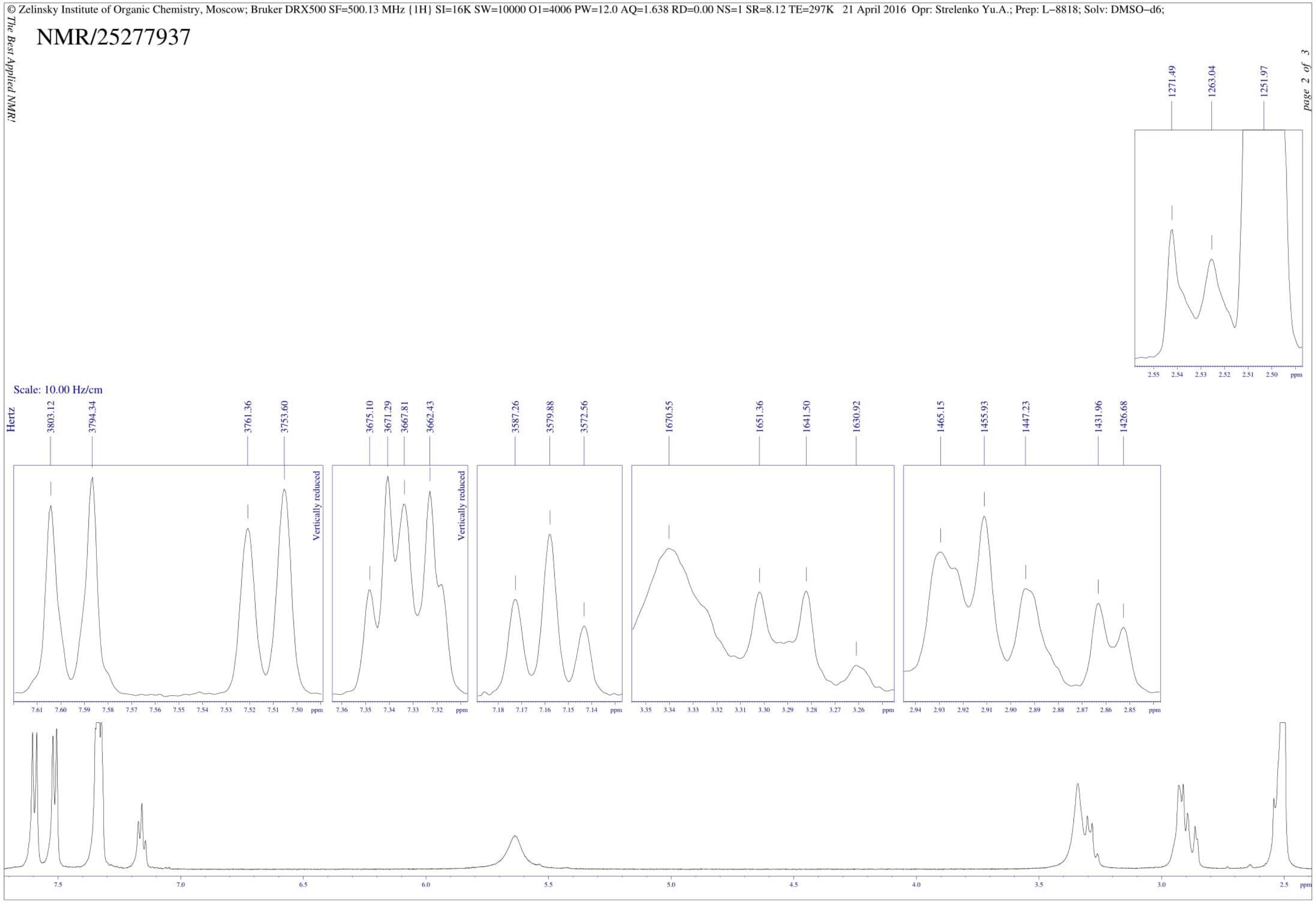
© Zelinsky Institute of Organic Chemistry, Moscow; Bruker DRX500 SF=500.13 MHz {1H} SI=16K SW=10000 O1=4006 PW=12.0 AQ=1.638 RD=0.00 NS=1 SR=8.12 TE=297K 21 April 2016 Opr: Strelenko Yu.A.; Prep: L–8818; Solv: DMSO–d6;
The Best Applied NMR!
NMR/25277937
page 2 of 3
1271.49
1263.04
1251.97
Scale: 10.00 Hz/cm
Hertz
3803.12
3794.34
3761.36
3753.60
Vertically reduced
3675.10
3671.29
3667.81
3662.43
Vertically reduced
3587.26
3579.88
3572.56
1670.55
1651.36
1641.50
1630.92
1465.15
1455.93
1447.23
1431.96
1426.68
ppm

The Best Applied NMR!

NMR/25277937

Peaks List

#	Address [points]	Frequency [Hz]	Frequency [ppm]	Intensity [cm]
1	10883.2	5676.515	11.3501	0.32
2	12782.0	5097.041	10.1914	2.22
3	17021.9	3803.118	7.6043	7.22
4	17050.7	3794.338	7.5867	8.32
5	17158.8	3761.363	7.5208	6.37
6	17184.2	3753.604	7.5053	7.84
7	17441.4	3675.100	7.3483	4.63
8	17453.9	3671.291	7.3407	9.48
9	17465.3	3667.809	7.3337	8.31
10	17483.0	3662.427	7.3229	8.84
11	17729.3	3587.265	7.1727	2.07
12	17753.5	3579.879	7.1579	3.46
13	17777.4	3572.561	7.1433	1.50
14	20254.6	2816.578	5.6317	1.14
15	24010.0	1670.546	3.3402	3.14
16	24072.8	1651.359	3.3019	2.22
17	24105.1	1641.500	3.2821	2.24
18	24139.8	1630.920	3.2610	0.67
19	24683.0	1465.155	2.9295	3.06
20	24713.2	1455.934	2.9111	3.83
21	24741.7	1447.229	2.8937	2.29
22	24791.8	1431.957	2.8632	1.98
23	24809.1	1426.678	2.8526	1.47
24	25162.6	1318.783	2.6369	0.23
25	25317.6	1271.489	2.5423	2.81
26	25345.3	1263.035	2.5254	2.17
27	25381.5	1251.973	2.5033	25.58
28	29292.9	58.329	0.1166	0.25
29	29484.0	0.002	0.0000	45.67
30	29680.1	-59.840	-0.1196	0.22

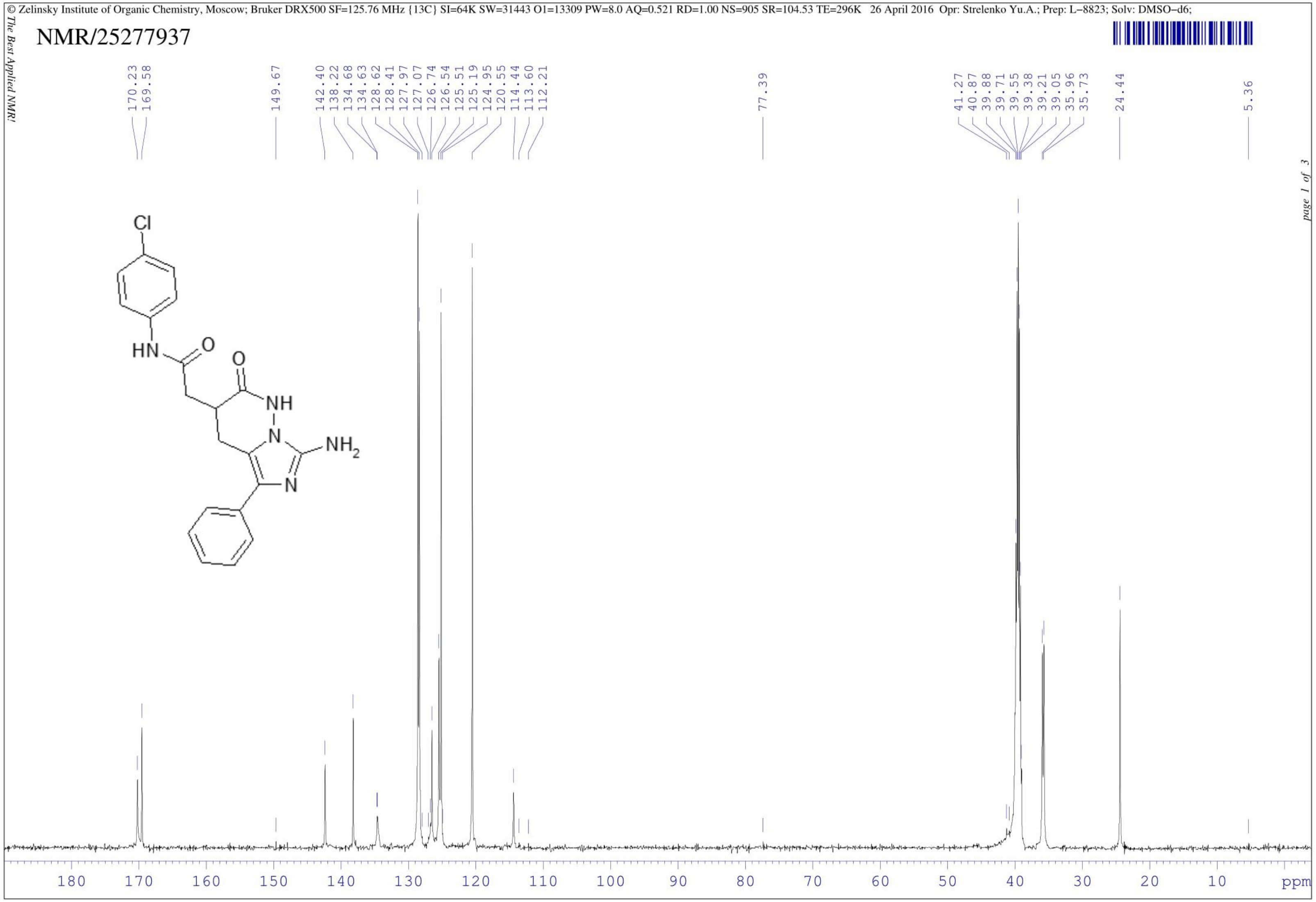
© Zelinsky Institute of Organic Chemistry, Moscow; Bruker DRX500 SF=125.76 MHz {13C} SI=64K SW=31443 O1=13309 PW=8.0 AQ=0.521 RD=1.00 NS=905 SR=104.53 TE=296K 26 April 2016 Opr: Strelenko Yu.A.; Prep: L−8823; Solv: DMSO−d6;
The Best Applied NMR!
NMR/25277937
170.23
169.58
149.67
142.40
138.22
134.68
134.63
128.62
128.41
127.97
127.07
126.74
126.54
125.51
125.19
124.95
120.55
114.44
113.60
112.21
77.39
41.27
40.87
39.88
39.71
39.55
39.38
39.21
39.05
35.96
35.73
24.44
5.36
page 1 of 3
Cl
HN
O
O
NH
N
NH2
N
180
170
160
150
140
130
120
110
100
90
80
70
60
50
40
30
20
10
ppm

© Zelinsky Institute of Organic Chemistry, Moscow; Bruker DRX500 SF=125.76 MHz {13C} SI=64K SW=31443 O1=13309 PW=8.0 AQ=0.521 RD=1.00 NS=905 SR=104.53 TE=296K 26 April 2016 Opr: Strelenko Yu.A.; Prep: L−8823; Solv: DMSO−d6;

NMR/25277937

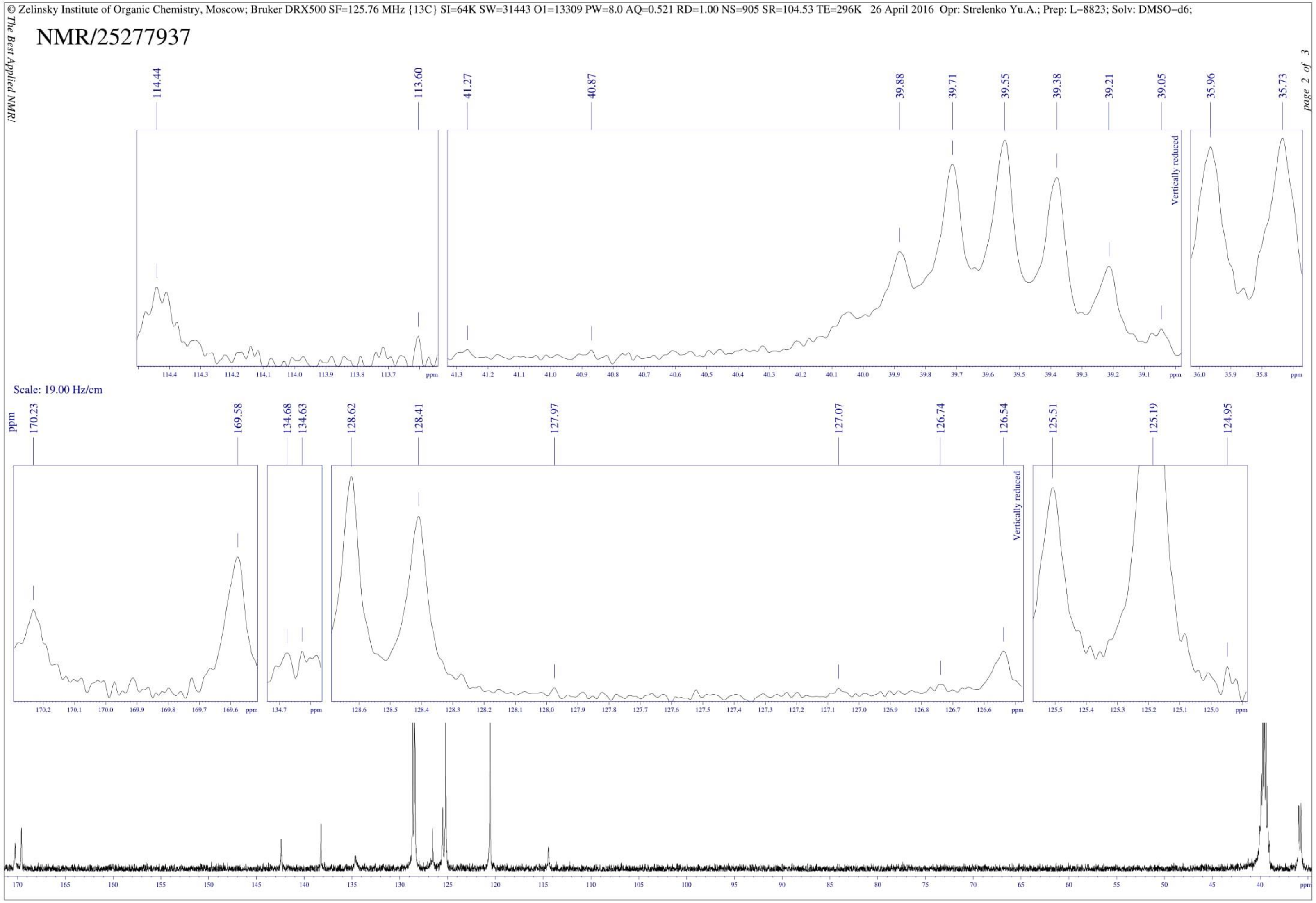

© Zelinsky Institute of Organic Chemistry, Moscow; Bruker DRX500 SF=125.76 MHz {13C} SI=64K SW=31443 O1=13309 PW=8.0 AQ=0.521 RD=1.00 NS=905 SR=104.53 TE=296K 26 April 2016 Opr: Strelenko Yu.A.; Prep: L−8823; Solv: DMSO−d6;

The Best Applied NMR!

NMR/25277937

Peaks List

#	Address [points]	Frequency [Hz]	Frequency [ppm]	Intensity [cm]
1	15672.0	21407.877	170.2309	1.85
2	15843.4	21325.629	169.5769	2.96
3	21059.9	18822.561	149.6730	0.51
4	22965.4	17908.205	142.4023	2.14
5	24061.1	17382.445	138.2216	3.22
6	24990.6	16936.459	134.6752	0.93
7	25003.4	16930.338	134.6265	0.97
8	26576.4	16175.527	128.6244	14.00
9	26632.8	16148.456	128.4091	11.47
10	26746.7	16093.834	127.9748	0.58
11	26985.0	15979.486	127.0655	0.55
12	27070.4	15938.494	126.7396	0.79
13	27123.4	15913.082	126.5375	2.91
14	27393.4	15783.520	125.5072	4.42
15	27477.7	15743.037	125.1853	11.91
16	27540.0	15713.184	124.9479	0.65
17	28691.9	15160.461	120.5528	12.89
18	30293.7	14391.854	114.4410	1.57
19	30513.0	14286.624	113.6042	0.53
20	30877.1	14111.879	112.2147	0.53
21	40003.4	9732.785	77.3931	0.51
22	49471.6	5189.584	41.2665	0.76
23	49575.8	5139.614	40.8691	0.73
24	49834.2	5015.606	39.8830	6.82
25	49878.5	4994.362	39.7141	12.24
26	49922.5	4973.231	39.5461	13.75
27	49966.1	4952.333	39.3799	11.44
28	50009.7	4931.375	39.2133	5.94
29	50053.7	4910.264	39.0454	2.02
30	50861.2	4522.796	35.9643	4.54
31	50921.6	4493.820	35.7339	4.72
32	53881.4	3073.601	24.4406	5.48
33	58882.9	673.700	5.3571	0.53

© Zelinsky Institute of Organic Chemistry, Moscow; Bruker DRX500 SF=500.13 MHz {1H−13C}HMBC SI=1024x512 SW=7122 O1=3455 PW=14.4 AQ=0.062 RD=1.00 NS=4x256 SR=6.71 TE=297K 13 October 2016 Opr: Strelenko Yu.A.; Prep: L−8891; Solv: DMSO−d6;

NMR/26227577

1H−13C HMBC−qs, DRX500

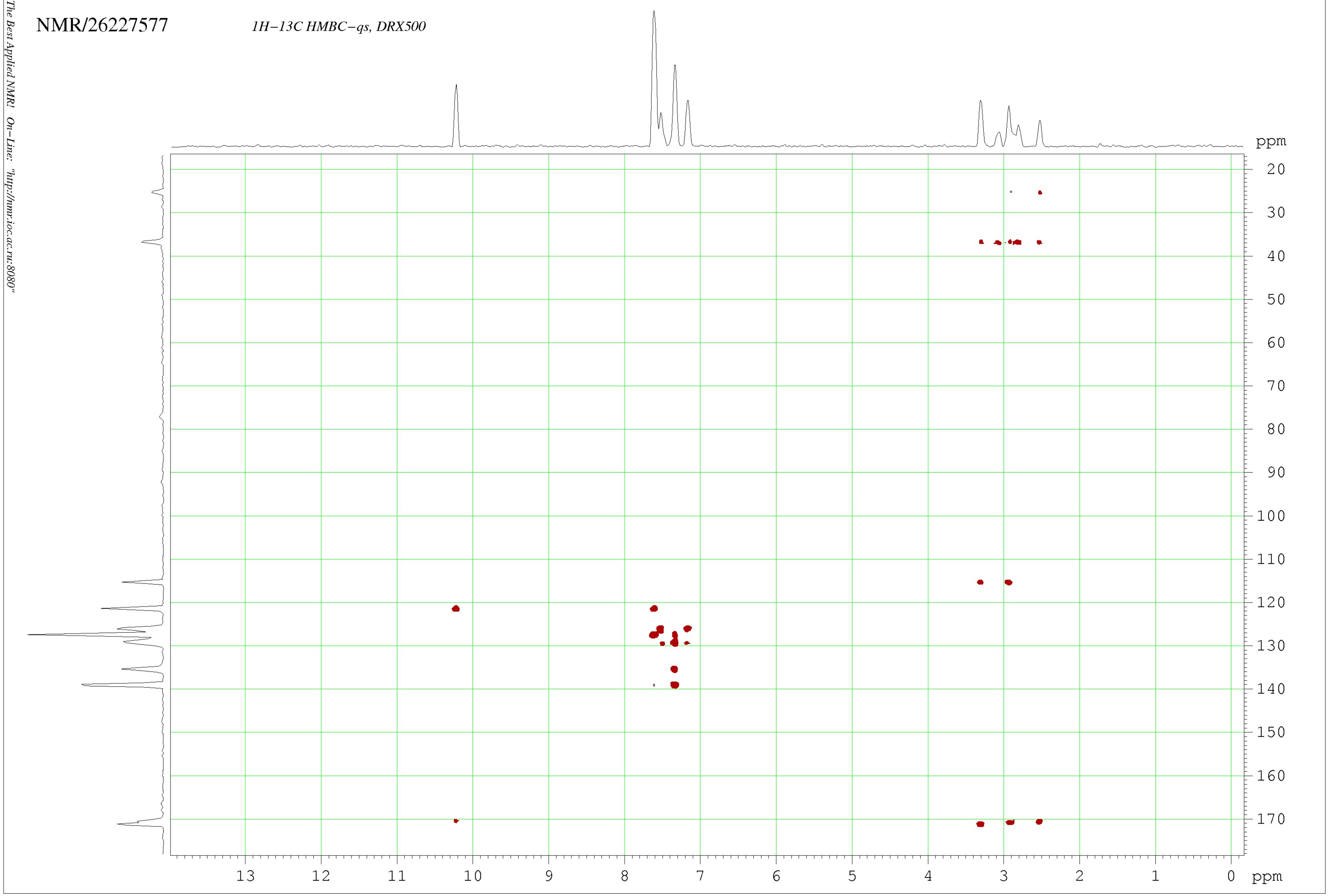

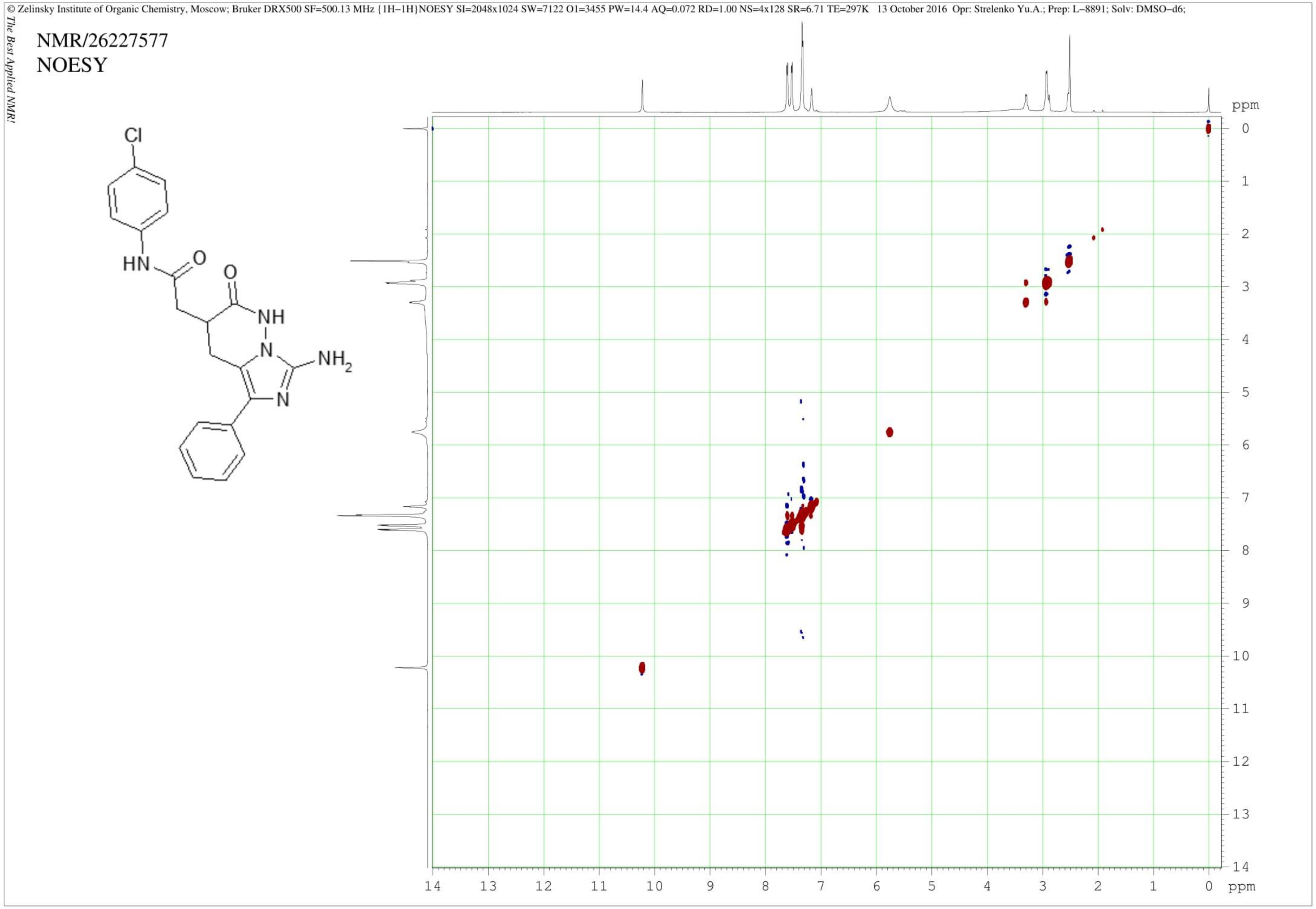
© Zelinsky Institute of Organic Chemistry, Moscow; Bruker DRX500 SF=500.13 MHz {1H−1H}NOESY SI=2048x1024 SW=7122 O1=3455 PW=14.4 AQ=0.072 RD=1.00 NS=4x128 SR=6.71 TE=297K 13 October 2016 Opr: Strelenko Yu.A.; Prep: L−8891; Solv: DMSO−d6;
The Best Applied NMR!
NMR/26227577
NOESY
Cl
HN
O
O
NH
N
NH2
N
ppm
ppm

^{1}H NMR spectrum in the presence of CF_3COOH

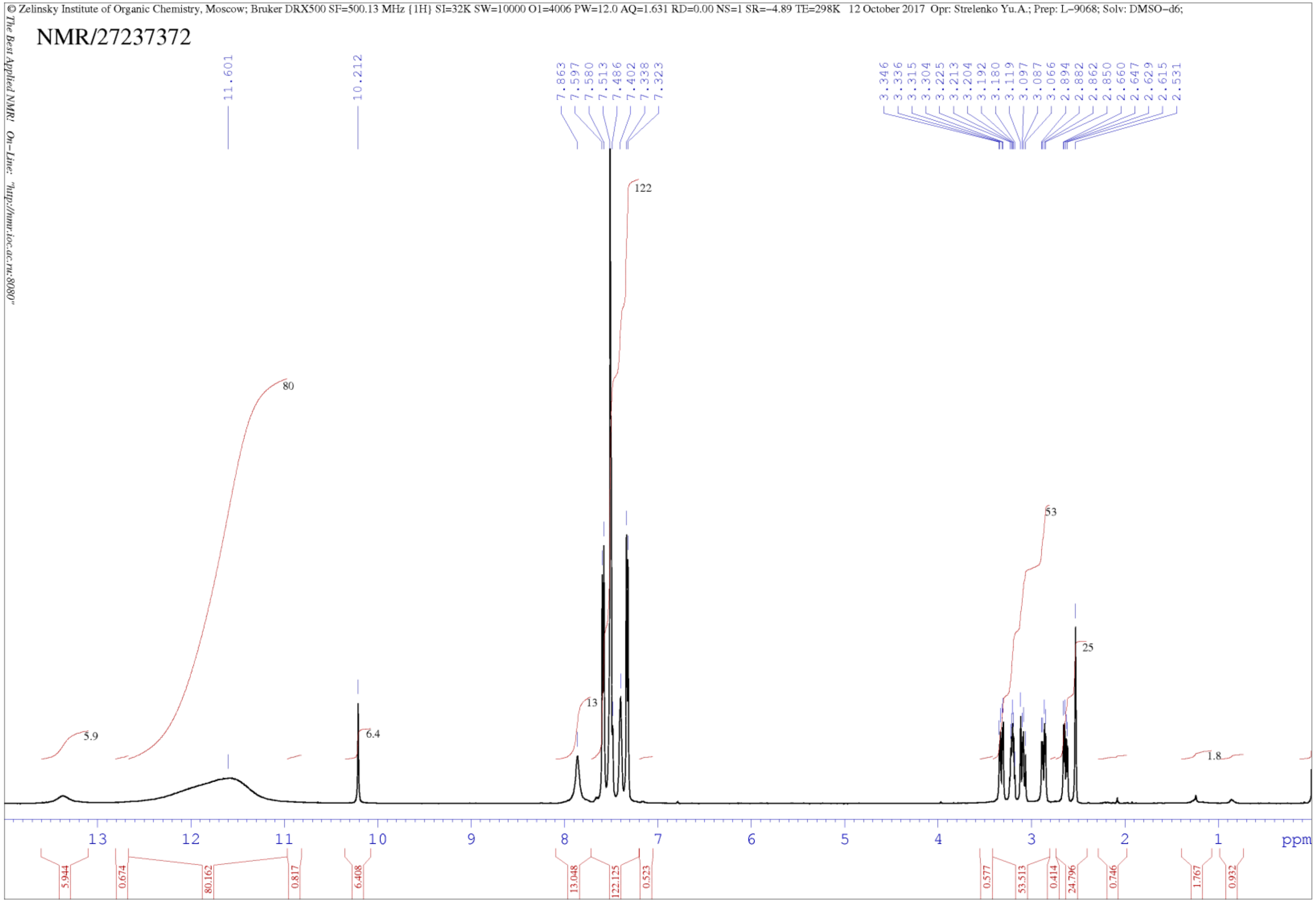

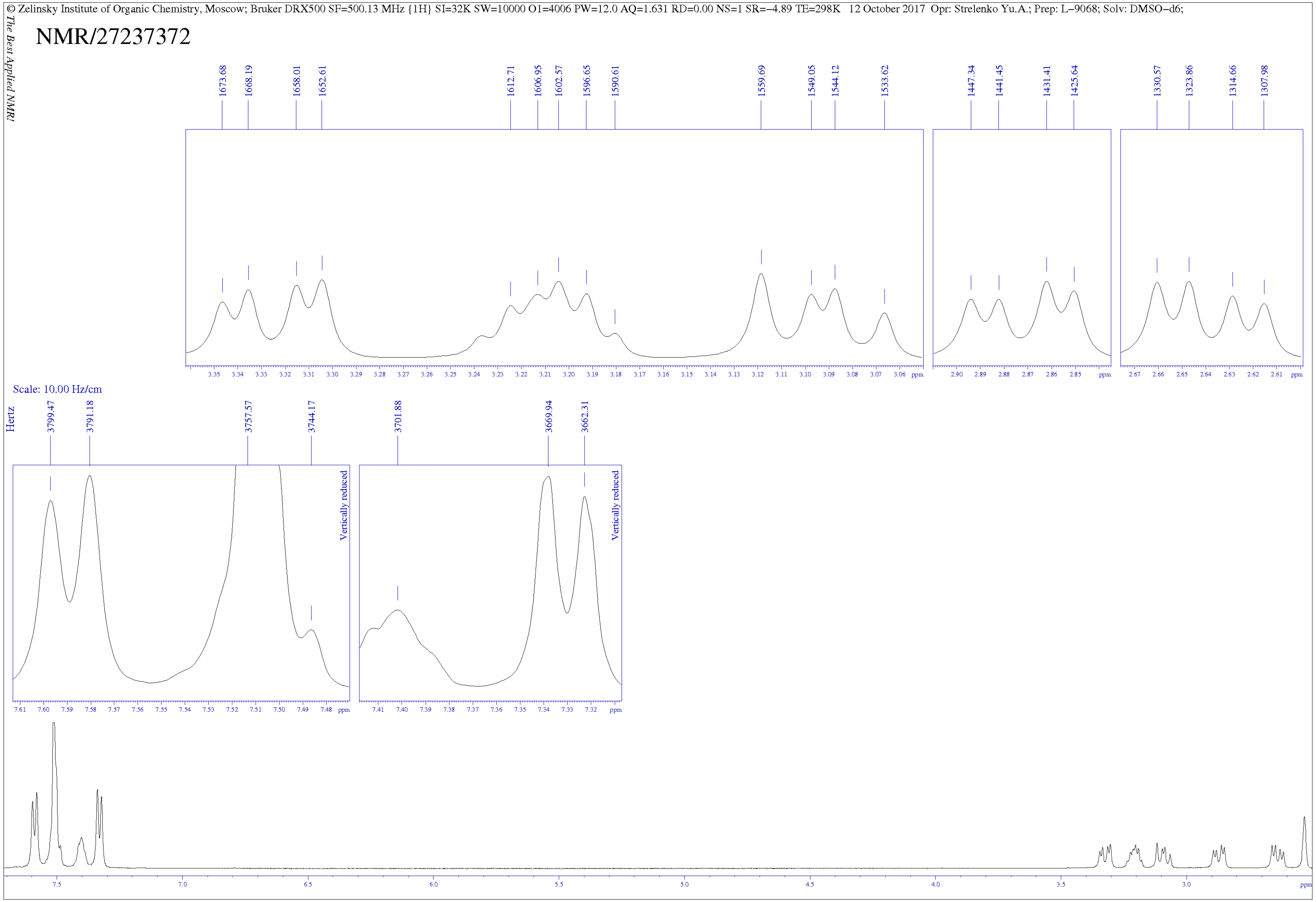
© Zelinsky Institute of Organic Chemistry, Moscow; Bruker DRX500 SF=500.13 MHz {1H} SI=32K SW=10000 O1=4006 PW=12.0 AQ=1.631 RD=0.00 NS=1 SR=−4.89 TE=298K 12 October 2017 Opr: Strelenko Yu.A.; Prep: L−9068; Solv: DMSO−d6;
The Best Applied NMR!
NMR/27237372
Scale: 10.00 Hz/cm
Hertz
Vertically reduced
ppm

**Data Filename**	LCMS_2685.d	**Sample Name**	#1544
**Sample Type**	Sample	**Position**	Vial 8
**Instrument Name**	Instrument 1	**User Name**	Falaleev A.
**Acq Method**	ACN-H2O_40-60.m	**Acquired Time**	22-Dec-16 8:15:10
**IRM Calibration Status**	Success	**DA Method**	alex20150126.m
**Comment**	4f / 2-(7-amino-2-oxo-5-phenyl-1,2,3,4-tetrahydroimidazo[1,5-b]pyridazin-3-yl)-N-(4-chlorophenyl)acetamide		

**Stream Name**	LC 1	**Acquisition SW Version**	6200 series TOF/6500 series Q-TOF B.06.01 (B6157)

User Chromatograms

**Fragmentor Voltage**	191	**Collision Energy**	0	**Ionization Mode**	ESI

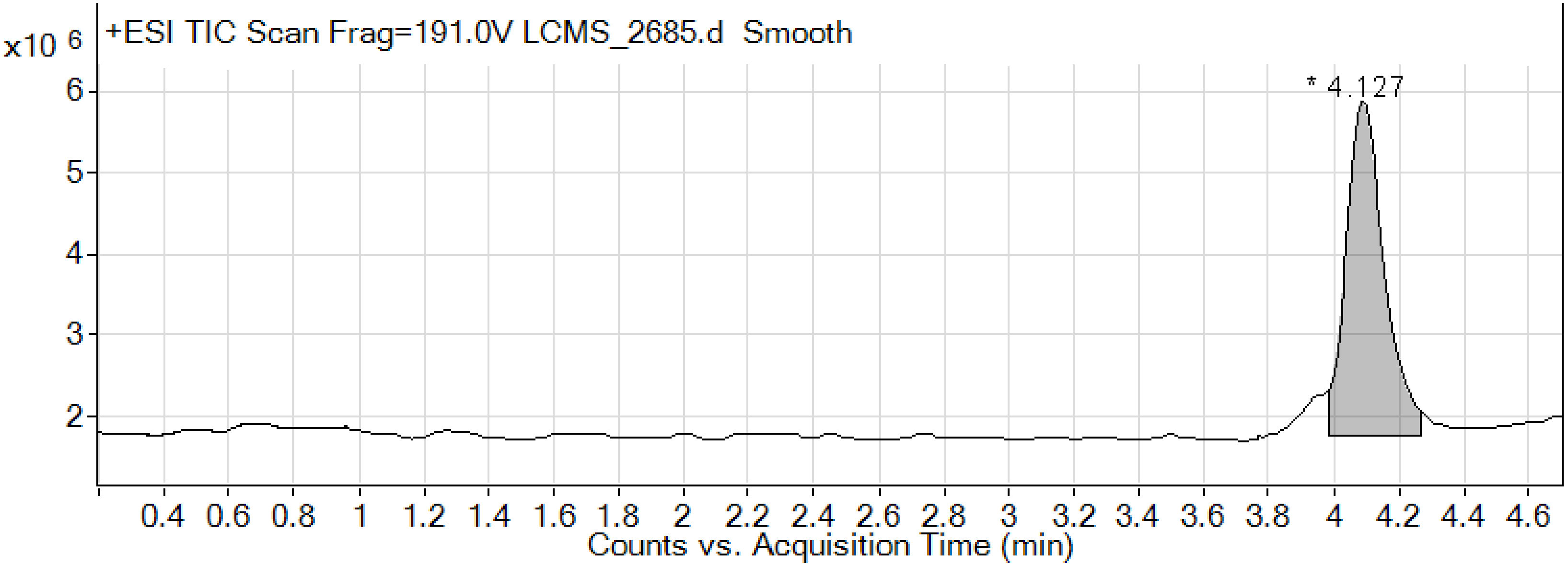

Integration Peak List

Peak	Start	RT	End	Height	Area	Area %
1	3,81	4,127	4,31	4113238,7	33341312,11	100

User Spectra

Spectrum Source	Fragmentor Voltage	Collision Energy	Ionization Mode
Peak (1) in "+ TIC Scan Smo"	191	0	ESI

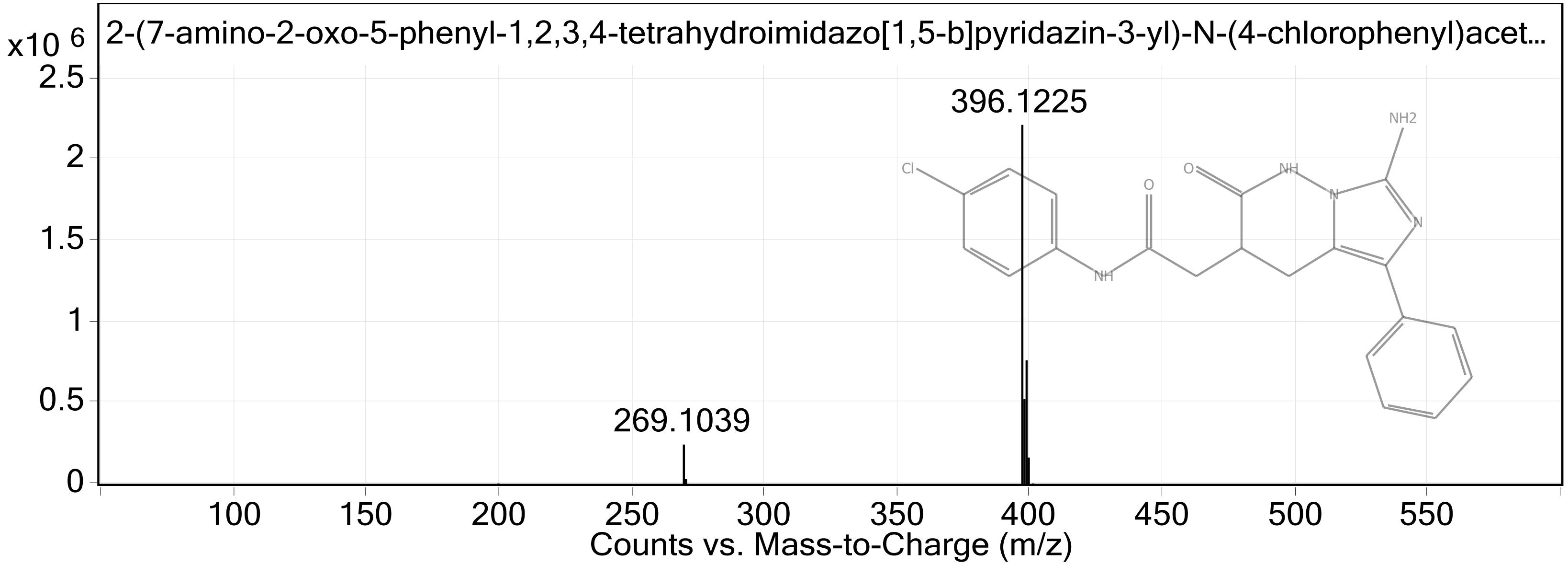

Peak List

m/z	z	Abund
269,1039		250284,86
396,1225	1	2217305,75
397,1262	1	525264,94
398,1208	1	773892,69

Spectrum Structure

2-(7-amino-2-oxo-5-phenyl-1,2,3,4-tetrahydroimidazo[1,5-b]pyridazin-3-yl)-N-(4-chlorophenyl)acetamide

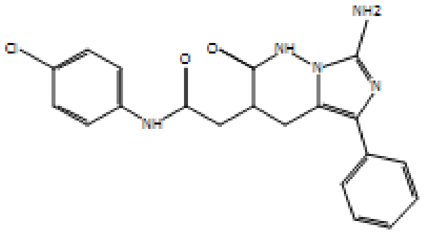

--- End Of Report ---

2-(7-Amino-2-oxo-5-phenyl-1,2,3,4-tetrahydroimidazo[1,5-*b*]pyridazin-3-yl)-*N*-(3,4-dimethylphenyl)acetamide (9e)

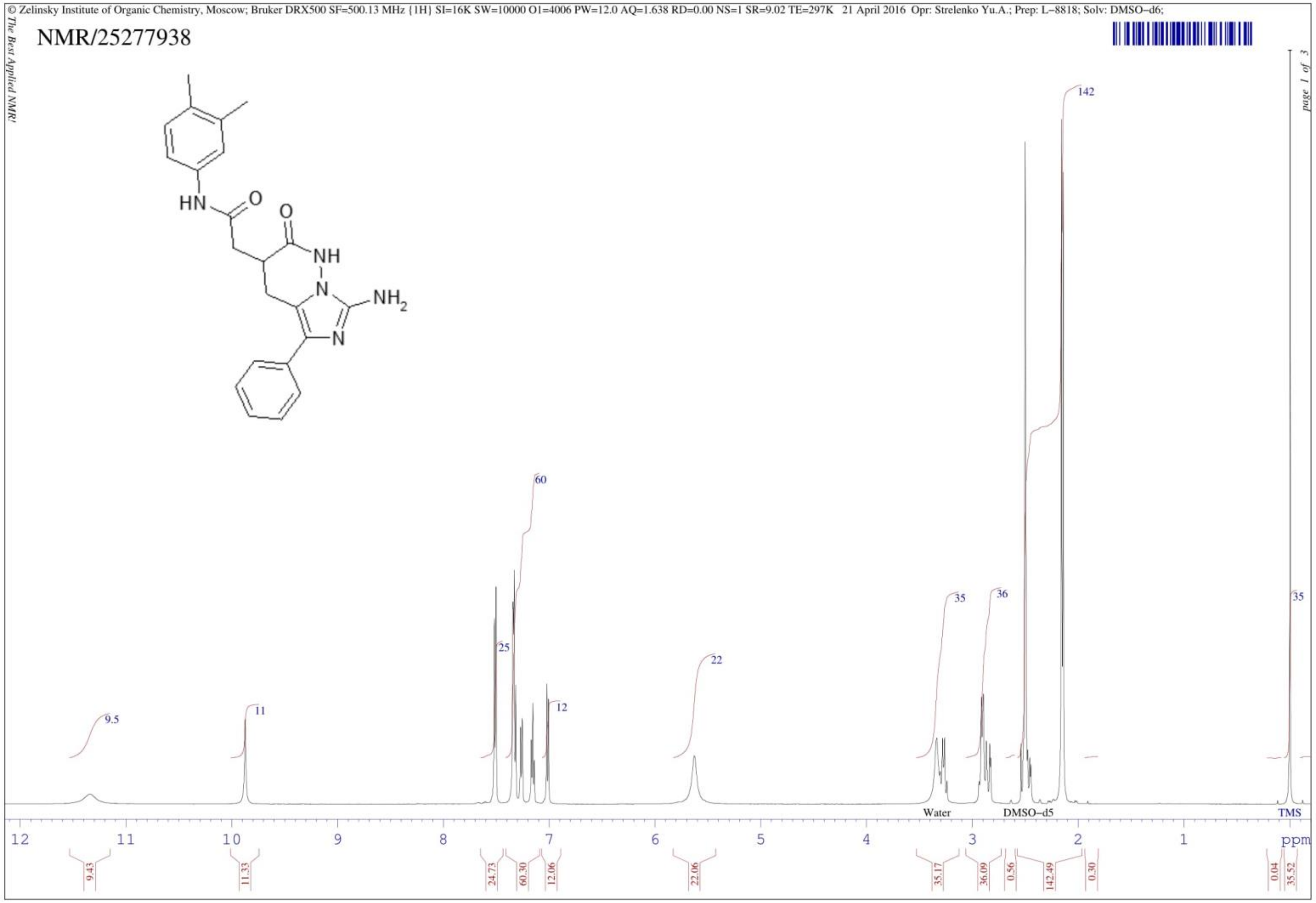

© Zelinsky Institute of Organic Chemistry, Moscow; Bruker DRX500 SF=500.13 MHz {1H} SI=16K SW=10000 O1=4006 PW=12.0 AQ=1.638 RD=0.00 NS=1 SR=9.02 TE=297K 21 April 2016 Opr: Strelenko Yu.A.; Prep: L−8818; Solv: DMSO−d6;

NMR/25277938

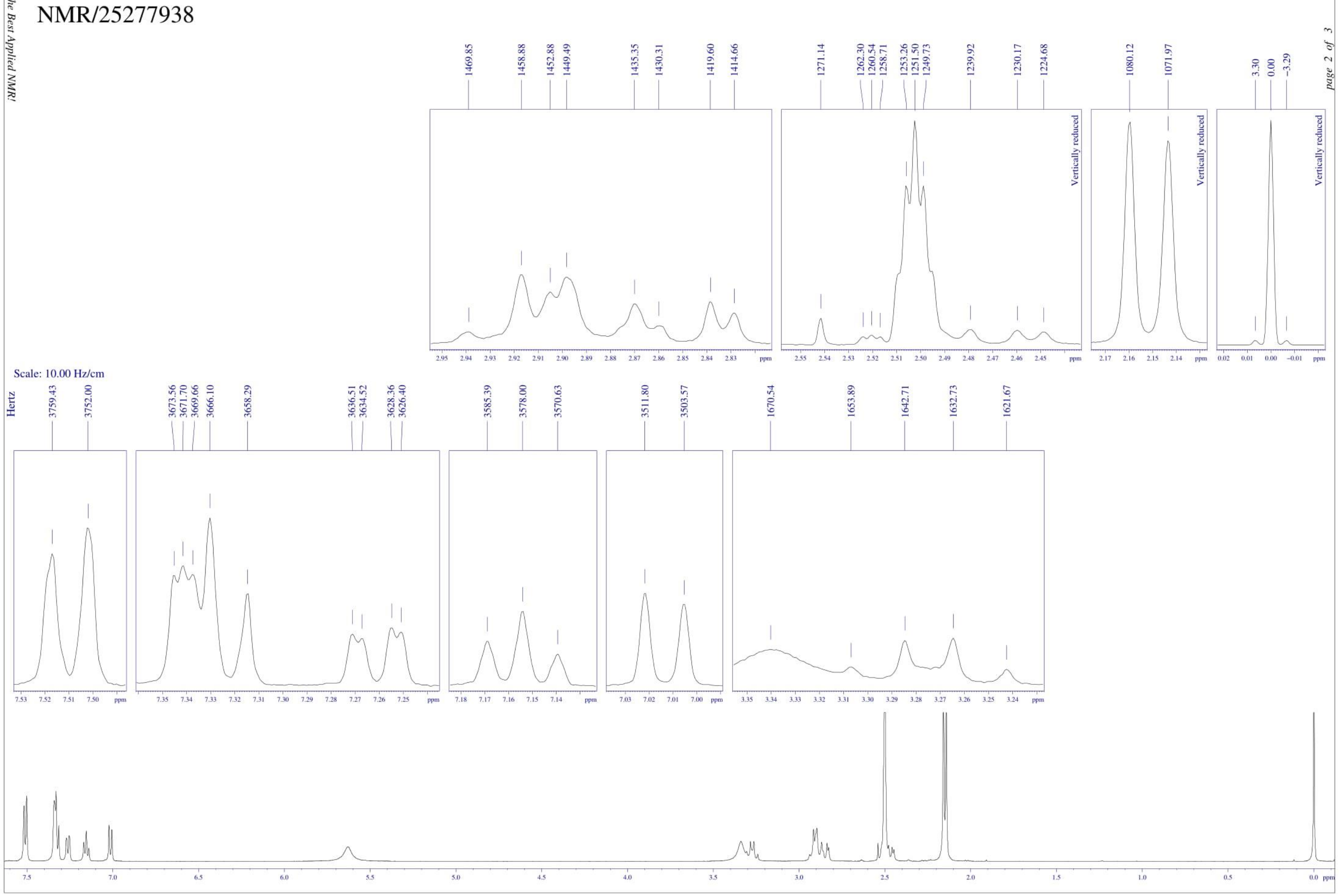

Peaks List

#	Address [points]	Frequency [Hz]	Frequency [ppm]	Intensity [cm]
1	13302.0	4937.438	9.8723	1.13
2	17162.1	3759.426	7.5169	2.76
3	17186.5	3751.997	7.5020	3.31
4	17443.5	3673.560	7.3452	2.33
5	17449.6	3671.703	7.3415	2.51
6	17456.3	3669.660	7.3374	2.33
7	17467.9	3666.101	7.3303	3.50
8	17493.5	3658.286	7.3147	1.95
9	17564.9	3636.505	7.2711	1.09
10	17571.4	3634.521	7.2672	1.00
11	17591.6	3628.365	7.2548	1.22
12	17598.0	3626.403	7.2509	1.13
13	17732.4	3585.394	7.1689	0.95
14	17756.6	3577.999	7.1541	1.57
15	17780.8	3570.626	7.1394	0.67
16	17973.6	3511.795	7.0218	1.95
17	18000.5	3503.572	7.0053	1.72
18	20256.4	2815.122	5.6288	0.56
19	24007.0	1670.539	3.3402	0.77
20	24061.5	1653.890	3.3069	0.40
21	24098.2	1642.714	3.2846	0.96
22	24130.9	1632.731	3.2646	1.00
23	24167.1	1621.671	3.2425	0.35
24	24664.6	1469.848	2.9389	0.28
25	24700.5	1458.883	2.9170	1.46
26	24720.2	1452.879	2.9050	1.10
27	24731.3	1449.488	2.8982	1.42
28	24777.7	1435.349	2.8700	0.86
29	24794.2	1430.314	2.8599	0.40
30	24829.2	1419.604	2.8385	0.90
31	24845.4	1414.663	2.8286	0.67
32	25315.7	1271.144	2.5416	1.19
33	25344.7	1262.302	2.5239	0.37
34	25350.5	1260.536	2.5204	0.44
35	25356.5	1258.706	2.5168	0.35
36	25374.3	1253.259	2.5059	7.02
37	25380.1	1251.496	2.5023	9.83
38	25385.9	1249.735	2.4988	6.99
39	25418.0	1239.917	2.4792	0.68
40	25450.0	1230.170	2.4597	0.65
41	25468.0	1224.681	2.4487	0.58
42	25941.7	1080.116	2.1597	11.18
43	25968.4	1071.975	2.1434	10.25
44	29470.2	3.296	0.0066	0.47
45	29481.0	0.001	0.0000	23.50
46	29491.8	-3.293	-0.0066	0.47

© Zelinsky Institute of Organic Chemistry, Moscow; Bruker DRX500 SF=500.13 MHz [] SI=16K SW=10000 O1=4006 PW=12.0 AQ=1.638 RD=0.00 NS=0 SR=9.02 TE=297K 21 April 2016 Opr: Strelenko Yu.A.; Prep: L-8818; Solv: DMSO-d6;

NMR/25277938

The Best Applied NMR!

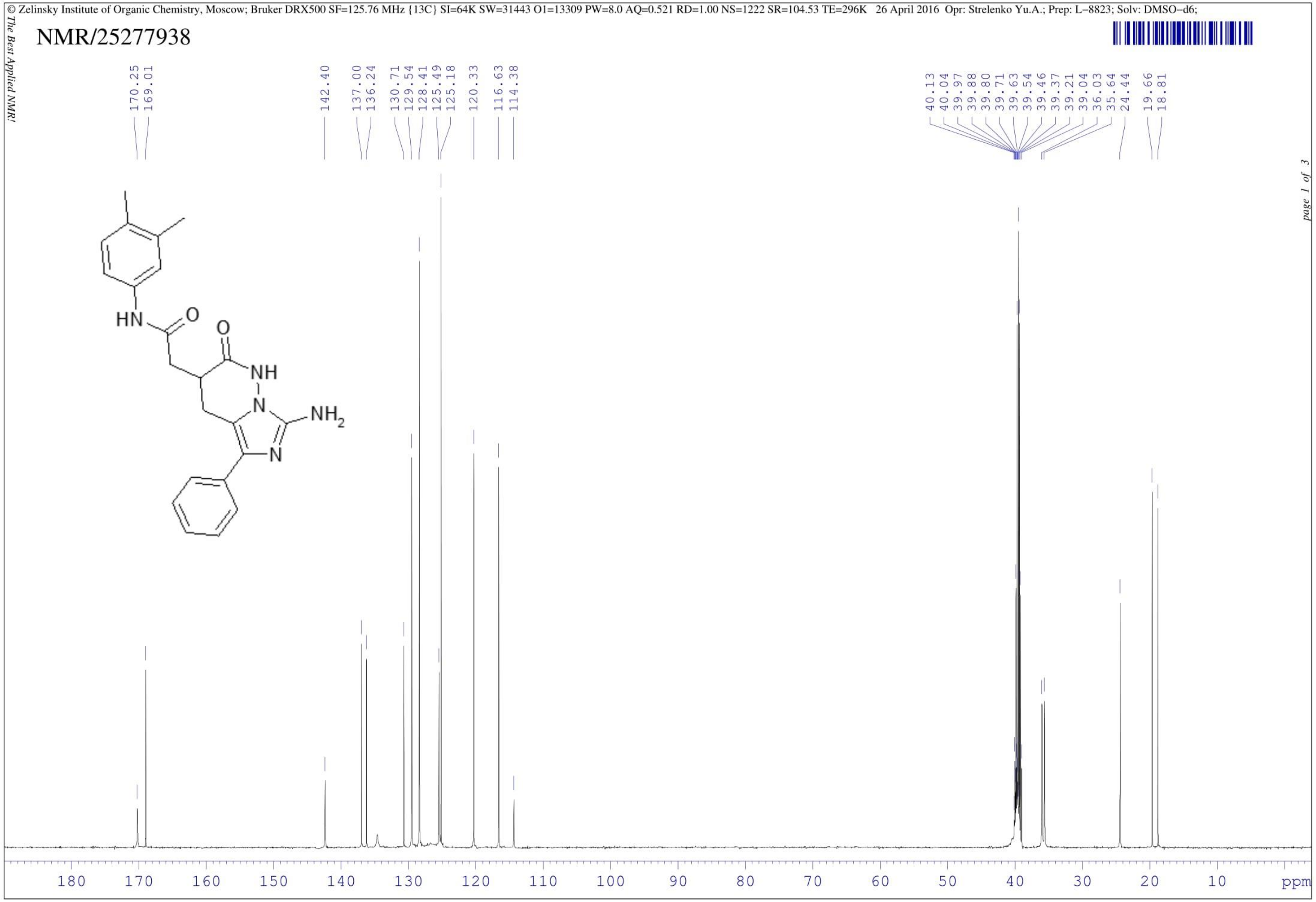
© Zelinsky Institute of Organic Chemistry, Moscow; Bruker DRX500 SF=125.76 MHz {13C} SI=64K SW=31443 O1=13309 PW=8.0 AQ=0.521 RD=1.00 NS=1222 SR=104.53 TE=296K 26 April 2016 Opr: Strelenko Yu.A.; Prep: L−8823; Solv: DMSO−d6;
NMR/25277938
The Best Applied NMR!
page 1 of 3
170.25
169.01
142.40
137.00
136.24
130.71
129.54
128.41
125.49
125.18
120.33
116.63
114.38
40.13
40.04
39.97
39.88
39.80
39.71
39.63
39.54
39.46
39.37
39.21
39.04
36.03
35.64
24.44
19.66
18.81
HN
O
O
NH
N
NH2
N
180 170 160 150 140 130 120 110 100 90 80 70 60 50 40 30 20 10 ppm

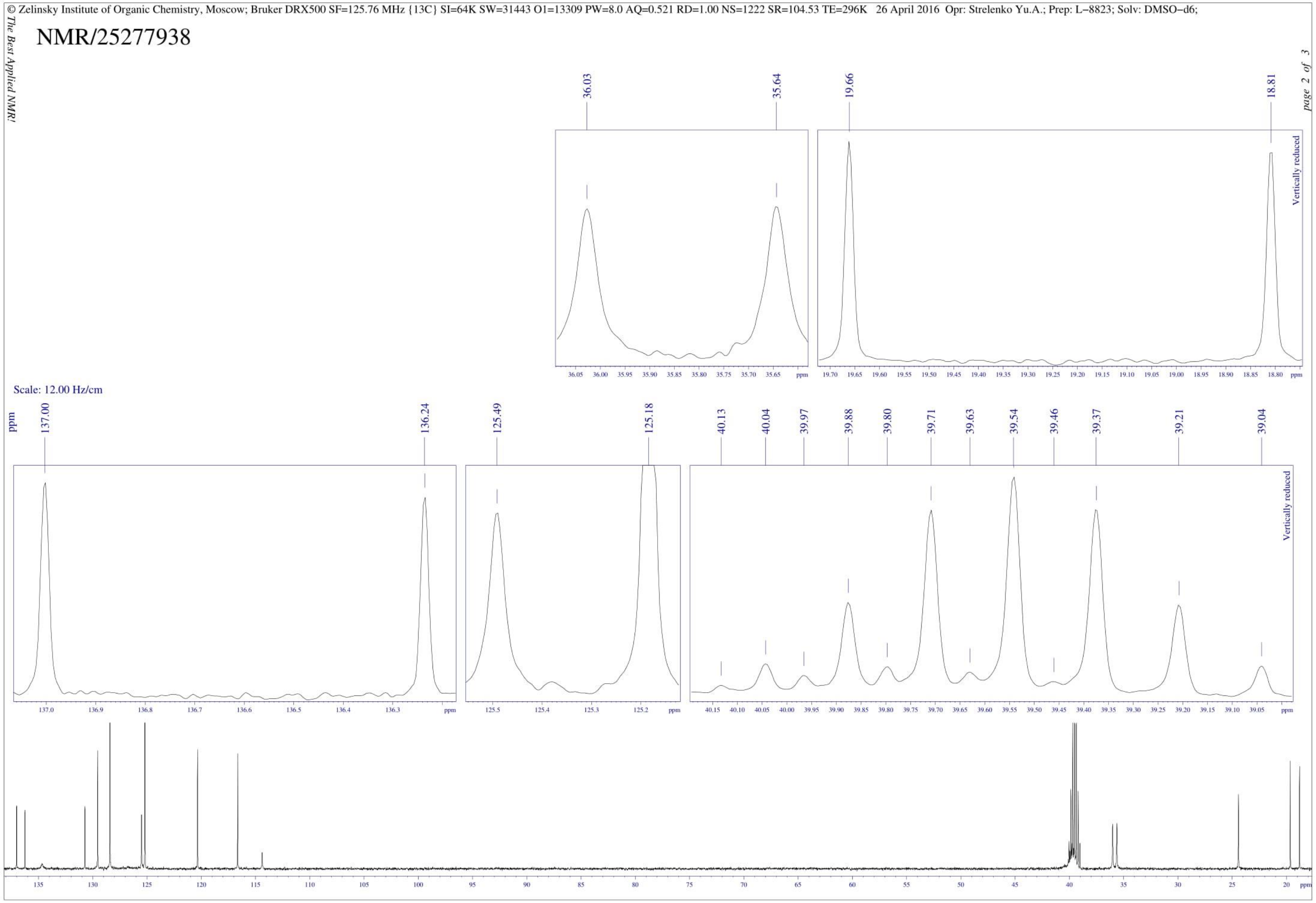
© Zelinsky Institute of Organic Chemistry, Moscow; Bruker DRX500 SF=125.76 MHz {13C} SI=64K SW=31443 O1=13309 PW=8.0 AQ=0.521 RD=1.00 NS=1222 SR=104.53 TE=296K 26 April 2016 Opr: Strelenko Yu.A.; Prep: L−8823; Solv: DMSO−d6;
The Best Applied NMR!
NMR/25277938
page 2 of 3
36.03
35.64
19.66
18.81
Vertically reduced
Scale: 12.00 Hz/cm
ppm
137.00
136.24
125.49
125.18
40.13
40.04
39.97
39.88
39.80
39.71
39.63
39.54
39.46
39.37
39.21
39.04
Vertically reduced

© Zelinsky Institute of Organic Chemistry, Moscow; Bruker DRX500 SF=125.76 MHz {13C} SI=64K SW=31443 O1=13309 PW=8.0 AQ=0.521 RD=1.00 NS=1222 SR=104.53 TE=296K 26 April 2016 Opr: Strelenko Yu.A.; Prep: L−8823; Solv: DMSO−d6;

The Best Applied NMR!

NMR/25277938

Peaks List

#	Address [points]	Frequency [Hz]	Frequency [ppm]	Intensity [cm]
1	15666.9	21410.324	170.2504	0.96
2	15991.3	21254.639	169.0124	4.02
3	22967.2	17907.342	142.3954	1.59
4	24380.6	17229.156	137.0026	4.60
5	24581.7	17132.670	136.2354	4.27
6	26029.2	16438.111	130.7124	4.51
7	26336.8	16290.480	129.5385	8.50
8	26632.8	16148.467	128.4092	12.68
9	27397.6	15781.492	125.4911	3.91
10	27478.1	15742.881	125.1841	14.00
11	28750.9	15132.152	120.3277	8.58
12	29719.1	14667.534	116.6332	8.30
13	30310.5	14383.797	114.3769	1.18
14	49768.8	5046.991	40.1326	0.70
15	49792.3	5035.684	40.0427	1.99
16	49812.6	5025.962	39.9654	1.29
17	49836.1	5014.673	39.8756	5.71
18	49856.7	5004.790	39.7970	1.83
19	49879.9	4993.654	39.7085	11.29
20	49900.5	4983.810	39.6302	1.50
21	49923.7	4972.650	39.5415	13.33
22	49945.0	4962.457	39.4604	0.93
23	49967.4	4951.676	39.3747	11.39
24	50011.2	4930.666	39.2076	5.57
25	50055.0	4909.666	39.0406	1.87
26	50844.8	4530.665	36.0269	3.23
27	50945.4	4482.397	35.6431	3.28
28	53882.5	3073.083	24.4365	5.42
29	55134.0	2472.548	19.6612	7.76
30	55357.5	2365.316	18.8085	7.53

**Data Filename**	LCMS_2686.d	**Sample Name**	#1545
**Sample Type**	Sample	**Position**	Vial 9
**Instrument Name**	Instrument 1	**User Name**	Falaleev [illegible]
**Acq Method**	ACN-H2O_40-60.m	**Acquired Time**	22-Dec-1[illegible]
**IRM Calibration Status**	Success	**DA Method**	alex2015[illegible]
**Comment**	4g / 2-(7-amino-2-oxo-5-phenyl-1,2,3,4-tetrahydroimidazo[1,5-b]pyridazin-3-yl)-N-(3,4-dimethylphenyl)acetamide		

**Stream Name**	LC 1	**Acquisition SW Version**	6200 series TOF/6500 series Q-TOF B.06.01 (B6157)

User Chromatograms

**Fragmentor Voltage**	191	**Collision Energy**	0	**Ionization Mode**	ESI

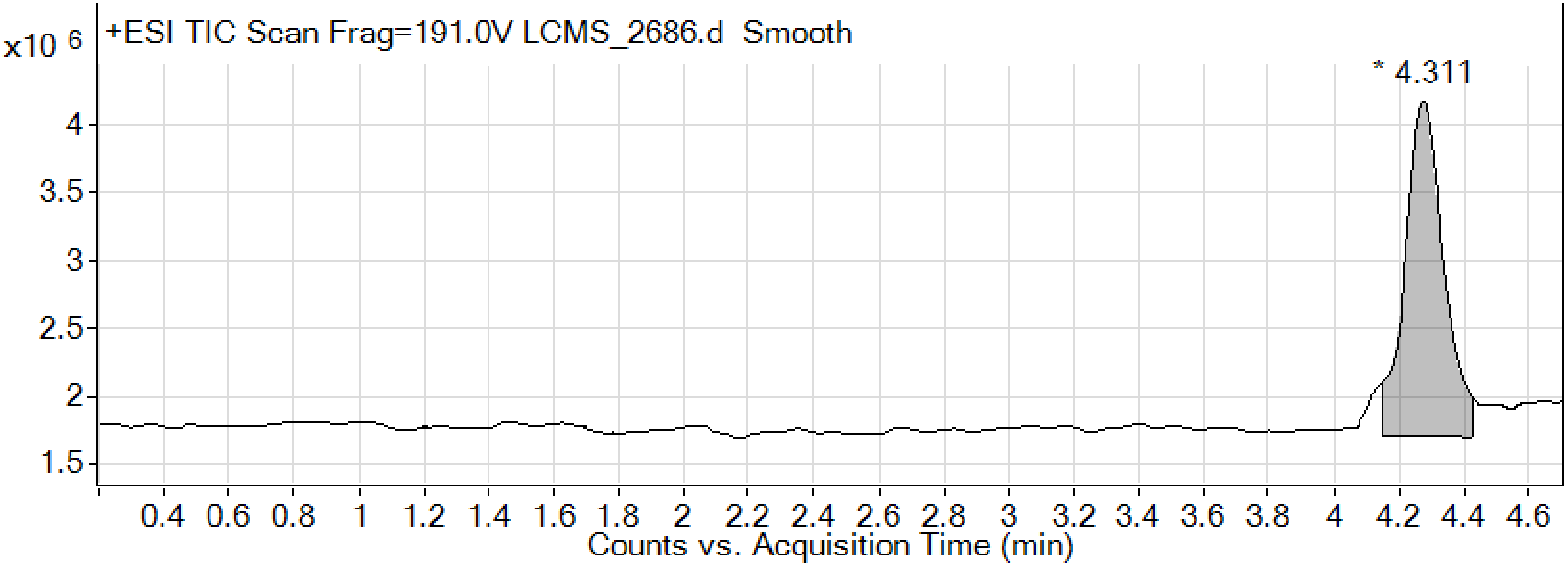

Integration Peak List

Peak	Start	RT	End	Height	Area	Area %
1	4,228	4,311	4,411	2485641,86	20368127,21	100

User Spectra

**Spectrum Source**	**Fragmentor Voltage**	**Collision Energy**	**Ionization Mode**
Peak (1) in "+ TIC Scan Smo"	191	0	ESI

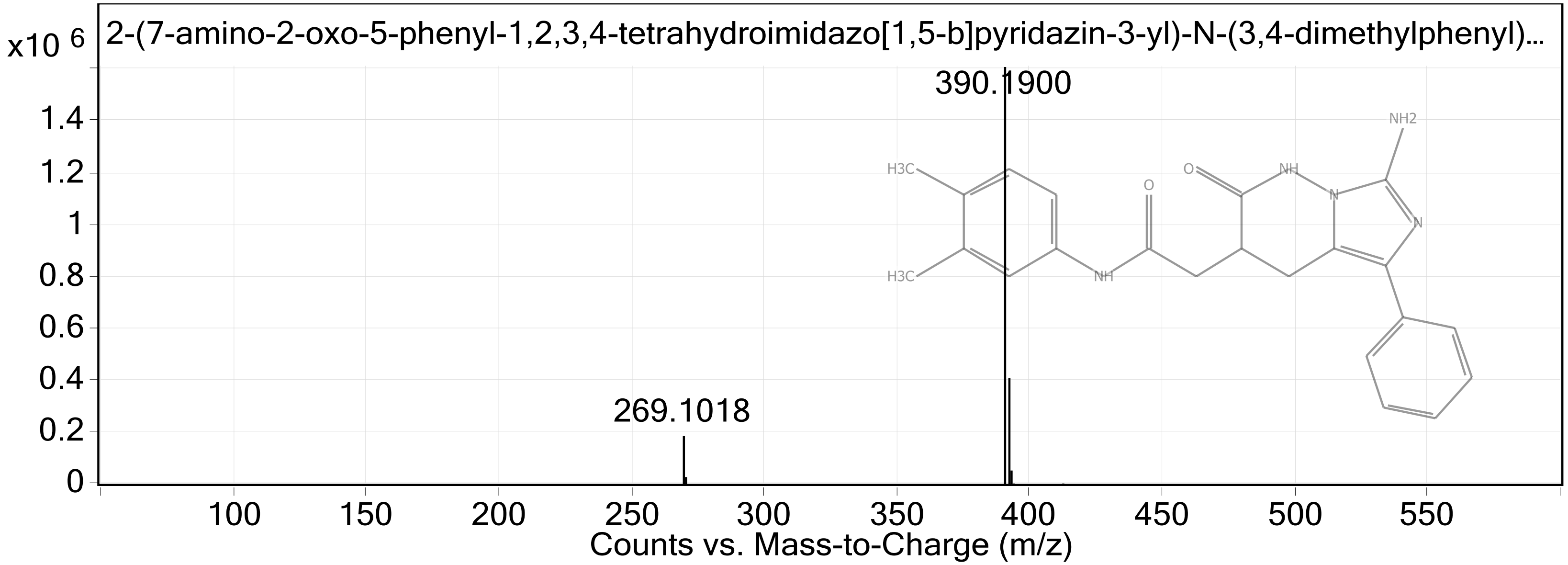

Peak List

m/z	z	Abund
269,1018		195154,27
390,19	1	1611606,88
391,1936	1	415030,44

Spectrum Structure

2-(7-amino-2-oxo-5-phenyl-1,2,3,4-tetrahydroimidazo[1,5-b]pyridazin-3-yl)-N-(3,4-dimethylphenyl)acetamide

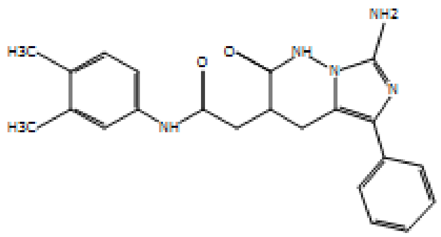

--- End Of Report ---

2-(7-Amino-2-oxo-5-phenyl-1,2,3,4-tetrahydroimidazo[1,5-*b*]pyridazin-3-yl)-*N*-(3,5-dimethylphenyl)acetamide (9f)

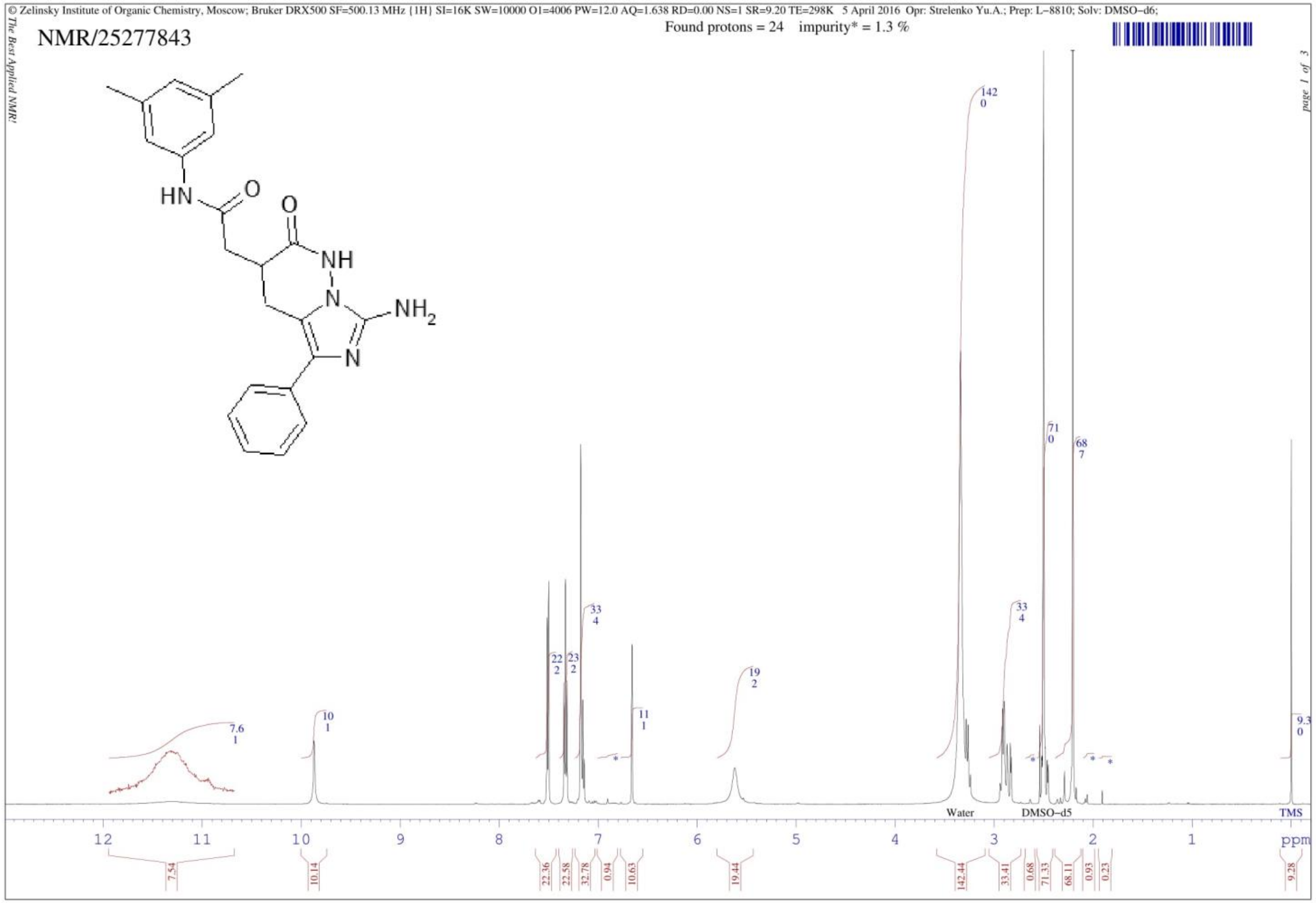

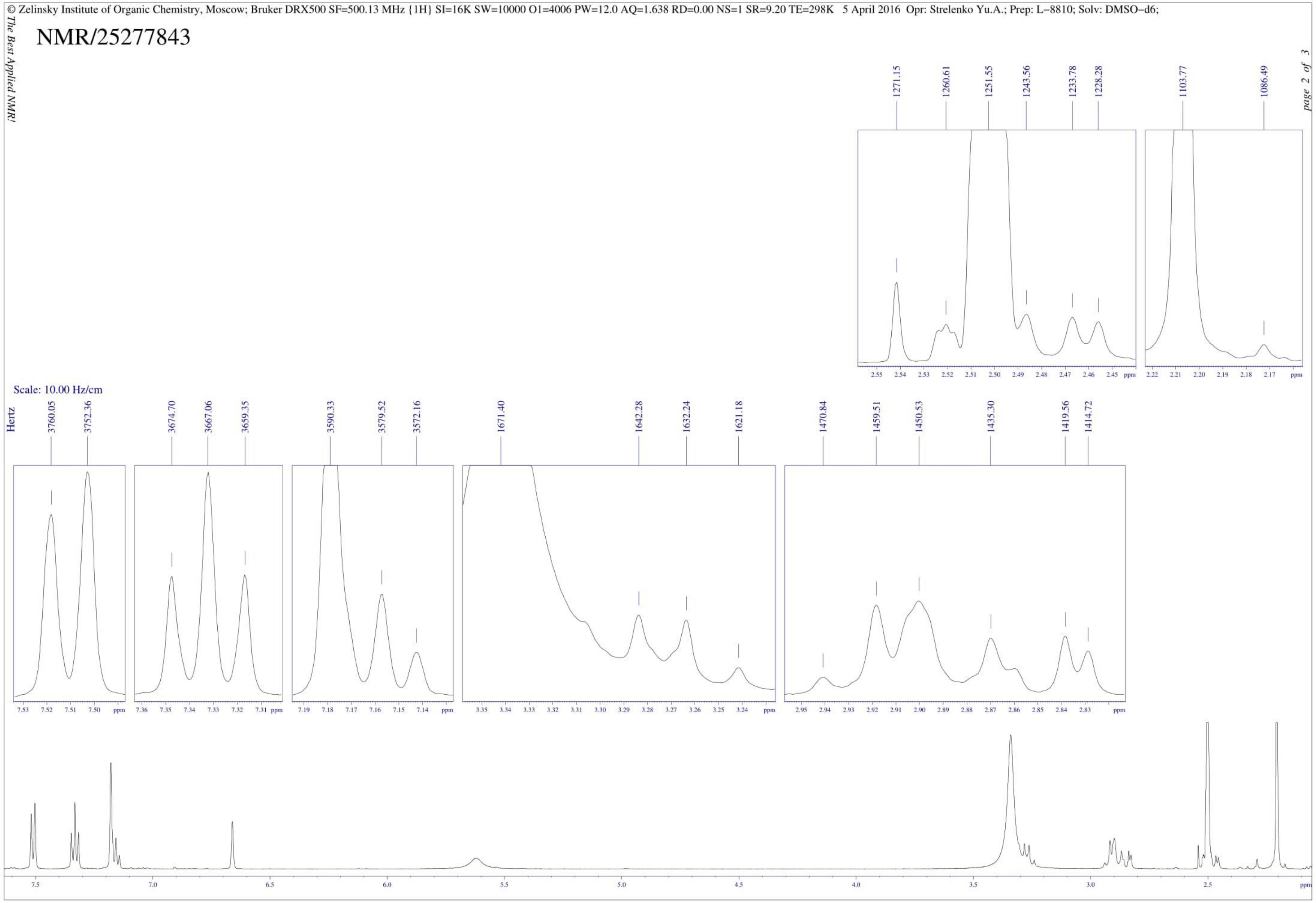
© Zelinsky Institute of Organic Chemistry, Moscow; Bruker DRX500 SF=500.13 MHz {1H} SI=16K SW=10000 O1=4006 PW=12.0 AQ=1.638 RD=0.00 NS=1 SR=9.20 TE=298K 5 April 2016 Opr: Strelenko Yu.A.; Prep: L–8810; Solv: DMSO–d6;
The Best Applied NMR!
NMR/25277843
page 2 of 3
Scale: 10.00 Hz/cm
Hertz
3760.05 3752.36 3674.70 3667.06 3659.35 3590.33 3579.52 3572.16 1671.40 1642.28 1632.24 1621.18 1470.84 1459.51 1450.53 1435.30 1419.56 1414.72
1271.15 1260.61 1251.55 1243.56 1233.78 1228.28 1103.77 1086.49
ppm

The Best Applied NMR!

NMR/25277843

Peaks List

#	Address [points]	Frequency [Hz]	Frequency [ppm]	Intensity [cm]
1	13302.7	4936.928	9.8713	1.25
2	17159.1	3760.046	7.5181	3.79
3	17184.3	3752.363	7.5028	4.68
4	17438.7	3674.704	7.3475	2.51
5	17463.8	3667.063	7.3322	4.68
6	17489.1	3659.348	7.3168	2.54
7	17715.2	3590.334	7.1788	7.36
8	17750.6	3579.516	7.1572	2.14
9	17774.7	3572.163	7.1425	0.93
10	18564.5	3331.144	6.6606	3.28
11	20266.7	2811.664	5.6219	0.69
12	24003.2	1671.402	3.3419	8.56
13	24098.6	1642.281	3.2837	1.71
14	24131.5	1632.245	3.2636	1.60
15	24167.7	1621.182	3.2415	0.61
16	24660.4	1470.841	2.9409	0.41
17	24697.5	1459.513	2.9183	1.91
18	24726.9	1450.534	2.9003	2.00
19	24776.8	1435.301	2.8699	1.22
20	24828.4	1419.562	2.8384	1.27
21	24844.3	1414.719	2.8287	0.95
22	25314.7	1271.148	2.5416	1.66
23	25349.2	1260.607	2.5206	0.77
24	25378.9	1251.552	2.5025	15.21
25	25405.1	1243.555	2.4865	0.99
26	25437.2	1233.779	2.4669	0.92
27	25455.2	1228.279	2.4559	0.83
28	25725.0	1145.926	2.2913	0.70
29	25863.2	1103.770	2.2070	31.46
30	25919.8	1086.493	2.1724	0.35
31	26101.9	1030.918	2.0613	0.20
32	26349.3	955.417	1.9103	0.30
33	29480.0	0.001	0.0000	7.55

© Zelinsky Institute of Organic Chemistry, Moscow; Bruker DRX500 SF=125.76 MHz {13C} SI=64K SW=31443 O1=13309 PW=8.0 AQ=0.521 RD=1.00 NS=627 SR=122.95 TE=296K 21 April 2016 Opr: Strelenko Yu.A.; Prep: L–8819; Solv: DMSO–d6;

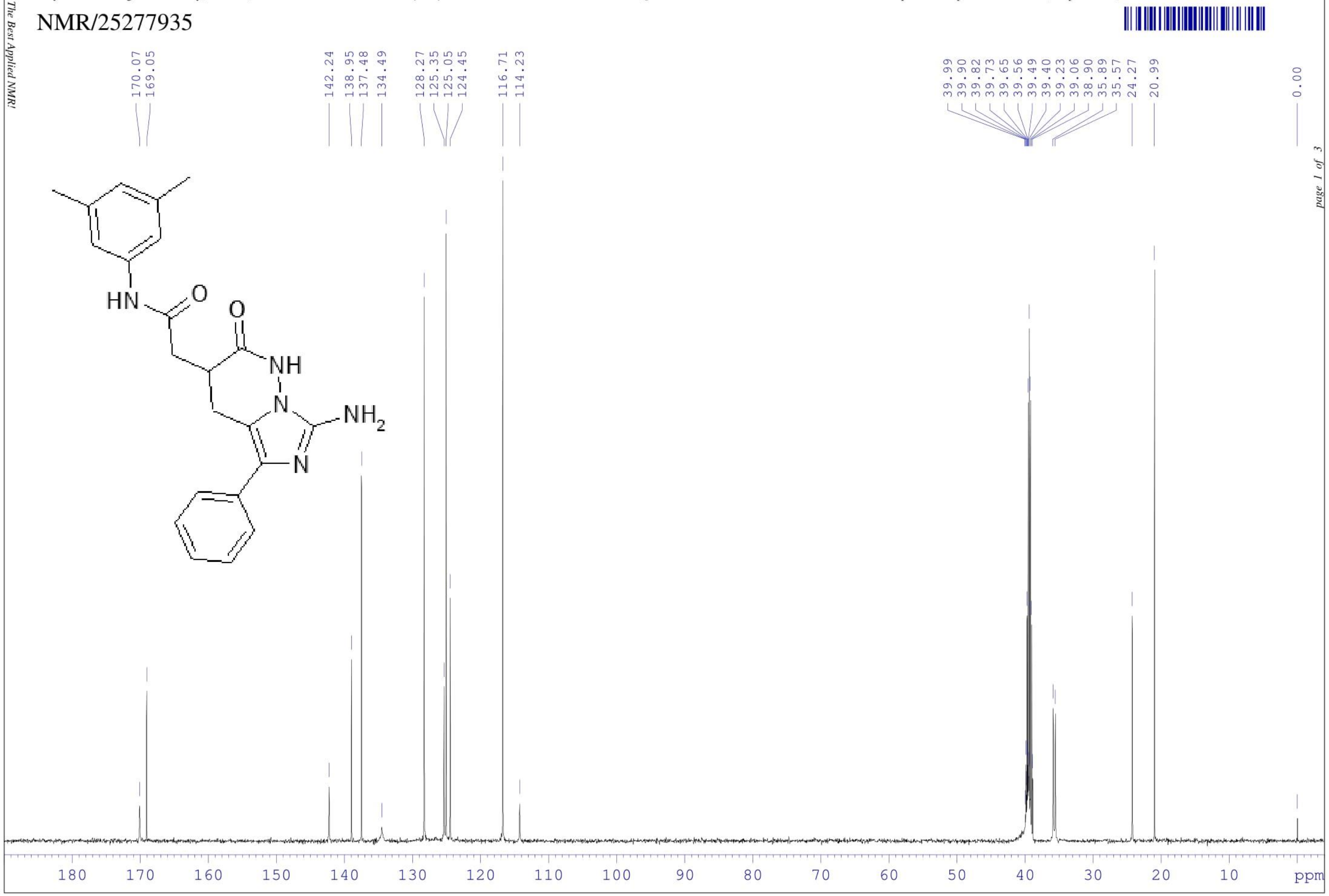

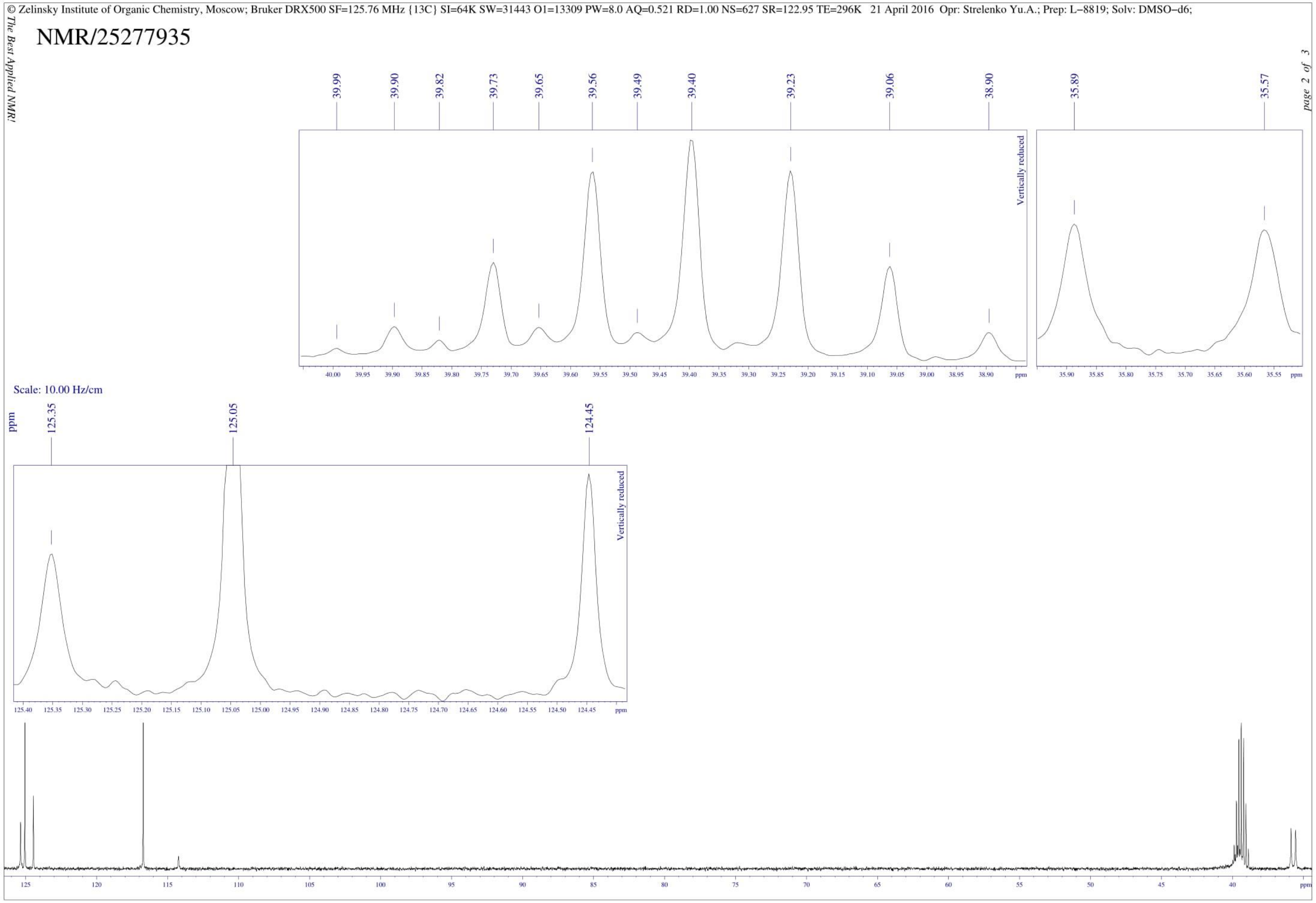
© Zelinsky Institute of Organic Chemistry, Moscow; Bruker DRX500 SF=125.76 MHz {13C} SI=64K SW=31443 O1=13309 PW=8.0 AQ=0.521 RD=1.00 NS=627 SR=122.95 TE=296K 21 April 2016 Opr: Strelenko Yu.A.; Prep: L–8819; Solv: DMSO–d6;
The Best Applied NMR!
NMR/25277935
page 2 of 3
39.99
39.90
39.82
39.73
39.65
39.56
39.49
39.40
39.23
39.06
38.90
35.89
35.57
Vertically reduced
Scale: 10.00 Hz/cm
ppm
125.35
125.05
124.45
Vertically reduced
ppm

© Zelinsky Institute of Organic Chemistry, Moscow; Bruker DRX500 SF=125.76 MHz {13C} SI=64K SW=31443 O1=13309 PW=8.0 AQ=0.521 RD=1.00 NS=627 SR=122.95 TE=296K 21 April 2016 Opr: Strelenko Yu.A.; Prep: L–8819; Solv: DMSO–d6;

The Best Applied NMR!

NMR/25277935

Peaks List

#	Address [points]	Frequency [Hz]	Frequency [ppm]	Intensity [cm]
1	15674.5	21388.242	170.0748	0.82
2	15942.8	21259.469	169.0508	3.26
3	22970.5	17887.365	142.2366	1.22
4	23831.3	17474.287	138.9518	3.95
5	24217.3	17289.088	137.4792	7.78
6	25001.1	16912.986	134.4885	0.39
7	26632.0	16130.405	128.2656	11.42
8	27395.6	15764.028	125.3522	3.33
9	27475.8	15725.532	125.0461	12.76
10	27633.1	15650.080	124.4461	5.18
11	29659.4	14677.758	116.7144	14.00
12	30311.1	14365.062	114.2279	0.88
13	49766.8	5029.535	39.9938	0.64
14	49792.2	5017.312	39.8966	1.70
15	49812.1	5007.779	39.8208	1.04
16	49835.9	4996.343	39.7299	4.81
17	49856.0	4986.687	39.6531	1.66
18	49879.6	4975.362	39.5630	9.27
19	49899.5	4965.834	39.4873	1.42
20	49923.4	4954.348	39.3959	10.84
21	49967.2	4933.363	39.2291	9.27
22	50010.9	4912.387	39.0623	4.62
23	50054.7	4891.360	38.8951	1.42
24	50843.1	4513.062	35.8869	2.87
25	50927.2	4472.711	35.5661	2.75
26	53888.5	3051.757	24.2669	4.83
27	54746.4	2640.121	20.9937	12.15
28	60248.5	0.003	0.0000	0.60

**Data Filename**	LCMS_2683.d	**Sample Name**	#1542
**Sample Type**	Sample	**Position**	Vial 6
**Instrument Name**	Instrument 1	**User Name**	Falaleev [illegible]
**Acq Method**	ACN-H2O_40-60.m	**Acquired Time**	22-Dec-1[illegible]
**IRM Calibration Status**	Success	**DA Method**	alex2015[illegible]
**Comment**	4d / 2-(7-amino-2-oxo-5-phenyl-1,2,3,4-tetrahydroimidazo[1,5-b]pyridazin-3-yl)-N-(3,5-dimethylphenyl)acetamide		

**Stream Name**	LC 1	**Acquisition SW Version**	6200 series TOF/6500 series Q-TOF B.06.01 (B6157)

User Chromatograms

**Fragmentor Voltage**	191	**Collision Energy**	0	**Ionization Mode**	ESI

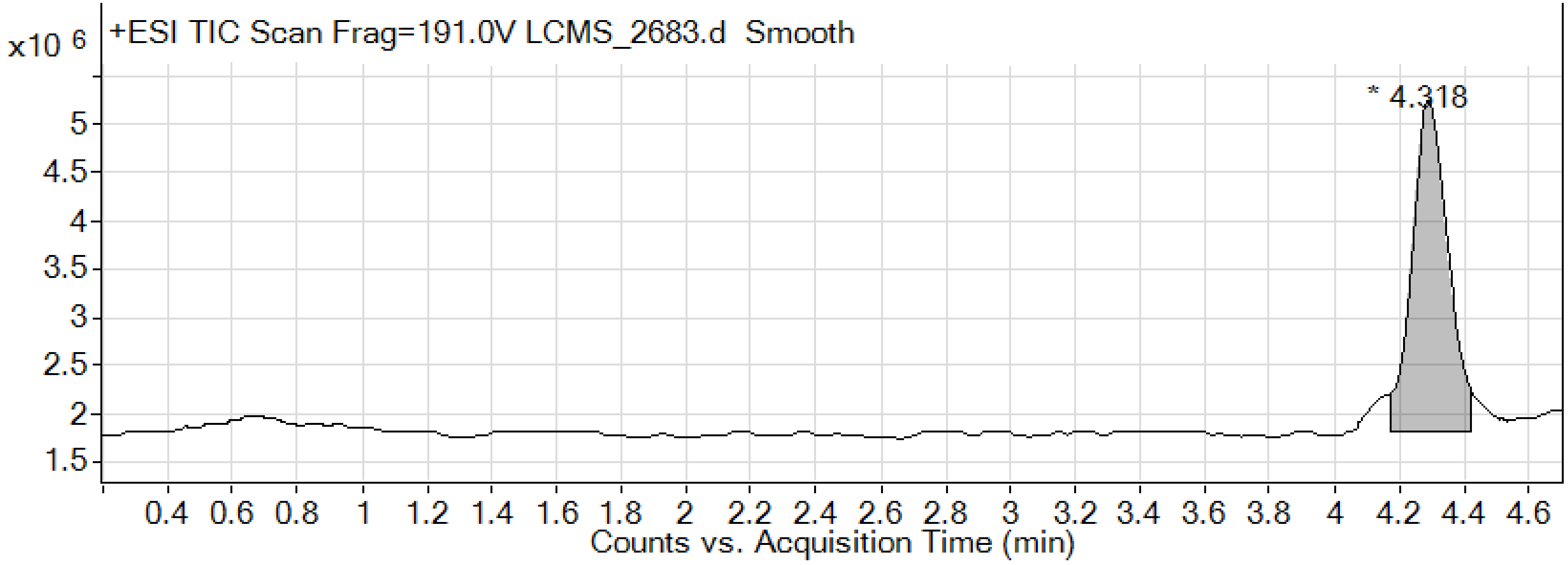

Integration Peak List

Peak	Start	RT	End	Height	Area	Area %
1	4,155	0,68	4,318	3445578,77	26585954,87	100

User Spectra

Spectrum Source	Fragmentor Voltage	Collision Energy	Ionization Mode
Peak (1) in "+ TIC Scan Smo"	191	0	ESI

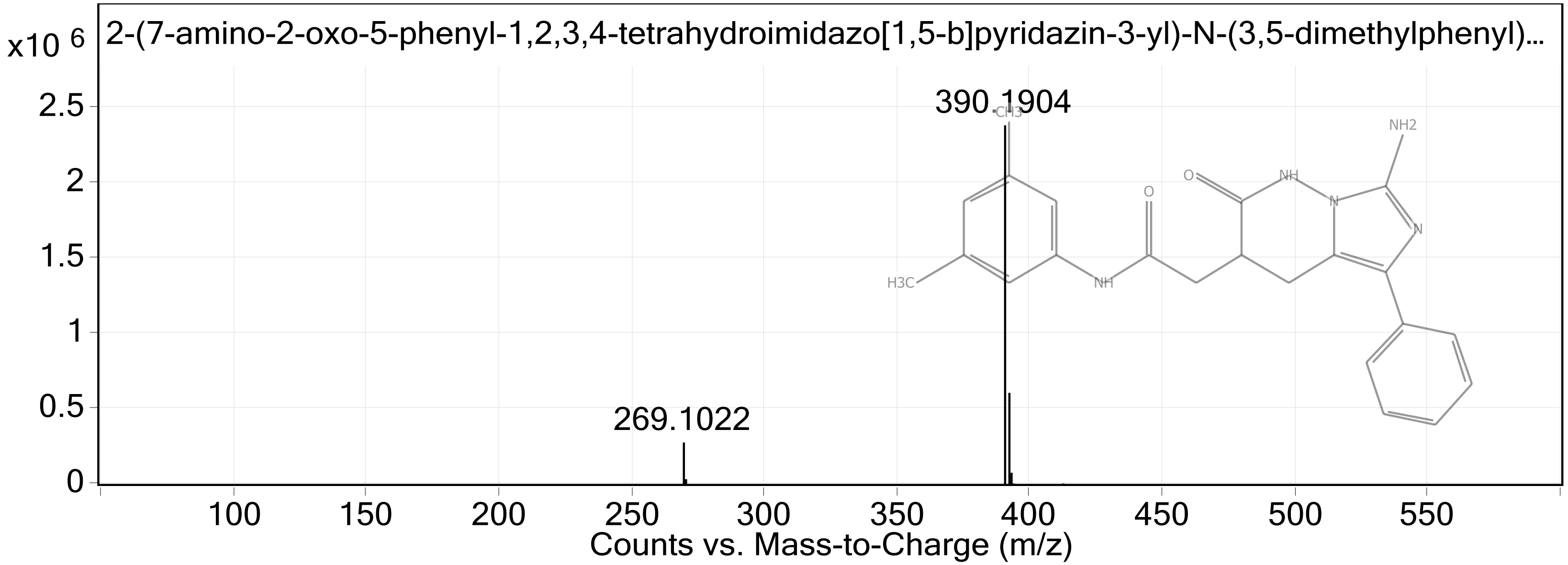

Peak List

m/z	z	Abund
269,1022		280521,47
390,1904	1	2394128
391,194	1	613890,94

Spectrum Structure

2-(7-amino-2-oxo-5-phenyl-1,2,3,4-tetrahydroimidazo[1,5-b]pyridazin-3-yl)-N-(3,5-dimethylphenyl)acetamide

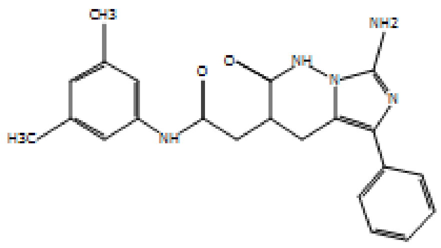

--- End Of Report ---

2-(7-Amino-2-oxo-5-phenyl-1,2,3,4-tetrahydroimidazo[1,5-*b*]pyridazin-3-yl)-*N*-(3,4-dichlorophenyl)acetamide (9g)

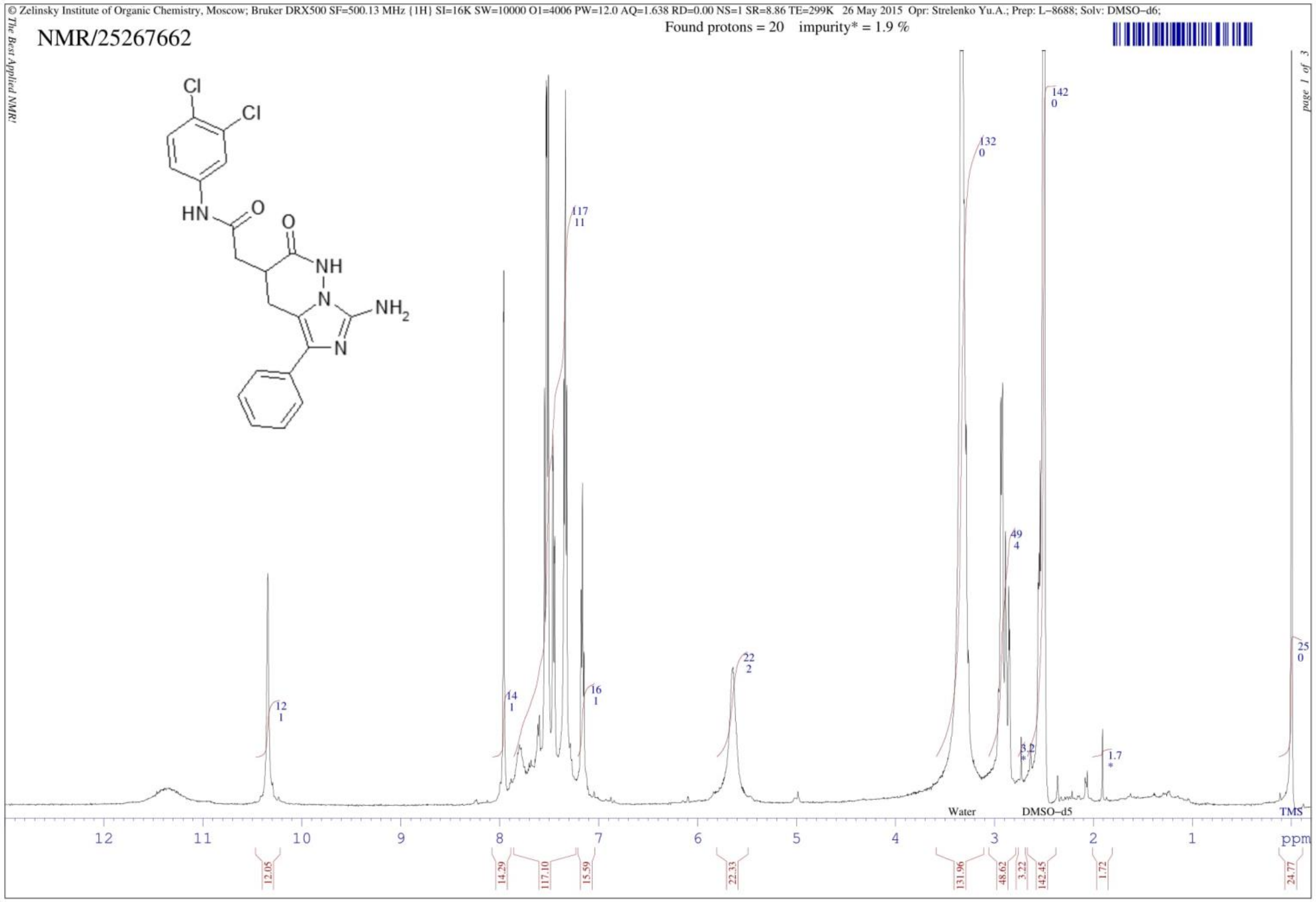

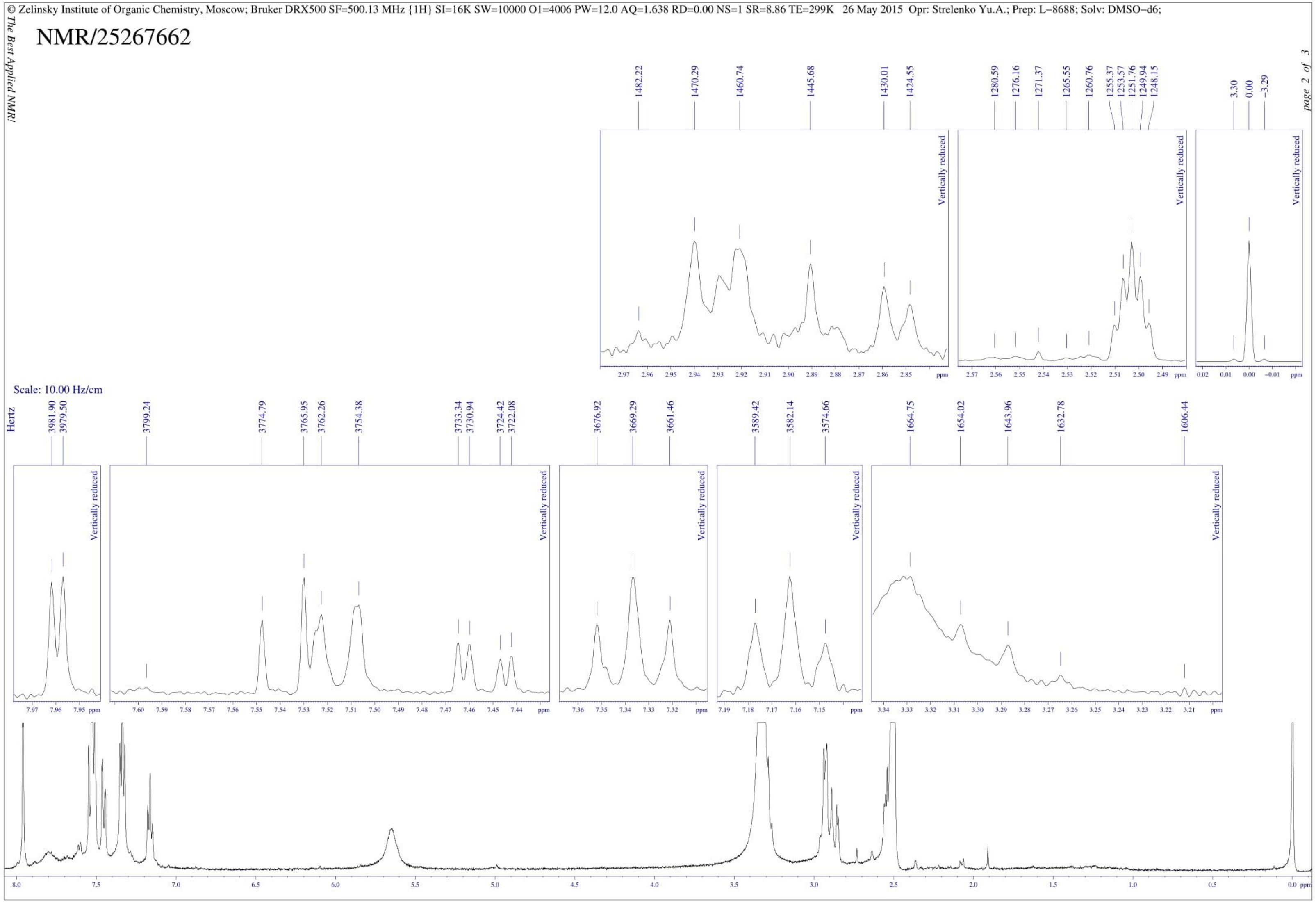
© Zelinsky Institute of Organic Chemistry, Moscow; Bruker DRX500 SF=500.13 MHz {1H} SI=16K SW=10000 O1=4006 PW=12.0 AQ=1.638 RD=0.00 NS=1 SR=8.86 TE=299K 26 May 2015 Opr: Strelenko Yu.A.; Prep: L–8688; Solv: DMSO–d6;
The Best Applied NMR!
NMR/25267662
page 2 of 3
Scale: 10.00 Hz/cm
Hertz
Vertically reduced

© Zelinsky Institute of Organic Chemistry, Moscow; Bruker DRX500 SF=500.13 MHz {1H} SI=16K SW=10000 O1=4006 PW=12.0 AQ=1.638 RD=0.00 NS=1 SR=8.86 TE=299K 26 May 2015 Opr: Strelenko Yu.A.; Prep: L–8688; Solv: DMSO–d6;

The Best Applied NMR!

NMR/25267662

Peaks List

#	Address [points]	Frequency [Hz]	Frequency [ppm]	Intensity [cm]
1	12529.7	5173.142	10.3436	3.63
2	16433.1	3981.896	7.9617	12.65
3	16441.0	3979.500	7.9569	13.28
4	17031.6	3799.245	7.5965	1.53
5	17111.8	3774.787	7.5476	12.33
6	17140.7	3765.953	7.5299	19.24
7	17152.8	3762.258	7.5226	13.20
8	17178.7	3754.377	7.5068	14.71
9	17247.6	3733.343	7.4647	8.80
10	17255.5	3730.940	7.4599	8.55
11	17276.8	3724.421	7.4469	6.07
12	17284.5	3722.076	7.4422	6.63
13	17432.5	3676.919	7.3519	9.29
14	17457.5	3669.286	7.3367	15.32
15	17483.1	3661.460	7.3210	9.77
16	17719.2	3589.416	7.1770	4.24
17	17743.1	3582.136	7.1624	6.86
18	17767.6	3574.655	7.1475	3.09
19	20232.2	2822.514	5.6436	2.15
20	24025.9	1664.753	3.3286	15.35
21	24061.1	1654.015	3.3072	9.24
22	24094.1	1643.964	3.2871	6.61
23	24130.7	1632.783	3.2647	2.76
24	24217.0	1606.444	3.2121	1.17
25	24624.1	1482.222	2.9637	1.87
26	24663.2	1470.291	2.9398	7.20
27	24694.5	1460.740	2.9207	6.74
28	24743.8	1445.681	2.8906	5.85
29	24795.1	1430.011	2.8593	4.49
30	24813.1	1424.545	2.8483	3.42
31	25003.9	1366.292	2.7319	1.93
32	25284.8	1280.590	2.5605	3.31
33	25299.3	1276.162	2.5517	4.15
34	25315.0	1271.369	2.5421	8.24
35	25334.1	1265.549	2.5304	3.12
36	25349.7	1260.759	2.5209	5.53
37	25367.4	1255.372	2.5101	29.96
38	25373.3	1253.566	2.5065	68.18
39	25379.2	1251.756	2.5029	97.64
40	25385.2	1249.945	2.4992	69.21
41	25391.1	1248.151	2.4957	31.25
42	26349.4	955.694	1.9109	2.91
43	29470.2	3.296	0.0066	1.68
44	29481.0	0.001	0.0000	82.73
45	29491.8	-3.295	-0.0066	1.68

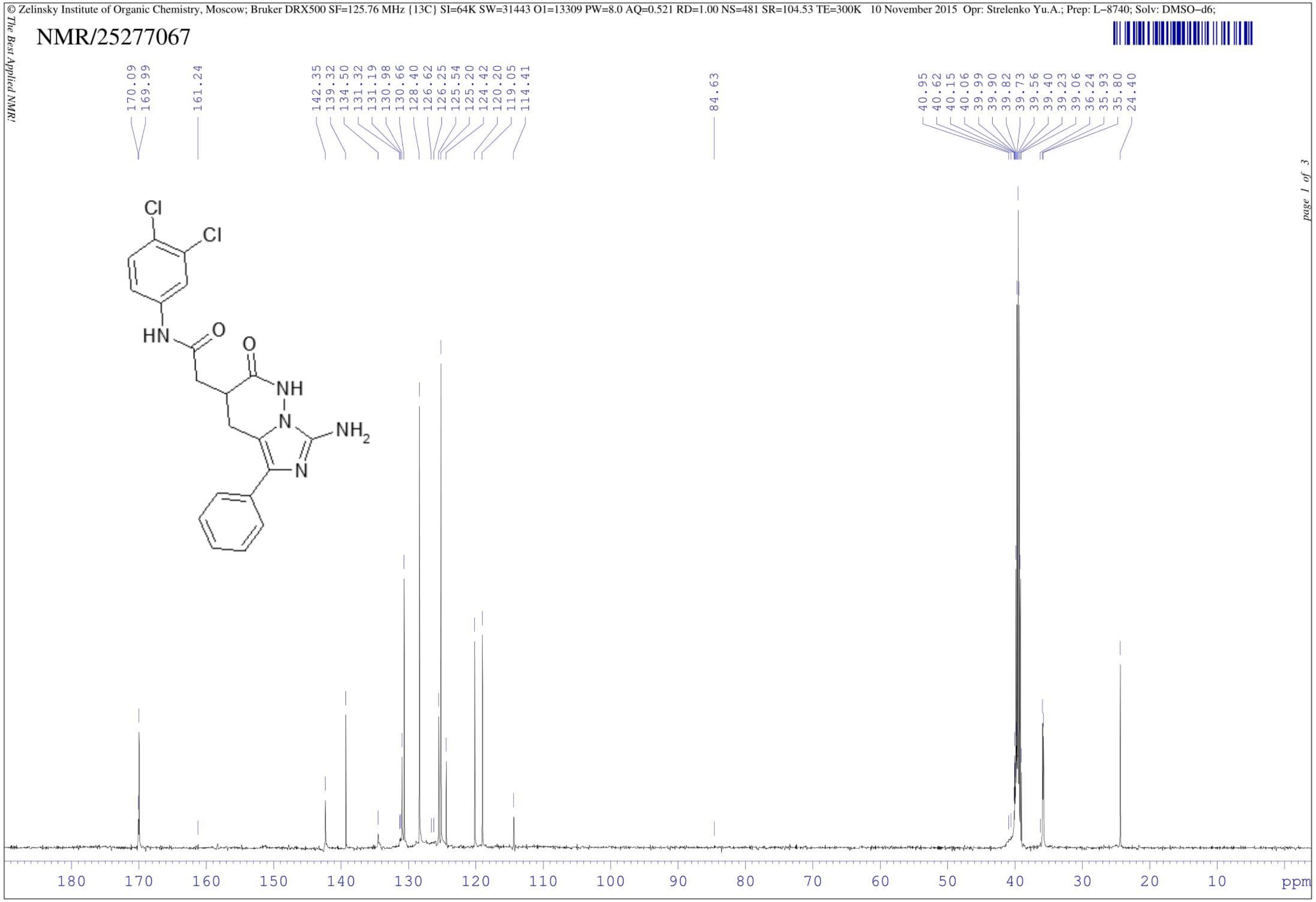

© Zelinsky Institute of Organic Chemistry, Moscow; Bruker DRX500 SF=125.76 MHz {13C} SI=64K SW=31443 O1=13309 PW=8.0 AQ=0.521 RD=1.00 NS=481 SR=104.53 TE=300K 10 November 2015 Opr: Strelenko Yu.A.; Prep: L–8740; Solv: DMSO–d6;
The Best Applied NMR!
NMR/25277067
page 1 of 3
170.09
169.99
161.24
142.35
139.32
134.50
131.32
131.19
130.98
130.66
128.40
126.62
126.25
125.54
125.20
124.42
120.20
119.05
114.41
84.63
40.95
40.62
40.15
40.06
39.99
39.90
39.82
39.73
39.56
39.40
39.23
39.06
36.24
35.93
35.80
24.40
Cl
Cl
HN
O
O
NH
N
NH_2
N
180
170
160
150
140
130
120
110
100
90
80
70
60
50
40
30
20
10
ppm

© Zelinsky Institute of Organic Chemistry, Moscow; Bruker DRX500 SF=125.76 MHz {13C} SI=64K SW=31443 O1=13309 PW=8.0 AQ=0.521 RD=1.00 NS=481 SR=104.53 TE=300K 10 November 2015 Opr: Strelenko Yu.A.; Prep: L–8740; Solv: DMSO–d6;

NMR/25277067

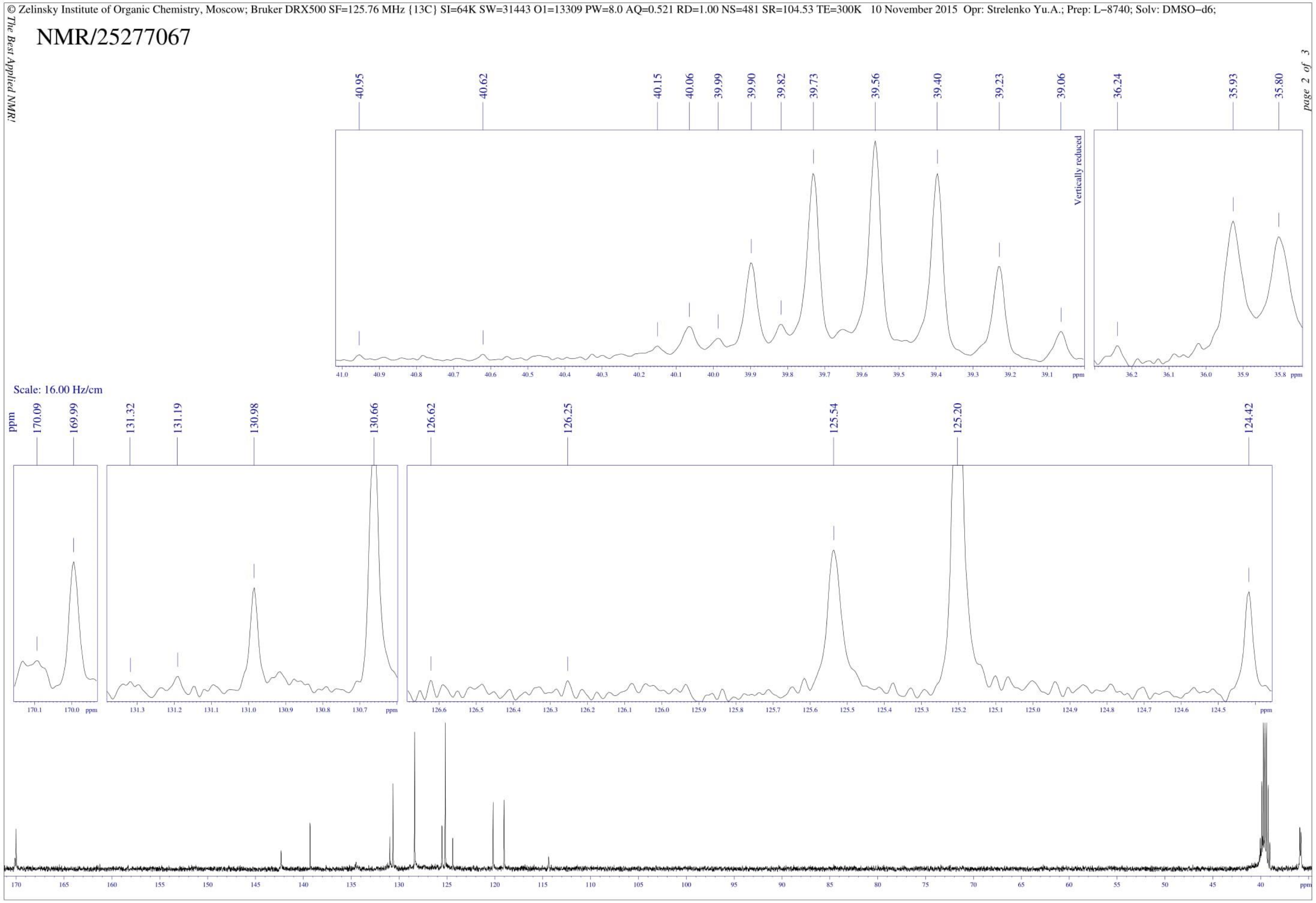

The Best Applied NMR!

NMR/25277067

Peaks List

#	Address [points]	Frequency [Hz]	Frequency [ppm]	Intensity [cm]
1	15708.0	21390.594	170.0935	0.77
2	15733.8	21378.197	169.9949	2.86
3	18028.1	20277.318	161.2410	0.35
4	22979.0	17901.686	142.3504	1.29
5	23774.0	17520.230	139.3172	3.29
6	25035.2	16915.035	134.5048	0.50
7	25870.3	16514.354	131.3187	0.33
8	25903.7	16498.312	131.1911	0.44
9	25957.8	16472.379	130.9849	2.32
10	26042.4	16431.787	130.6621	6.18
11	26635.3	16147.285	128.3998	9.87
12	27101.2	15923.698	126.6219	0.36
13	27197.9	15877.311	126.2530	0.34
14	27385.7	15787.200	125.5365	3.12
15	27473.1	15745.248	125.2029	10.76
16	27678.8	15646.563	124.4182	2.23
17	28784.4	15116.058	120.1997	4.81
18	29084.9	14971.841	119.0529	4.96
19	30300.7	14388.465	114.4141	0.87
20	38107.3	10642.612	84.6278	0.33
21	49553.5	5150.309	40.9542	0.43
22	49640.9	5108.333	40.6204	0.47
23	49764.0	5049.283	40.1508	0.99
24	49786.7	5038.395	40.0643	2.23
25	49807.0	5028.665	39.9869	1.48
26	49830.3	5017.451	39.8977	6.28
27	49851.3	5007.417	39.8179	2.37
28	49874.3	4996.358	39.7300	11.98
29	49917.9	4975.428	39.5636	14.00
30	49961.8	4954.388	39.3963	11.96
31	50005.4	4933.433	39.2296	6.07
32	50049.0	4912.511	39.0633	1.91
33	50789.4	4557.280	36.2385	0.34
34	50871.0	4518.110	35.9271	2.97
35	50903.3	4502.586	35.8036	2.65
36	53891.9	3068.593	24.4008	4.32

**Data Filename**	LCMS_2684.d	**Sample Name**	#1543
**Sample Type**	Sample	**Position**	Vial 7
**Instrument Name**	Instrument 1	**User Name**	Falaleev [illegible]
**Acq Method**	ACN-H2O_40-60.m	**Acquired Time**	22-Dec-1[illegible]
**IRM Calibration Status**	Success	**DA Method**	alex2015[illegible]
**Comment**	4e / 2-(7-amino-2-oxo-5-phenyl-1,2,3,4-tetrahydroimidazo[1,5-b]pyridazin-3-yl)-N-(3,4-dichlorophenyl)acetamide		

**Stream Name**	LC 1	**Acquisition SW Version**	6200 series TOF/6500 series Q-TOF B.06.01 (B6157)

User Chromatograms

**Fragmentor Voltage**	191	**Collision Energy**	0	**Ionization Mode**	ESI

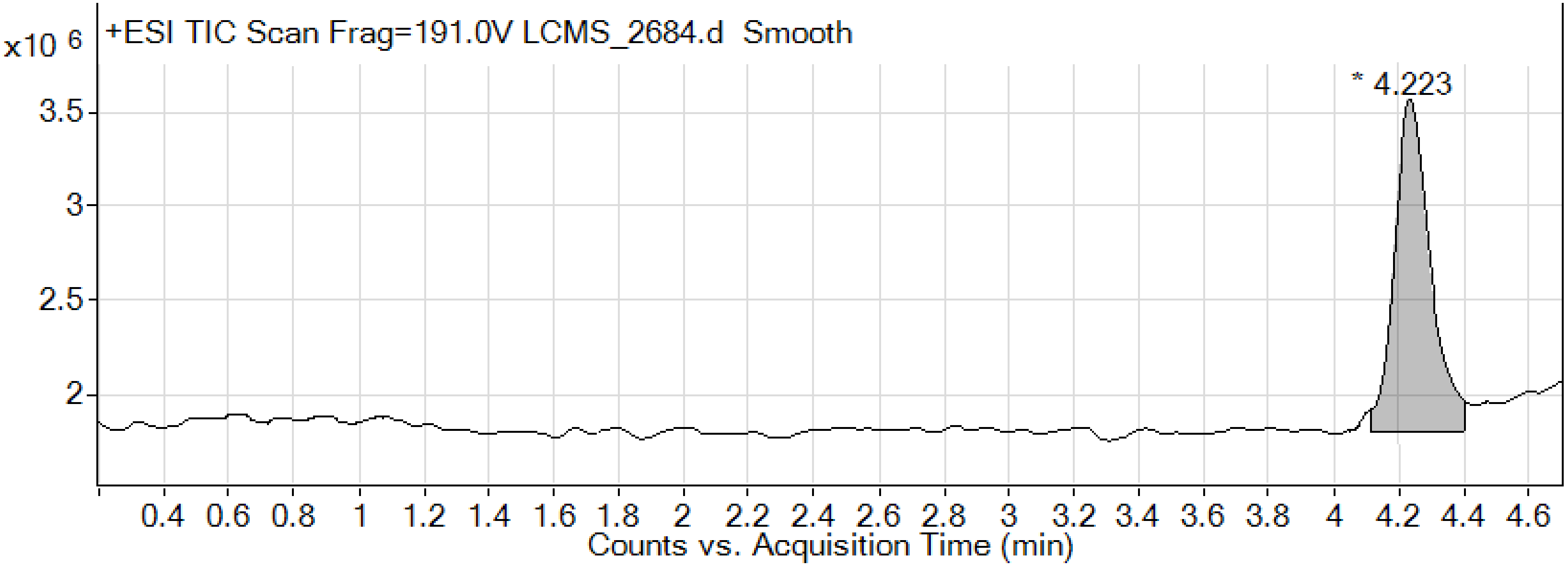

Integration Peak List

Peak	Start	RT	End	Height	Area	Area %
1	4,11	4,223	4,406	1765263,83	13623785,86	100

User Spectra

Spectrum Source	Fragmentor Voltage	Collision Energy	Ionization Mode
Peak (1) in "+ TIC Scan Smo"	191	0	ESI

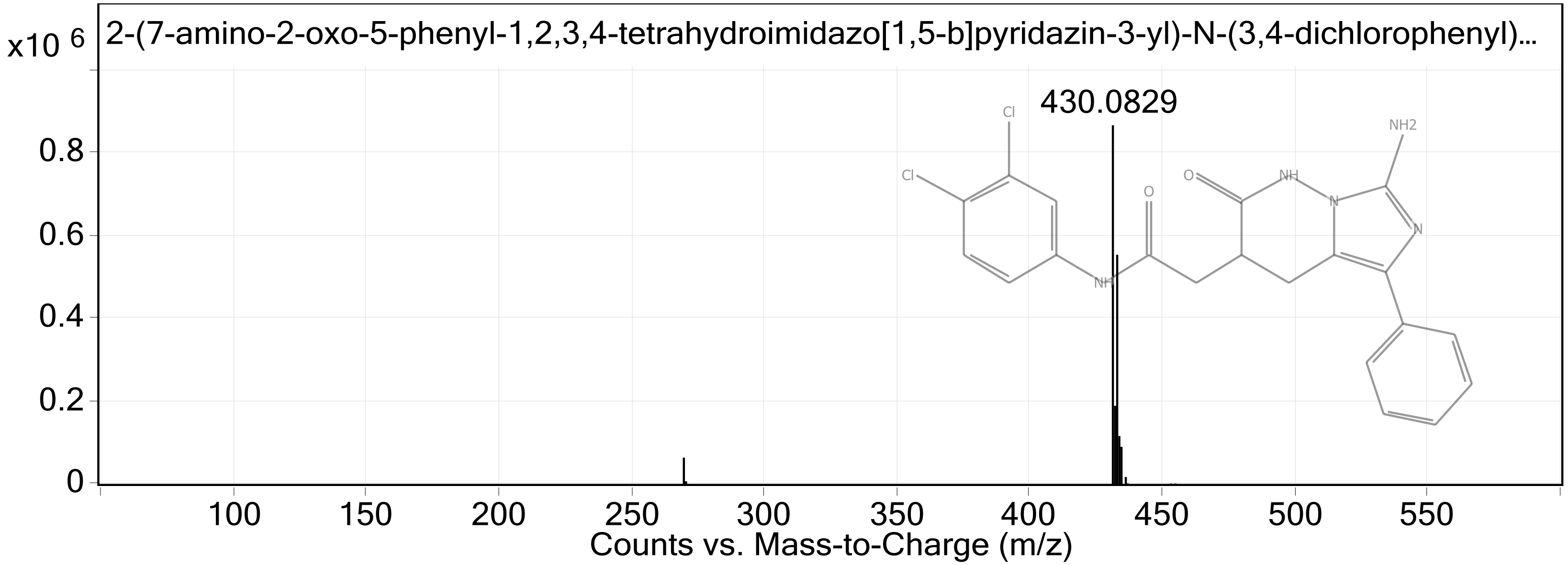

Peak List

m/z	z	Abund
430,0829	1	871094,75
431,0861	1	192983,55
432,0805	1	558106,81
433,083	1	121937,38
434,0785	1	93032,98

Spectrum Structure

2-(7-amino-2-oxo-5-phenyl-1,2,3,4-tetrahydroimidazo[1,5-b]pyridazin-3-yl)-N-(3,4-dichlorophenyl)acetamide

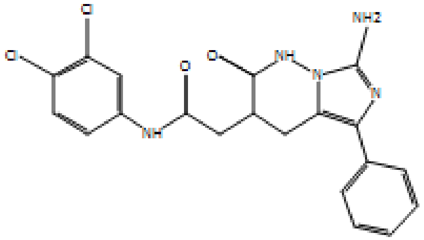

--- End Of Report ---

V. Results of HPLC–HRESIMS monitoring of the reaction mixture composition in the synthesis of imidazopyridazine 9d

Qualitative Analysis Report

**Data Filename**	LCMS_1212.d	**Sample Name**	#924
**Sample Type**	Sample	**Position**	Vial 5
**Instrument Name**	Instrument 1	**User Name**	Falaleev A.
**Acq Method**	MeOH-ACN-H2O 10-30-60.m	**Acquired Time**	19-Jan-16 4:41:15 PM
**IRM Calibration Status**	Success	**DA Method**	alex20150126.m
**Comment**	**Reaction MIX - 10 min+AcOH**		

Stream Name LC 1 **Acquisition SW Version** 6200 series TOF/6500 series Q-TOF B.06.01 (B6157)

User Chromatograms

Fragmentor Voltage 191 **Collision Energy** 0 **Ionization Mode** ESI

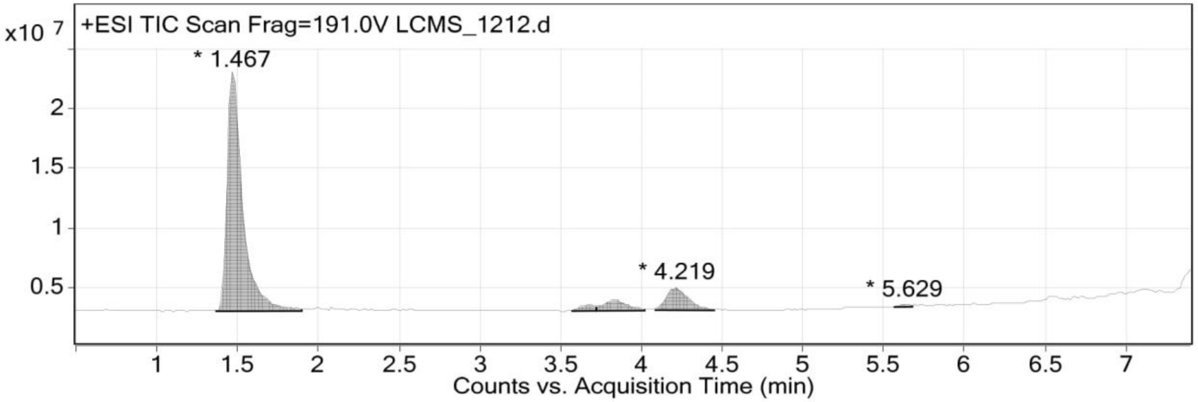

Integration Peak List

Peak	Start	RT	End	Height	Area	Area %	Area Sum, %
1	1,367	1,467	1,898	19996082	135528126	100	81,22
2	3,572	3,672	3,722	495779	3269886	2,41	1,96
3	3,722	3,821	4,02	868940	8946537	6,6	5,36

Agilent Technologies Page 1 of 6 Printed at: 16:36 on: 31.08.2017

4	4,086	4,219	4,451	1931747	17980329	13,27	10,78
5	5,562	5,629	5,678	222273	1133733	0,84	0,68

User Spectra

Spectrum Source	Fragmentor Voltage	Collision Energy	Ionization Mode
Peak (1) in "+ TIC Scan"	191	0	ESI

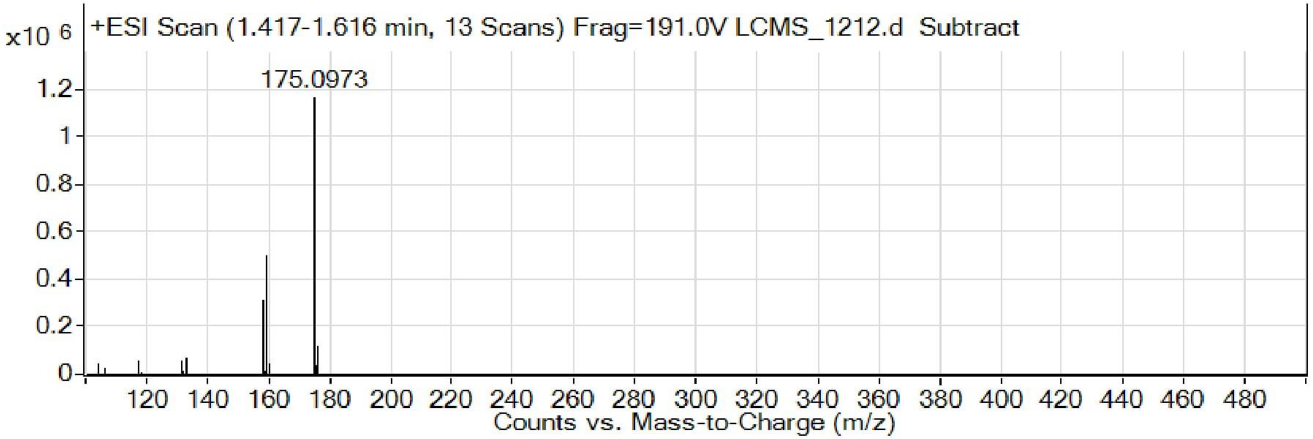

Peak List

m/z	z	Abund
158,0892		251037,8
159,0969		408416,8
175,0973	1	930683,67
176,0982	1	96357,12

Spectrum Source	Fragmentor Voltage	Collision Energy	Ionization Mode
Peak (2) in "+ TIC Scan"	191	0	ESI

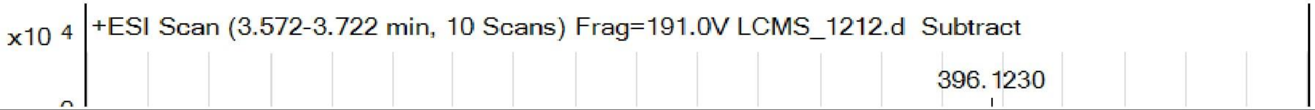

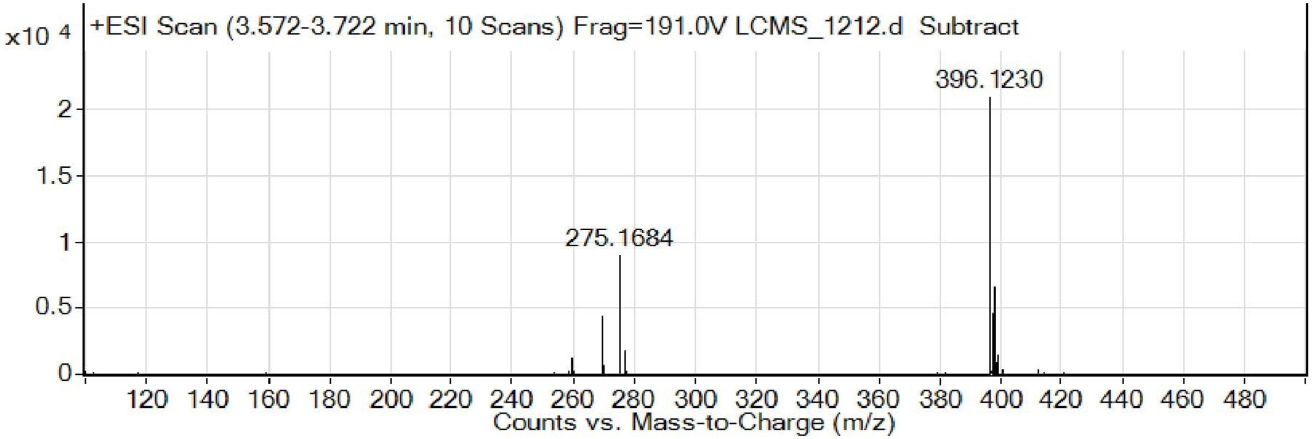

Peak List

m/z	z	Abund
269,1419		4388,76
275,1684		9105,06
396,1230	1	21036,62
397,1327	1	4670,68
398,1376	1	6831,51

Spectrum Source	Fragmentor Voltage	Collision Energy	Ionization Mode
Peak (3) in "+ TIC Scan"	191	0	ESI

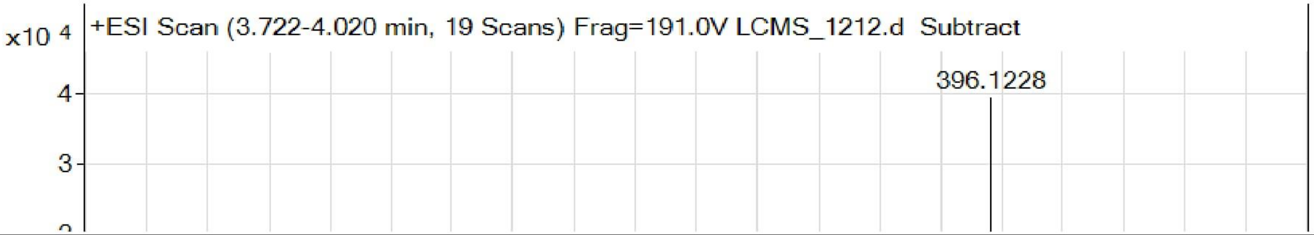

Qualitative Analysis Report

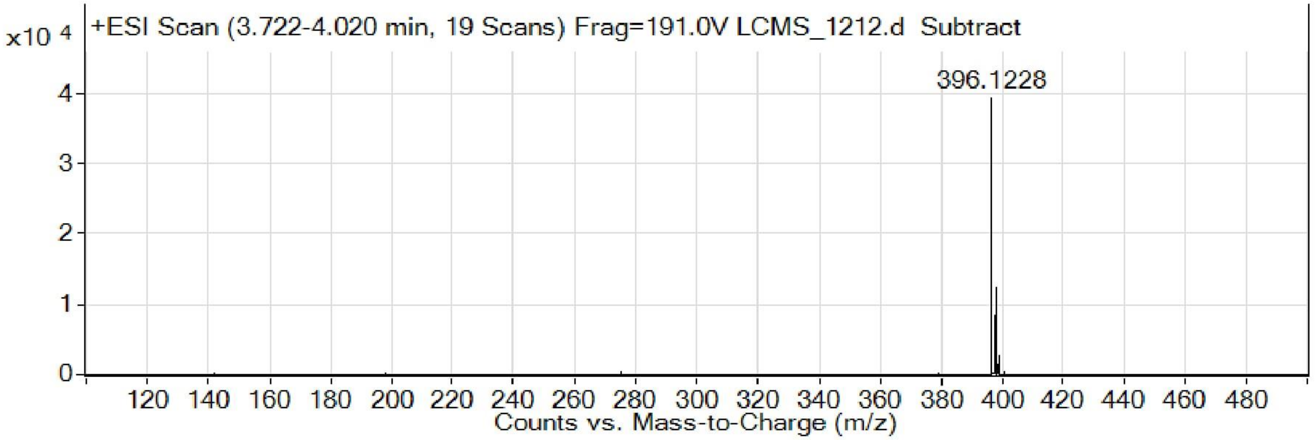

Peak List

m/z	z	Abund
396,1228	1	39528,66
397,1228	1	8628,43
398,1277	1	12626,5

Spectrum Source	Fragmentor Voltage	Collision Energy	Ionization Mode
Peak (4) in "+ TIC Scan"	191	0	ESI

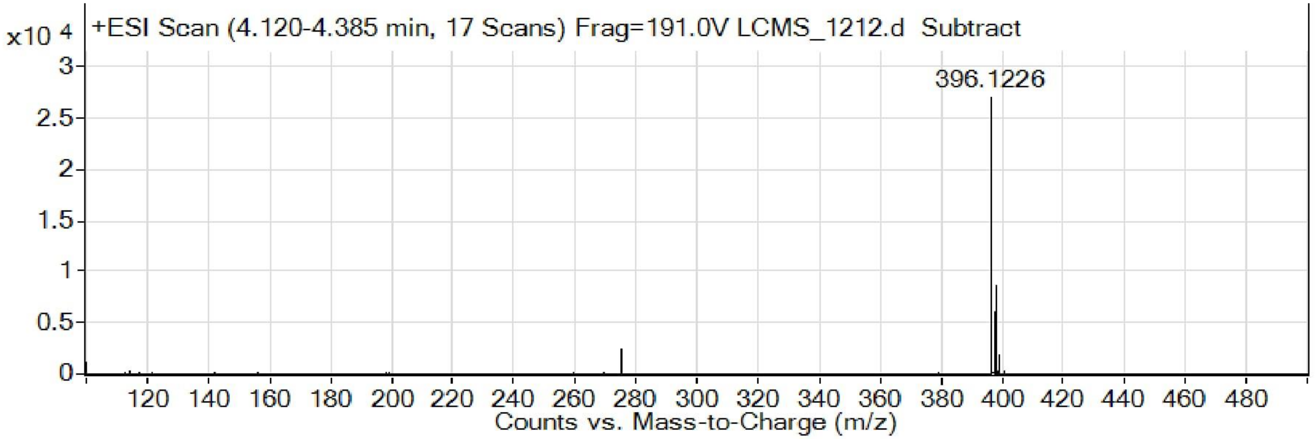

Peak List

m/z	z	Abund
396,1226	1	92666,25
397,1242	1	20681,49
398,1291	1	29812,99

Spectrum Source	Fragmentor Voltage	Collision Energy	Ionization Mode
Peak (5) in "+ TIC Scan"	191	0	ESI

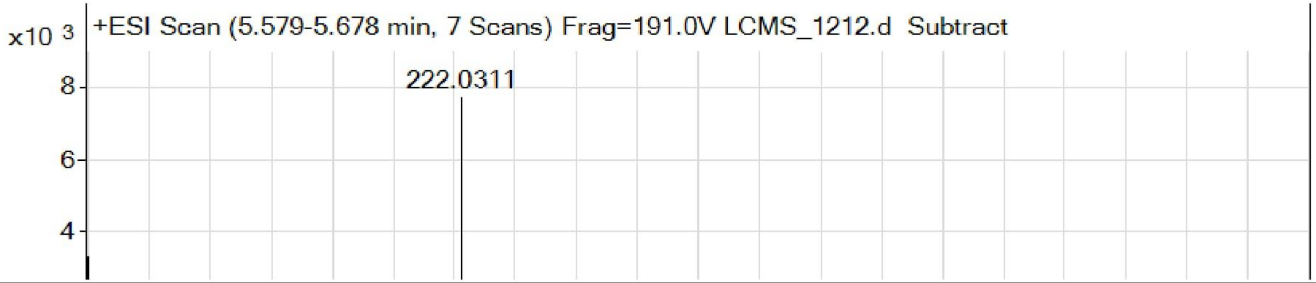

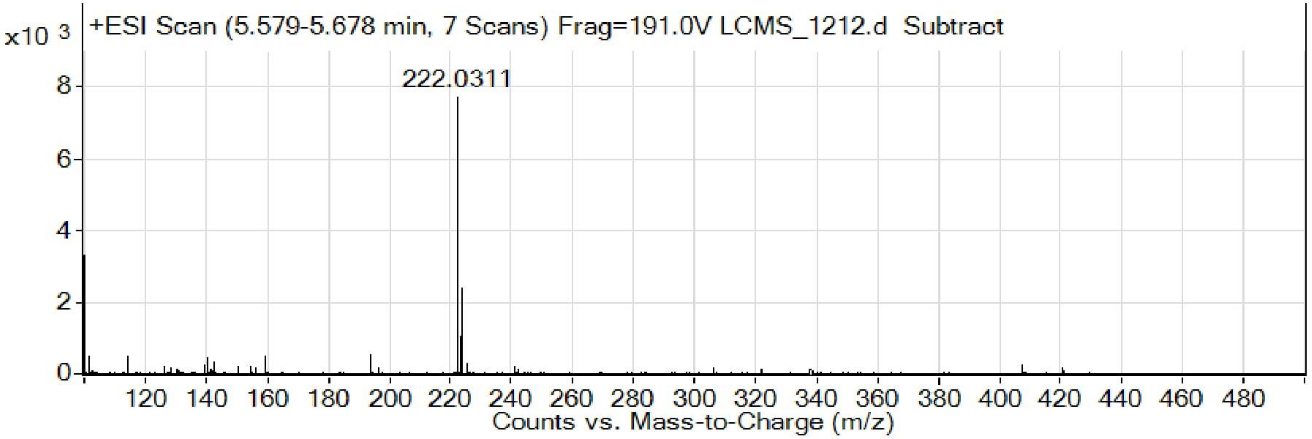

Peak List

m/z	z	Abund
59,0632		1734,92
100,1338		3402,5
222,0311	1	7987,29
223,0391	1	1056,36
224,0339	1	2442,91

--- End Of Report ---

Qualitative Analysis Report

**Data Filename**	LCMS_1213.d	**Sample Name**	#924
**Sample Type**	Sample	**Position**	Vial 6
**Instrument Name**	Instrument 1	**User Name**	Falaleev A.
**Acq Method**	MeOH-ACN-H2O 10-30-60.m	**Acquired Time**	19-Jan-16 4:57:43 PM
**IRM Calibration Status**	Success	**DA Method**	alex20150126.m
**Comment**	**Reaction MIX - 11 min (with 100 mkL AcOH)**		

Stream Name LC 1 **Acquisition SW Version** 6200 series TOF/6500 series Q-TOF B.06.01 (B6157)

User Chromatograms

Fragmentor Voltage 191 **Collision Energy** 0 **Ionization Mode** ESI

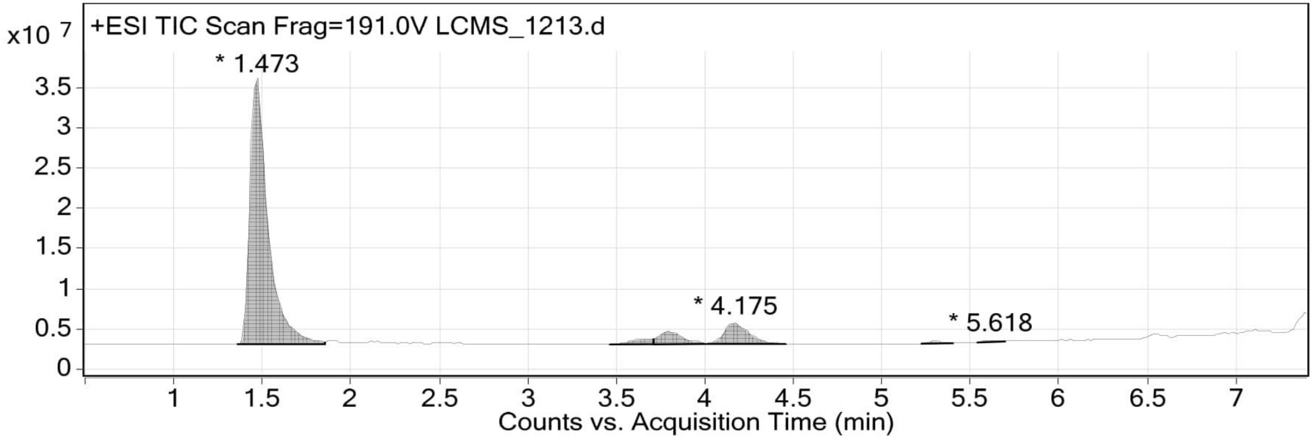

Integration Peak List

Peak	Start	RT	End	Height	Area	Area %	Area Sum, %
1	1,356	1,473	1,854	33082106	239433947	100	83,34
2	3,462	3,711	3,711	679078	5559259	2,32	1,93
3	3,711	3,794	4,01	1615652	14311806	5,98	4,98

4	4,01	4,175	4,457	2584340	25168023	10,51	8,76
5	5,22	5,286	5,402	255714	1646457	0,69	0,57
6	5,535	5,618	5,701	213998	1194068	0,5	0,42

User Spectra

Spectrum Source	Fragmentor Voltage	Collision Energy	Ionization Mode
Peak (1) in "+ TIC Scan"	191	0	ESI

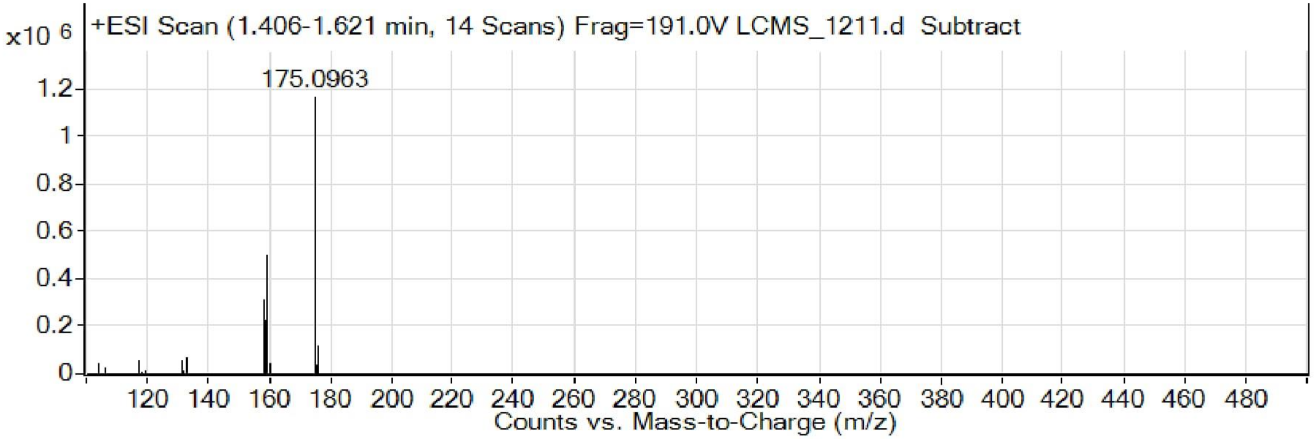

Peak List

m/z	z	Abund
158,1057		367944,09
159,1134		601732,9
175,0963	1	1387859,28
176,0239	1	144458,78

Spectrum Source	Fragmentor Voltage	Collision Energy	Ionization Mode
Peak (2) in "+ TIC Scan"	191	0	ESI

Qualitative Analysis Report

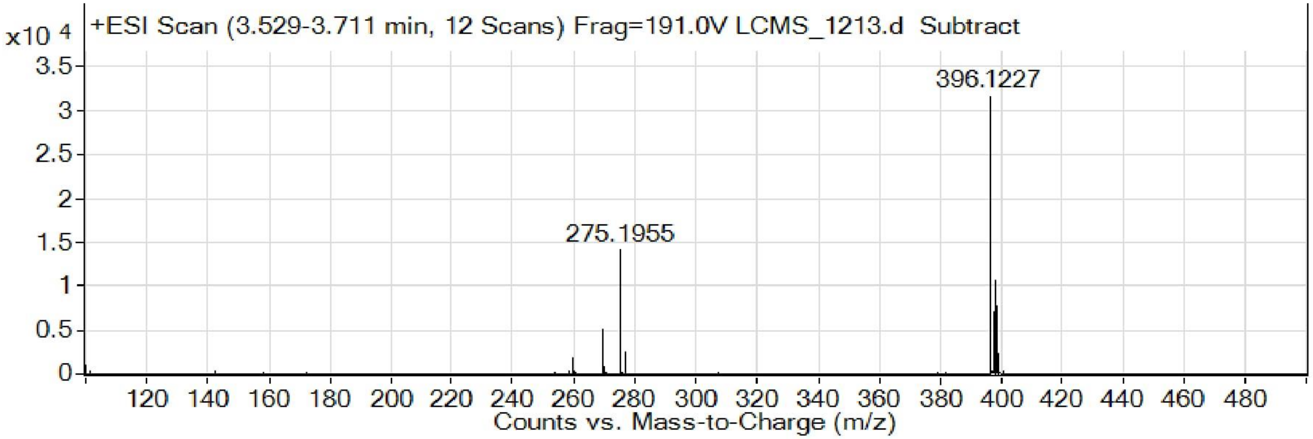

Peak List

m/z	z	Abund
269,1681		5276,93
275,1955		14205,41
396,1227	1	32801,82
397,1215	1	7425,68
398,1164	1	10875,07

Spectrum Source	Fragmentor Voltage	Collision Energy	Ionization Mode
Peak (3) in "+ TIC Scan"	191	0	ESI

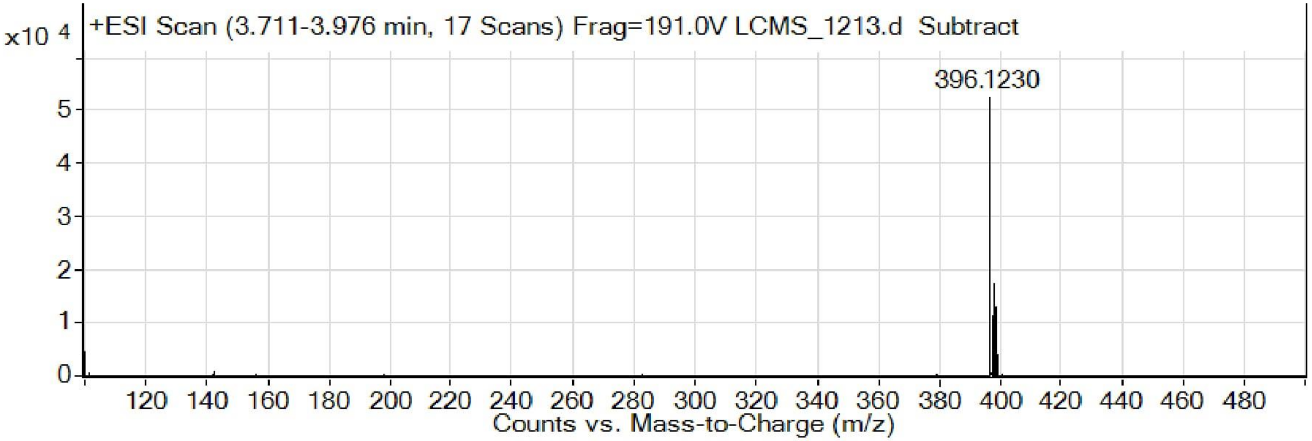

Peak List

m/z	z	Abund
396,1230	1	54563,8
397,1212	1	11705,64
398,1265	1	17815,54

Spectrum Source	Fragmentor Voltage	Collision Energy	Ionization Mode
Peak (4) in "+ TIC Scan"	191	0	ESI

Qualitative Analysis Report

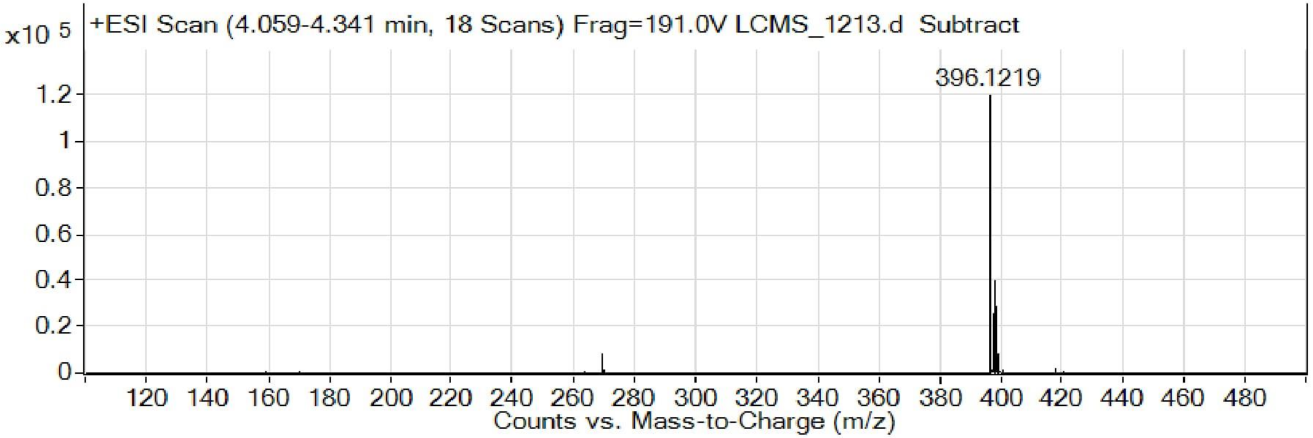

Peak List

m/z	z	Abund
396,1219	1	124010,97
397,1214	1	26643,8
398,1164	1	39962,49

Spectrum Source	Fragmentor Voltage	Collision Energy	Ionization Mode
Peak (5) in "+ TIC Scan"	191	0	ESI

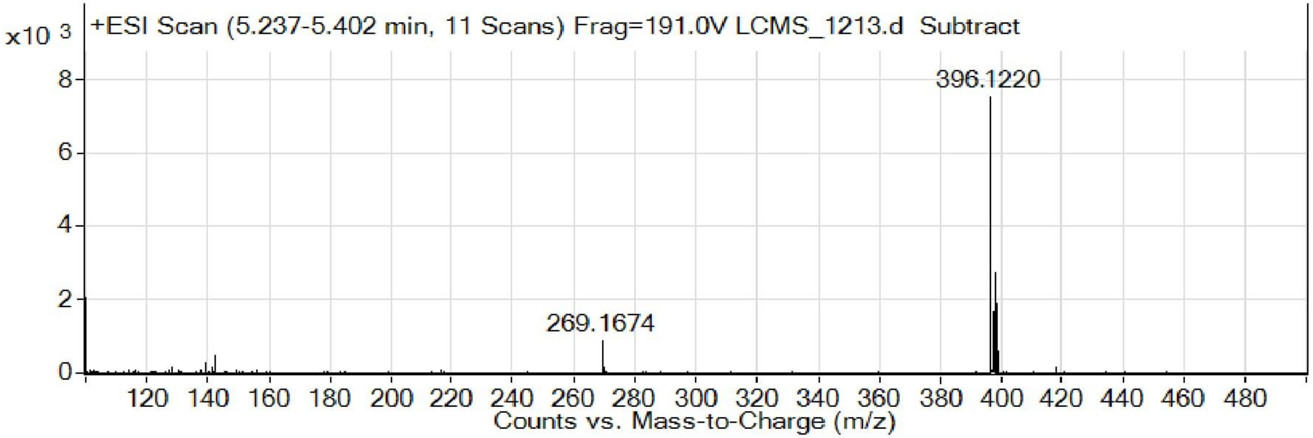

Peak List

m/z	z	Abund
59,0629		2006,99
100,1374		2060,36
269,1674		940,3
396,1220	1	7698,37
397,1207	1	1770,39
398,115	1	2731,23

Spectrum Source	Fragmentor Voltage	Collision Energy	Ionization Mode
Peak (6) in "+ TIC Scan"	191	0	ESI

Qualitative Analysis Report

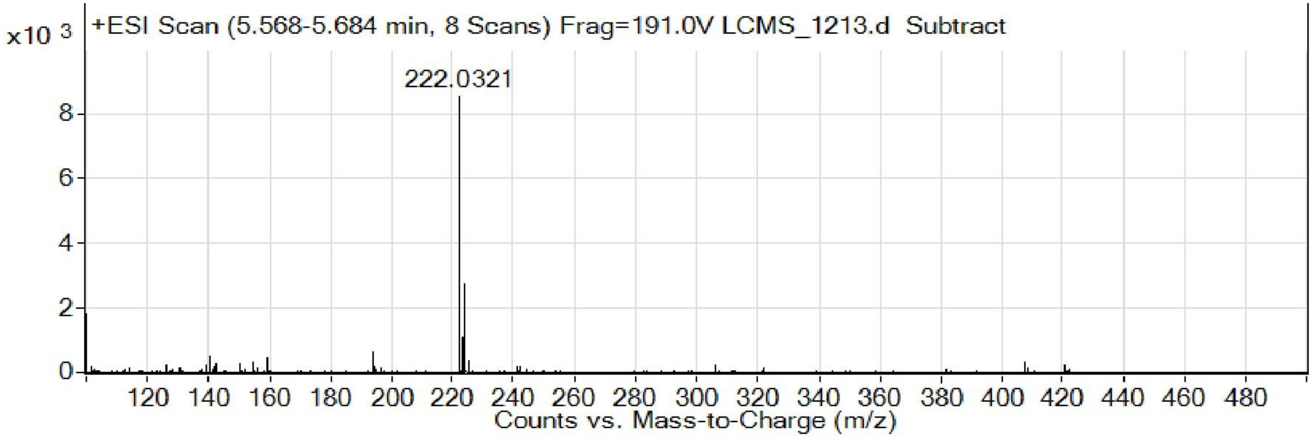

Peak List

m/z	z	Abund
59,0657		2036,51
100,1344		1931,73
222,0321	1	8880,27
223,0381	1	1127,96
224,0418	1	2783,09

--- End Of Report ---

Qualitative Analysis Report

**Data Filename**	LCMS_1214.d	**Sample Name**	#924
**Sample Type**	Sample	**Position**	Vial 7
**Instrument Name**	Instrument 1	**User Name**	Falaleev A.
**Acq Method**	MeOH-ACN-H2O 10-30-60.m	**Acquired Time**	19-Jan-16 5:14:53 PM
**IRM Calibration Status**	Success	**DA Method**	alex20150126.m
**Comment**	Reaction MIX - 16 min (with 100 mkL AcOH)		

Stream Name LC 1 **Acquisition SW Version** 6200 series TOF/6500 series Q-TOF B.06.01 (B6157)

User Chromatograms

Fragmentor Voltage 191 **Collision Energy** 0 **Ionization Mode** ESI

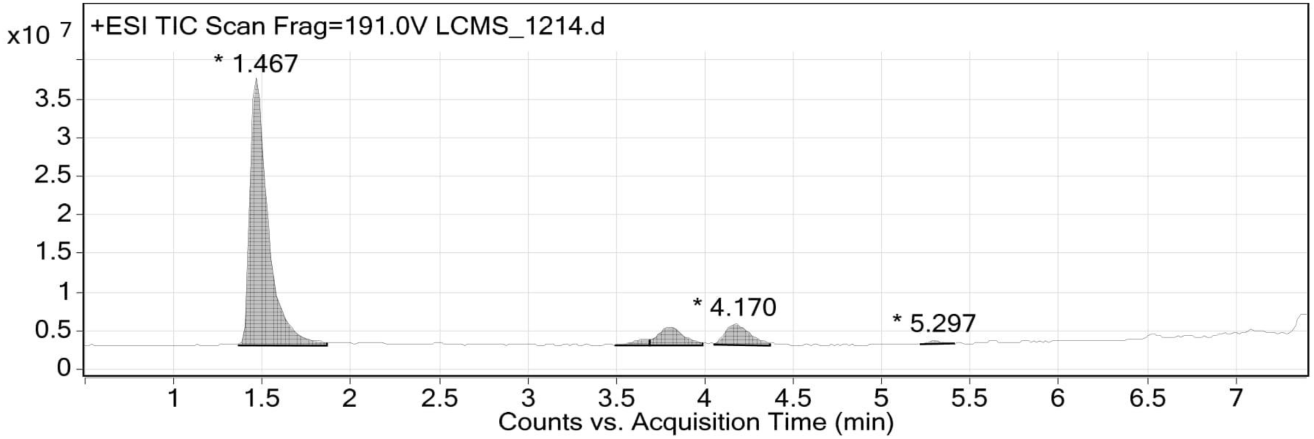

Integration Peak List

Peak	Start	RT	End	Height	Area	Area %	Area Sum, %
1	1,368	1,467	1,865	34622764	250734717	100	81,21
2	3,49	3,656	3,689	772548	5333592	2,13	1,73
3	3,689	3,805	3,987	2349490	24131258	9,62	7,82

4	4,054	4,17	4,369	2678470	26437019	10,54	8,56
5	5,215	5,297	5,414	371292	2096757	0,84	0,68

User Spectra

Spectrum Source	Fragmentor Voltage	Collision Energy	Ionization Mode
Peak (1) in "+ TIC Scan"	191	0	ESI

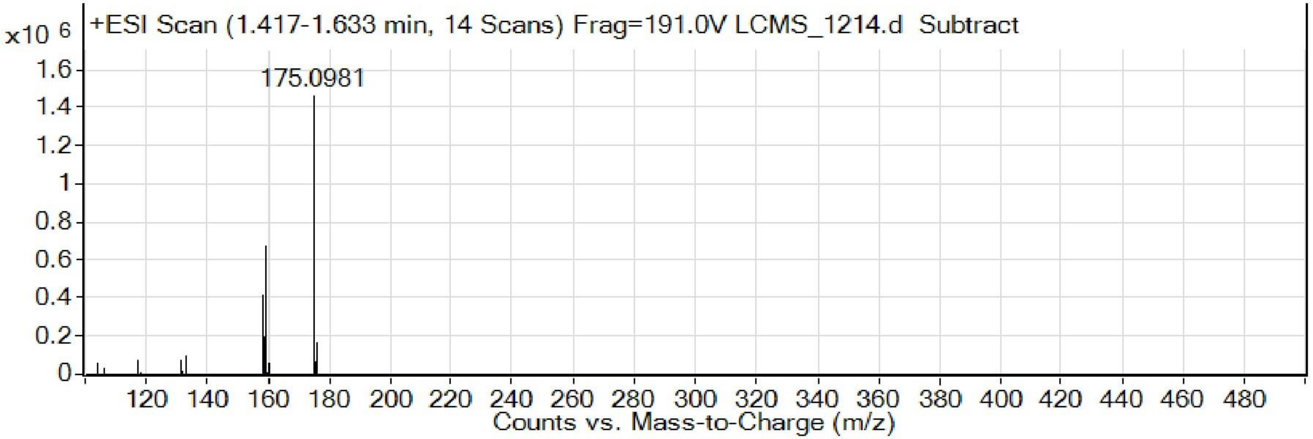

Peak List

m/z	z	Abund
158,1055		421217,3
159,1132		679374,63
175,0981	1	1521520,56
176,1187	1	164259,93

Spectrum Source	Fragmentor Voltage	Collision Energy	Ionization Mode
Peak (2) in "+ TIC Scan"	191	0	ESI

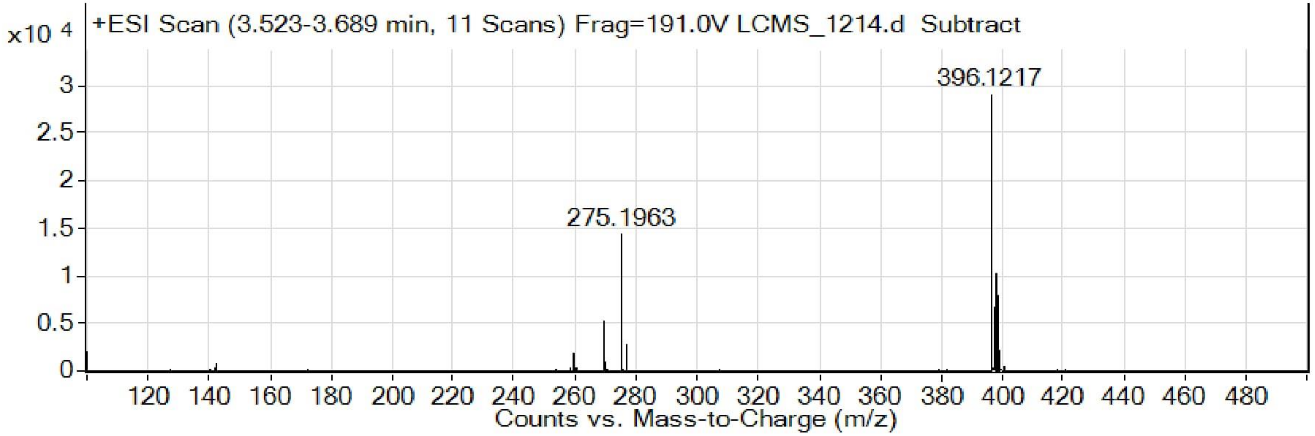

Peak List

m/z	z	Abund
269,1693		5444,53
275,1963		14382,8
396,1217	1	30588,51
397,1227	1	6917,96
398,2179	1	10312,08

Spectrum Source	Fragmentor Voltage	Collision Energy	Ionization Mode
Peak (3) in "+ TIC Scan"	191	0	ESI

Qualitative Analysis Report

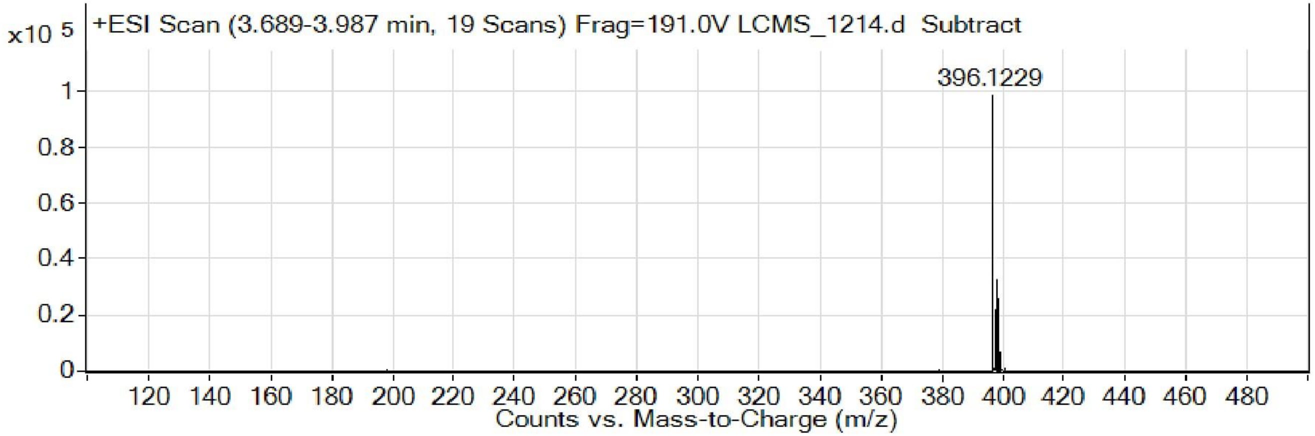

Peak List

m/z	z	Abund
396,1229	1	104359,36
397,1225	1	22665,74
398,2178	1	33263,58

Spectrum Source	Fragmentor Voltage	Collision Energy	Ionization Mode
Peak (4) in "+ TIC Scan"	191	0	ESI

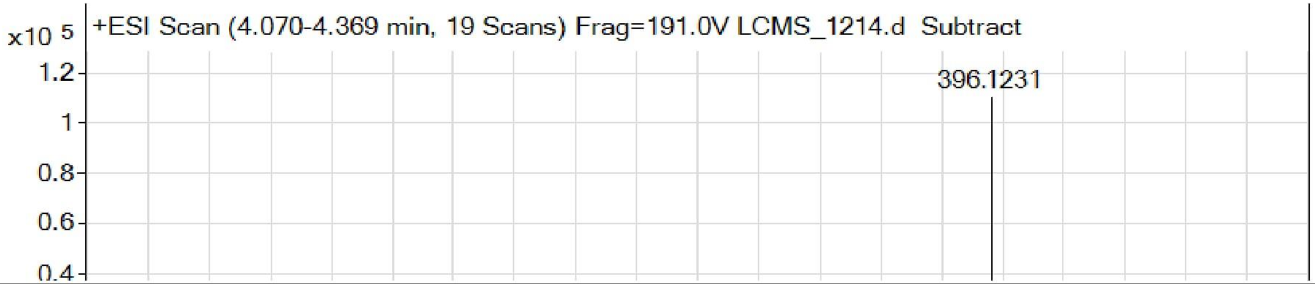

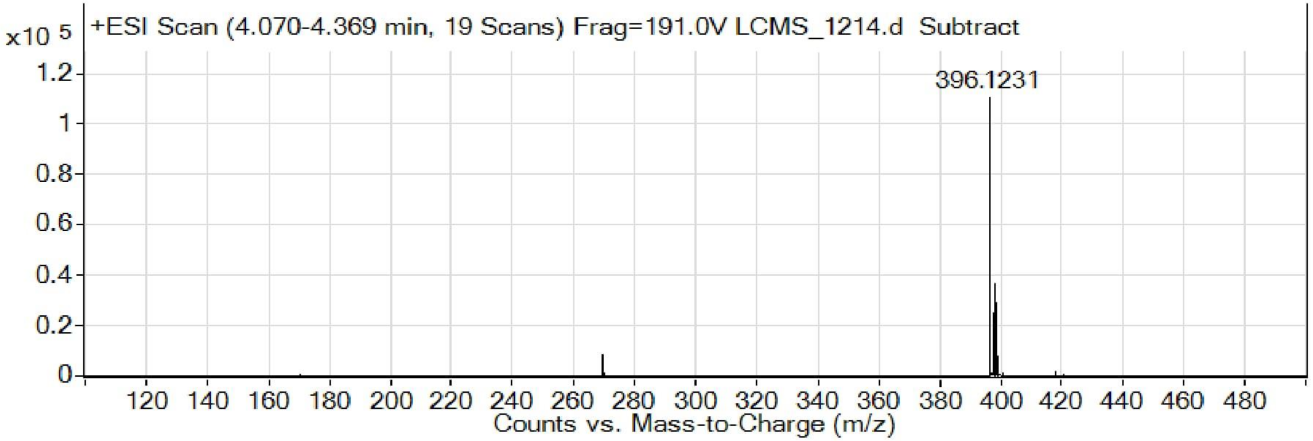

Peak List

m/z	z	Abund
396,1231	1	114652,6
397,1227	1	25332,02
398,2181	1	37252,7

Spectrum Source	Fragmentor Voltage	Collision Energy	Ionization Mode
Peak (5) in "+ TIC Scan"	191	0	ESI

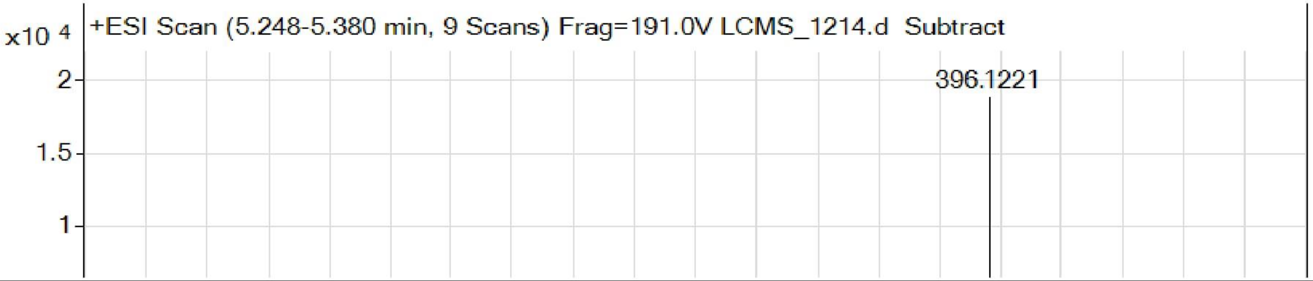

Qualitative Analysis Report

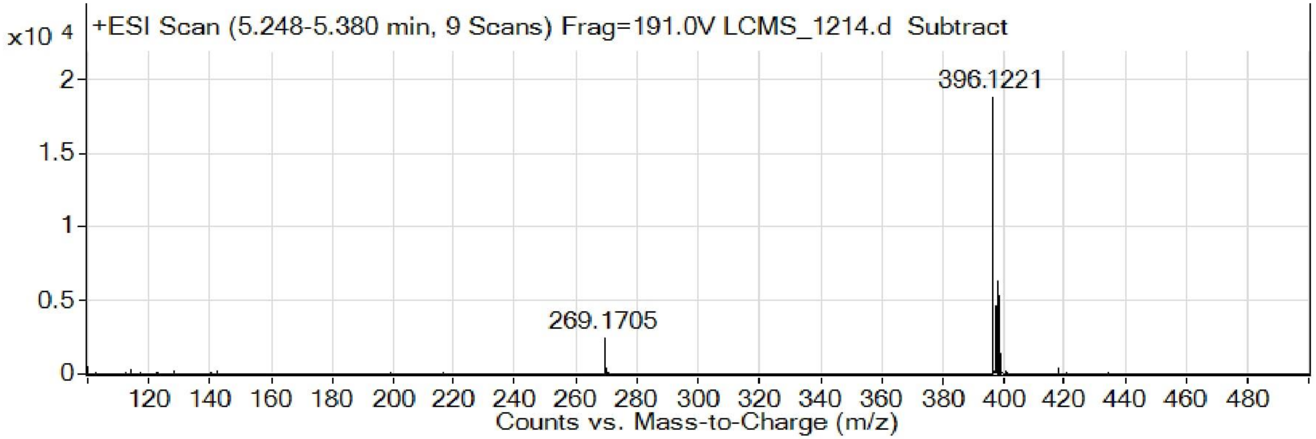

Peak List

m/z	z	Abund
269,1705		2516,05
396,1221	1	19344,08
397,1237	1	4636,85
398,2191	1	6579,87

--- End Of Report ---

Qualitative Analysis Report

**Data Filename**	LCMS_1215.d	**Sample Name**	#924
**Sample Type**	Sample	**Position**	Vial 8
**Instrument Name**	Instrument 1	**User Name**	Falaleev A.
**Acq Method**	MeOH-ACN-H2O 10-30-60.m	**Acquired Time**	19-Jan-16 5:30:33 PM
**IRM Calibration Status**	Success	**DA Method**	alex20150126.m
**Comment**	**Reaction MIX - 30 min (with 100 mkL AcOH)**		

Stream Name LC 1 **Acquisition SW Version** 6200 series TOF/6500 series Q-TOF B.06.01 (B6157)

User Chromatograms

Fragmentor Voltage 191 **Collision Energy** 0 **Ionization Mode** ESI

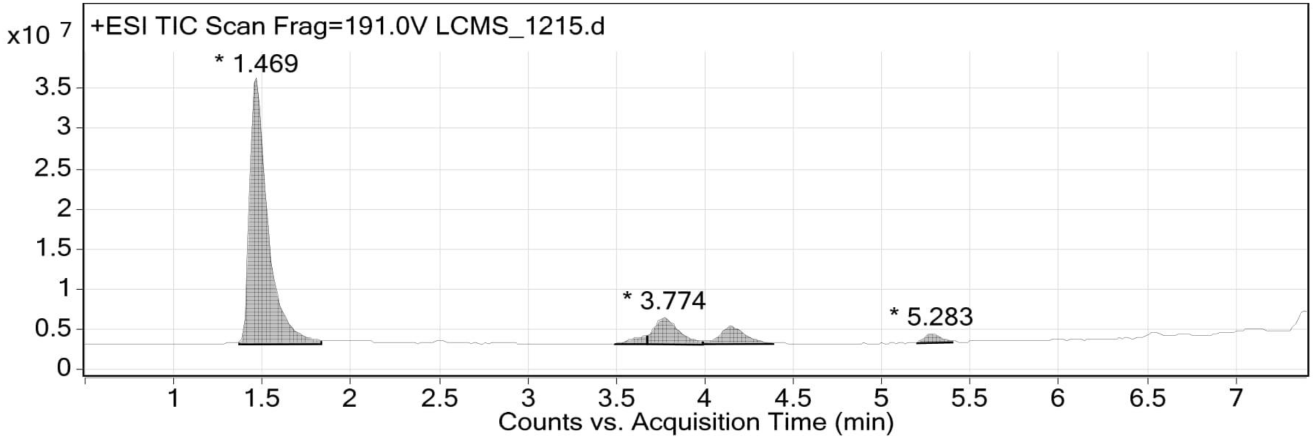

Integration Peak List

Peak	Start	RT	End	Height	Area	Area %	Area Sum, %
1	1,37	1,469	1,834	33217560	241718530	100	77,62
2	3,492	3,674	3,674	1074496	6675507	2,76	2,14
3	3,674	3,774	3,989	3336799	32660392	13,51	10,49

4	3,989	4,155	4,387	2267341	22968105	9,5	7,37
5	5,2	5,283	5,399	1115116	7409725	3,07	2,38

User Spectra

Spectrum Source	Fragmentor Voltage	Collision Energy	Ionization Mode
Peak (1) in "+ TIC Scan"	191	0	ESI

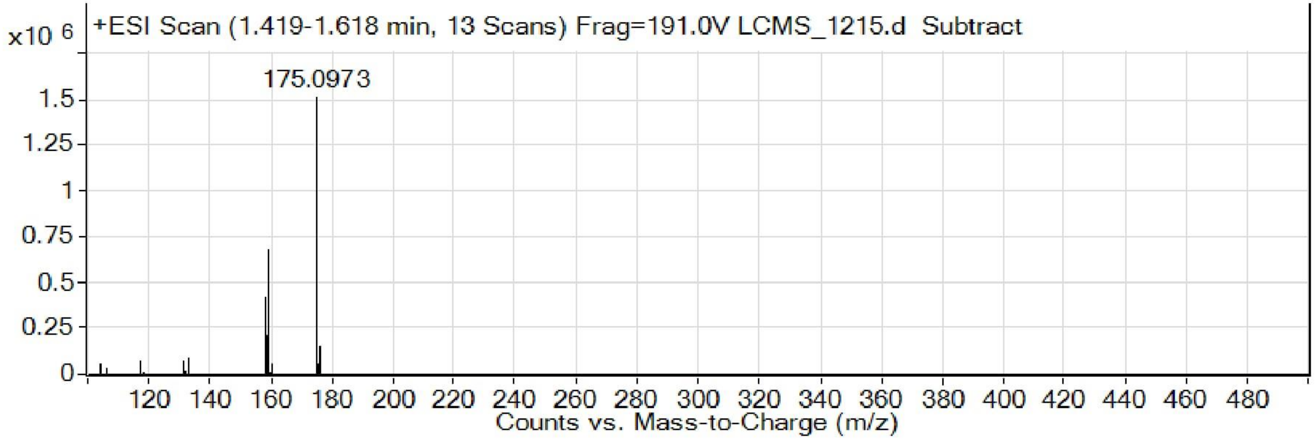

Peak List

m/z	z	Abund
158,1053		428231,71
159,113		691849,22
175,0973	1	1556913,49
176,0985	1	167758,33

Spectrum Source	Fragmentor Voltage	Collision Energy	Ionization Mode
Peak (2) in "+ TIC Scan"	191	0	ESI

Qualitative Analysis Report

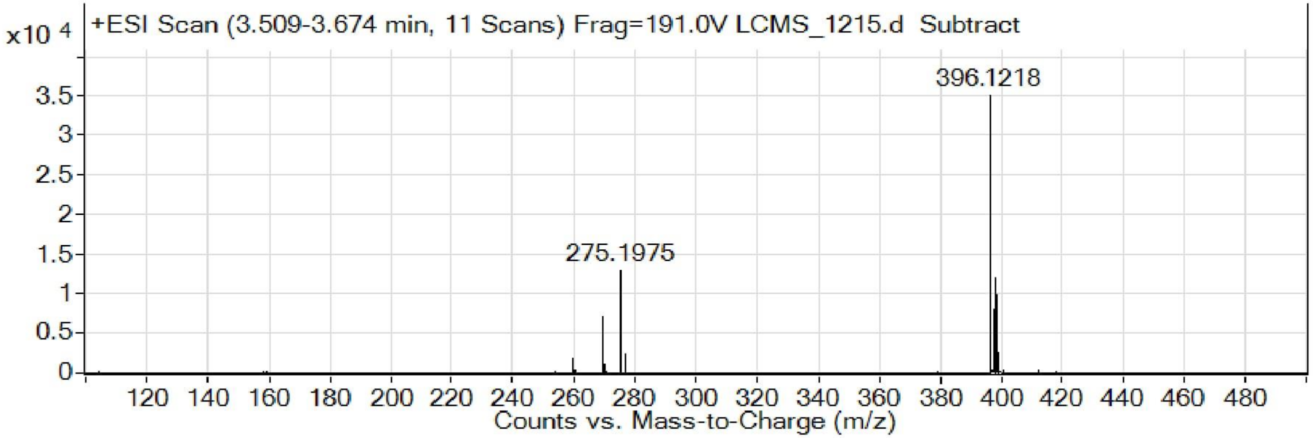

Peak List

m/z	z	Abund
269,1702		7159,94
275,1975		13107,44
396,1218	1	36024,64
397,1242	1	8178,69
398,2191	1	12466,68

Spectrum Source	Fragmentor Voltage	Collision Energy	Ionization Mode
Peak (3) in "+ TIC Scan"	191	0	ESI

Qualitative Analysis Report

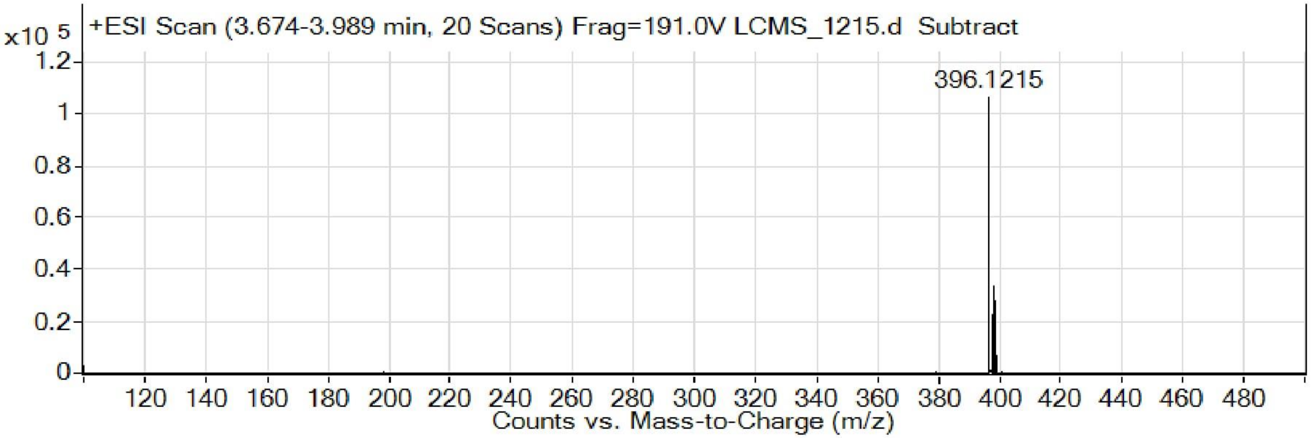

Peak List

m/z	z	Abund
396,1215	1	108265,17
397,124	1	23083,89
398,2192	1	34874,63

Spectrum Source	Fragmentor Voltage	Collision Energy	Ionization Mode
Peak (4) in "+ TIC Scan"	191	0	ESI

Qualitative Analysis Report

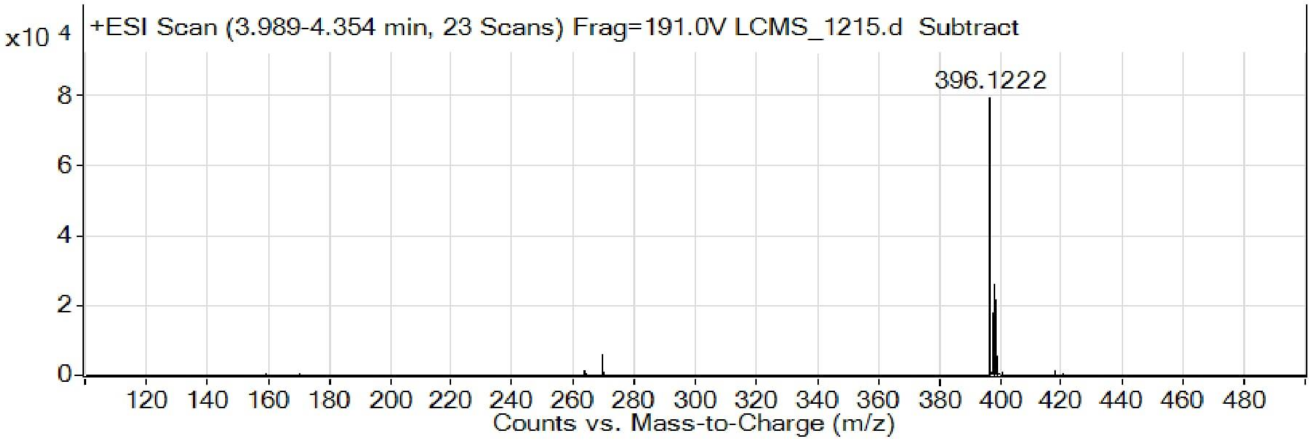

Peak List

m/z	z	Abund
396,1222	1	81039,59
397,1241	1	18046,5
398,2195	1	26880,01

Spectrum Source	Fragmentor Voltage	Collision Energy	Ionization Mode
Peak (5) in "+ TIC Scan"	191	0	ESI

Qualitative Analysis Report

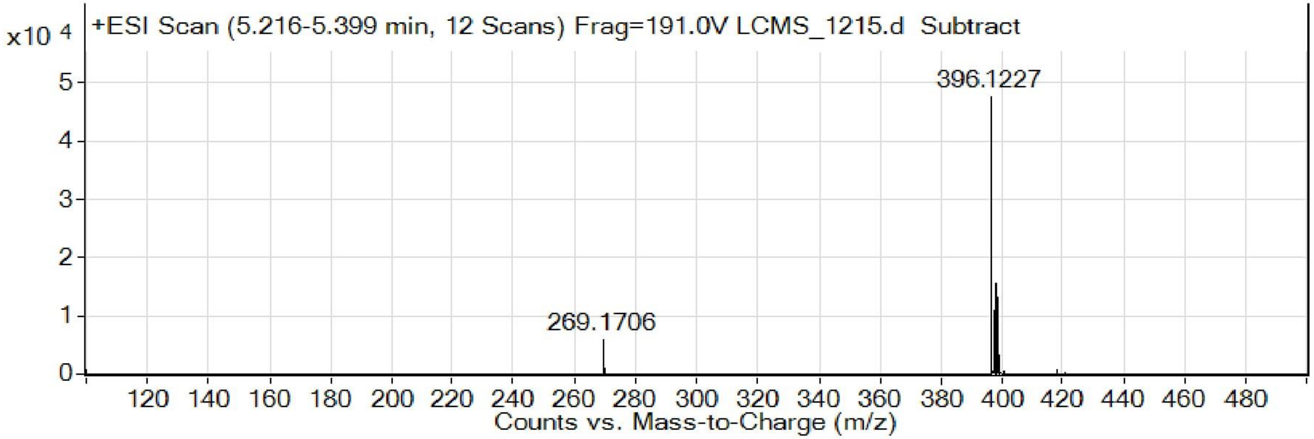

Peak List

m/z	z	Abund
269,1706		6036,46
396,1227	1	48429,09
397,1246	1	11065,25
398,2196	1	16049,19

--- End Of Report ---

Qualitative Analysis Report

**Data Filename**	LCMS_1216.d	**Sample Name**	#924
**Sample Type**	Sample	**Position**	Vial 9
**Instrument Name**	Instrument 1	**User Name**	Falaleev A.
**Acq Method**	MeOH-ACN-H2O 10-30-60.m	**Acquired Time**	19-Jan-16 5:47:33 PM
**IRM Calibration Status**	Success	**DA Method**	alex20150126.m
**Comment**	**Reaction MIX - 60 min (with 100 mkL AcOH)**		

Stream Name LC 1 **Acquisition SW Version** 6200 series TOF/6500 series Q-TOF B.06.01 (B6157)

User Chromatograms

Fragmentor Voltage 191 **Collision Energy** 0 **Ionization Mode** ESI

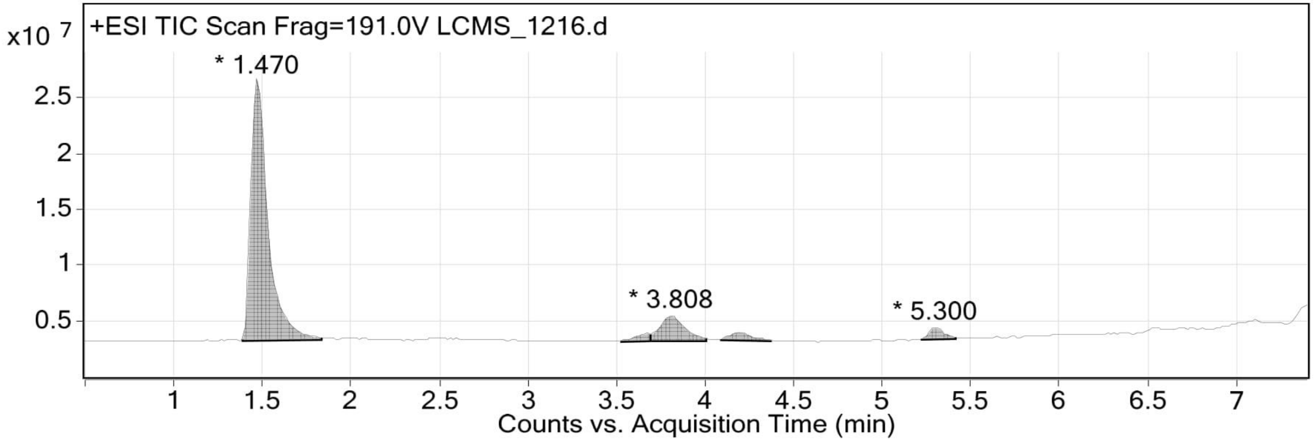

Integration Peak List

Peak	Start	RT	End	Height	Area	Area %	Area Sum,%
1	1,387	1,47	1,835	23559341	160098681	100	79,89
2	3,526	3,675	3,692	674084	4377803	2,73	2,18
3	3,692	3,808	4,007	2241163	22525296	14,07	11,24

4	4,09	4,189	4,372	757174	6908268	4,32	3,45
5	5,218	5,3	5,416	1009562	6476487	4,05	3,23

User Spectra

Spectrum Source	Fragmentor Voltage	Collision Energy	Ionization Mode
Peak (1) in "+ TIC Scan"	191	0	ESI

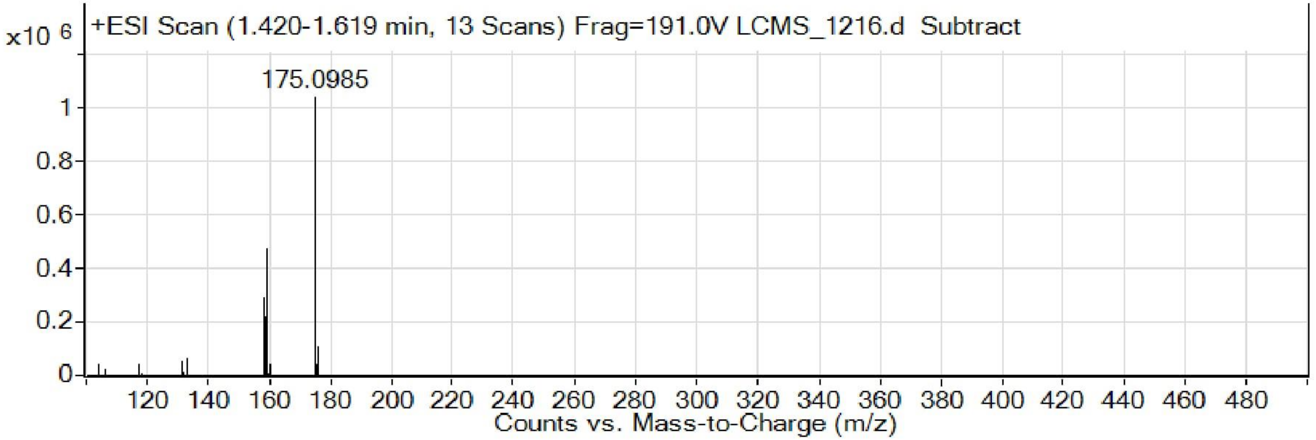

Peak List

m/z	z	Abund
158,106		291186,47
159,1138		475870,03
175,0985	1	1092603,84
176,0994	1	113969,02

Spectrum Source	Fragmentor Voltage	Collision Energy	Ionization Mode
Peak (2) in "+ TIC Scan"	191	0	ESI

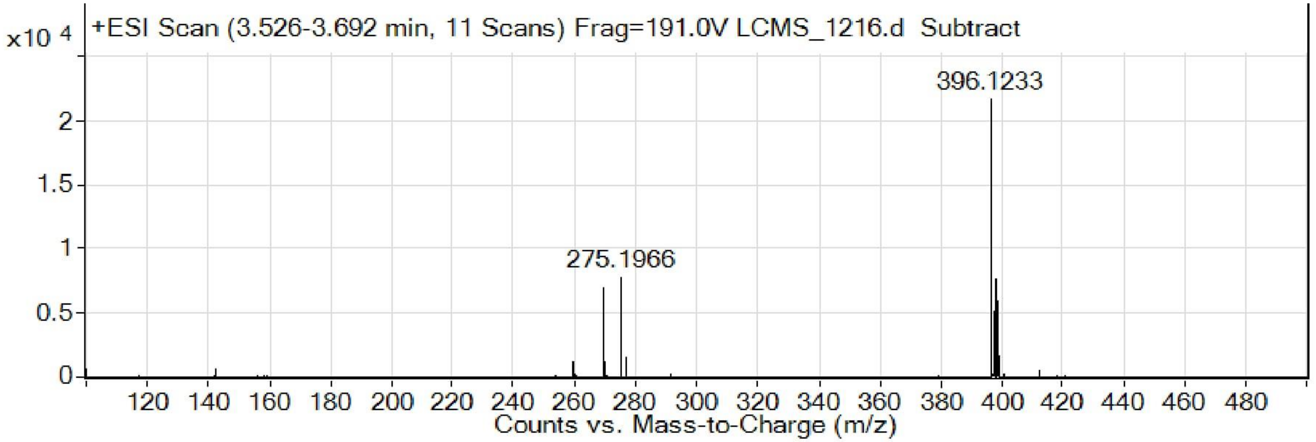

Peak List

m/z	z	Abund
59,061		2880,19
269,1694		7075,53
275,1966		7866,97
396,1233	1	22741,97
397,1224	1	5240,38
398,2179	1	7737,39

Spectrum Source	Fragmentor Voltage	Collision Energy	Ionization Mode
Peak (3) in "+ TIC Scan"	191	0	ESI

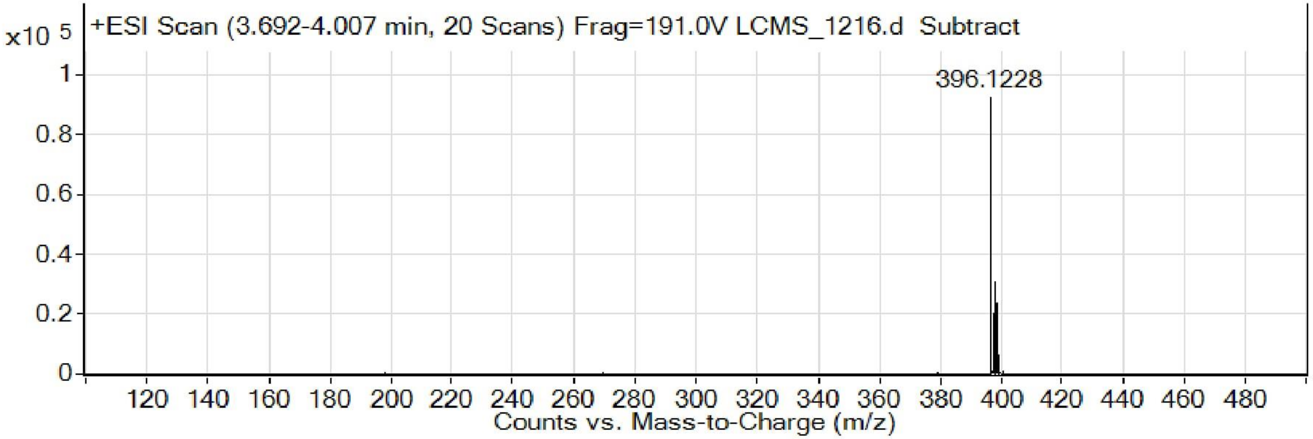

Peak List

m/z	z	Abund
396,1228	1	97293,86
397,1223	1	20845,21
398,2175	1	31360,73

Spectrum Source	Fragmentor Voltage	Collision Energy	Ionization Mode
Peak (4) in "+ TIC Scan"	191	0	ESI

Qualitative Analysis Report

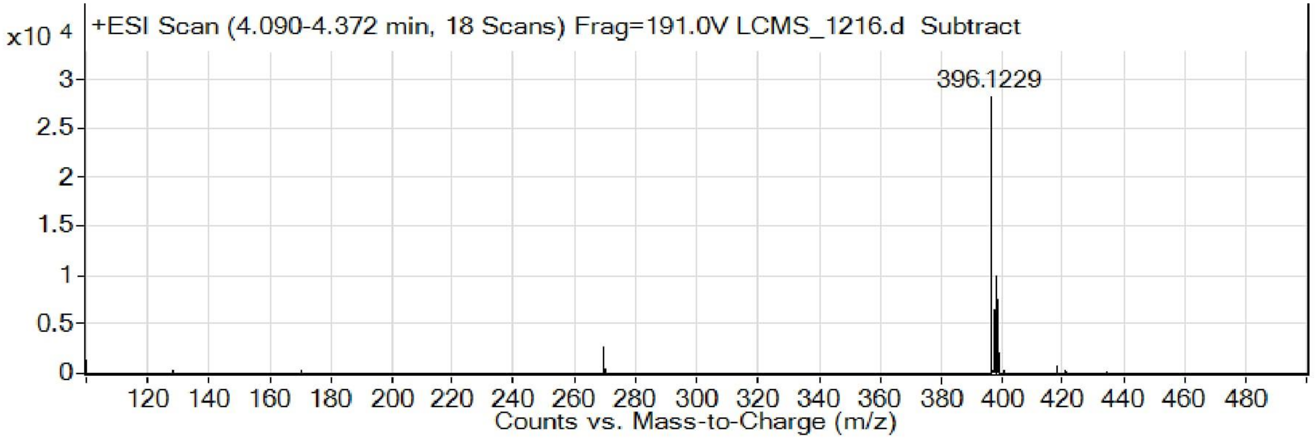

Peak List

m/z	z	Abund
396,1229	1	29583,18
397,122	1	6672,2
398,2175	1	9966,93

Spectrum Source	Fragmentor Voltage	Collision Energy	Ionization Mode
Peak (5) in "+ TIC Scan"	191	0	ESI

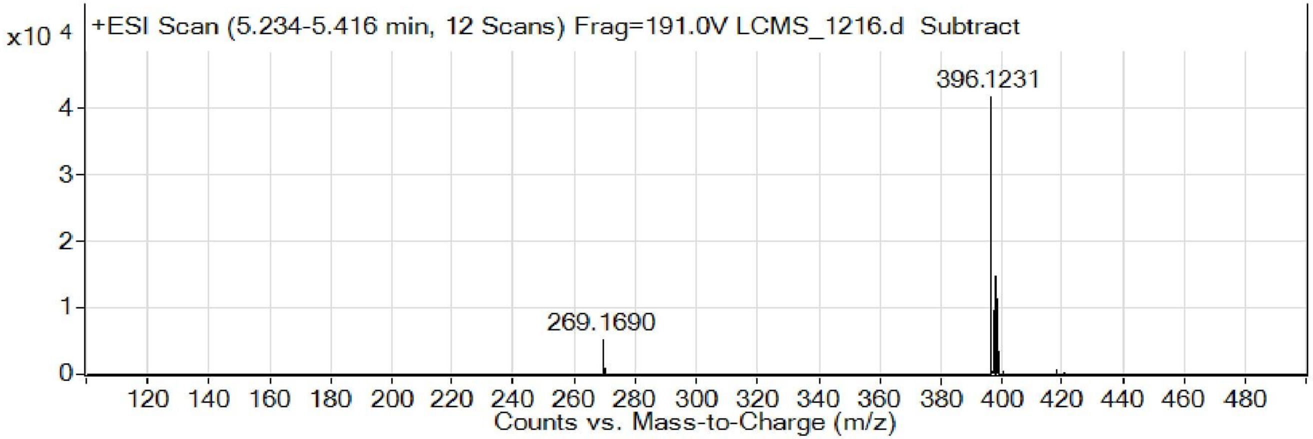

Peak List

m/z	z	Abund
269,169		5299,03
396,1231	1	43674,21
397,1222	1	9788,1
398,2177	1	14809,12

--- End Of Report ---
